# Classification of truck-involved crash severity: Dealing with missing, imbalanced, and high dimensional safety data

**DOI:** 10.1371/journal.pone.0281901

**Published:** 2023-03-22

**Authors:** Seyed Iman Mohammadpour, Majid Khedmati, Mohammad Javad Hassan Zada

**Affiliations:** 1 Civil Engineering Department, Sharif University of Technology, Tehran, Iran; 2 Department of Industrial Engineering, Sharif University of Technology, Tehran, Iran; 3 Department of Mobility Systems Engineering, Technical University of Munich, Munich, Germany; Nanjing Forestry University, CHINA

## Abstract

While the cost of road traffic fatalities in the U.S. surpasses $240 billion a year, the availability of high-resolution datasets allows meticulous investigation of the contributing factors to crash severity. In this paper, the dataset for Trucks Involved in Fatal Accidents in 2010 (TIFA 2010) is utilized to classify the truck-involved crash severity where there exist different issues including missing values, imbalanced classes, and high dimensionality. First, a decision tree-based algorithm, the Synthetic Minority Oversampling Technique (SMOTE), and the Random Forest (RF) feature importance approach are employed for missing value imputation, minority class oversampling, and dimensionality reduction, respectively. Afterward, a variety of classification algorithms, including RF, K-Nearest Neighbors (KNN), Multi-Layer Perceptron (MLP), Gradient-Boosted Decision Trees (GBDT), and Support Vector Machine (SVM) are developed to reveal the influence of the introduced data preprocessing framework on the output quality of ML classifiers. The results show that the GBDT model outperforms all the other competing algorithms for the non-preprocessed crash data based on the G-mean performance measure, but the RF makes the most accurate prediction for the treated dataset. This finding indicates that after the feature selection is conducted to alleviate the computational cost of the machine learning algorithms, bagging (bootstrap aggregating) of decision trees in RF leads to a better model rather than boosting them via GBDT. Besides, the adopted feature importance approach decreases the overall accuracy by only up to 5% in most of the estimated models. Moreover, the worst class recall value of the RF algorithm without prior oversampling is only 34.4% compared to the corresponding value of 90.3% in the up-sampled model which validates the proposed multi-step preprocessing scheme. This study also identifies the temporal and spatial (roadway) attributes, as well as crash characteristics, and Emergency Medical Service (EMS) as the most critical factors in truck crash severity.

## 1. Introduction

According to a survey conducted in 2017, 71.6% of the total value of commodities are transported by trucks within the U.S. [1]. Trucks are an integral part of the U.S. economy, and they are also responsible for the economic burden of severe crashes. Although the frequency of truck crashes is relatively low, it is still an important issue because a crash involving one or multiple trucks is obviously a large-scale one. Large trucks comprise only 4% of the registered vehicles in the country, but account for 8% of all fatal crashes [2]. The need to enhance heavy vehicle traffic safety has been a major social concern for decades in the U.S. which necessitates achieving a clear picture of the contributing factors to crash severity in order to map proper countermeasures.

### 1.1 Machine learning and crash severity analysis

The underlying factors of crash severity can be detected and identified via two approaches, including statistical and Machine Learning (ML) methods. The statistical methods are known for easy interpretation and their solid theoretical basis. However, they impose restrictive assumptions on data distribution and the link function between the target variable and regressors. If the basic assumptions of the statistical approach are violated, the estimated parameters will be biased [3]. In addition, the flexibility of these parametric approaches is limited when modeling high dimensional safety data due to the inherent collinearity between variables, which in turn drives scholars to eliminate a number of predictors to preserve the interpretability of estimated parameters. Therefore, some potential contributing factors might be overlooked, and the model’s predictive power decreases. Instead, machine learning models do not rely on specific functional forms and strict assumptions on data distribution. Furthermore, the ML algorithms propose simple solutions for complex crash data issues, including unobserved heterogeneity, missing values, imbalanced classes, multicollinearity, and high dimensionality. The recent progress in the field of artificial intelligence has provided a bright outlook on the application of data mining techniques and new data sources in crash severity analysis to obtain new insights into the crash mechanism. For example, researchers have applied interpretable text mining algorithms on crash narratives to explore crash severity risk factors [4]. Exploring the potential correlation between various risk factors and revealing that how their coupling mechanisms influence the crash severity is also a new topic introduced by association rule mining algorithms [5, 6]. With the ability to solve complex prediction problems, advanced ML methods have been also used for real-time crash severity prediction [7]. Despite much effort put into overcoming the limitations of conventional statistical methods, the cutting-edge statistical models are more arduous to interpret and use in practice. Dealing with big data in a high-dimensional space and complex nonlinear associations has shifted attention from statistical methods to machine learning algorithms.

Previous studies indicated that the relationships between the crash contributing factors and crash severity does not necessarily follow a fixed linear link function. Yang et al. [8] explored the existing connections between the built environment factors and truck-involved crashes in the Los Angeles region, using Zero Inflated Poisson (ZIP) statistical model and XGBoost (eXtreme Gradient Boosting) machine learning algorithm. Their results signified that the derived effects of the risk factors in crash severity are not consistent in the two adopted approaches. The SHAP (SHapley Additive exPlanation) value plots illustrated the nonlinear nature of the associations, confirming the results of the XGBoost. Hence, machine learning algorithms can search for more accurate and case-specific links between the independent and dependent variables through relaxing the oversimplified assumption of certain paradigm relationships in the statistical approaches [8]. Moreover, recent studies illustrated the significance of the interaction effect of road and environment characteristics on the severity of traffic crashes [9]. While statistical models need some adjustments to extract these interaction effects, most ML models can automatically take them into account. In contrast to this finding, most previous studies on the truck-involved crash severity analysis have employed classical discrete choice methods, e.g. logistic regression rather than state-of-the-art machine learning classifiers.

### 1.2 Data preprocessing

Whereas the quality of the model outputs is highly dependent on the quality of the input data based on the “Garbage In Garbage Out (GIGO)” principle [10], the application of appropriate data mining tools is essential to improve data quality, and gain valuable knowledge. The GIGO principle is a concept in data modeling and analytics where, based on this principle, in a processing system, the quality of the data generated by the system cannot be higher than that of the data fed into it. Therefore, a ML algorithm yields misleading results if it is working on defective data. In this regard, the missing values and imbalanced characteristics of crash severity data should be treated in the preprocessing stage. There are two primary approaches to missing data treatment, including the Complete-Case analysis (CC) and Missing data Imputation (MI) [11]. The first approach drops all crash observations with missing values on any of the features, while MI forecasts the missing values given the conditional distribution of the observed data for the factors with missing information. The CC approach diminishes the prediction power of classification models by discarding a significant volume of useful information considering the sparse distribution of missing cells. This is especially highlighted when the study targets specific types of crashes with few observations.

The available datasets on truck-involved crashes consist of numerous attributes which usually contain missing values. Looking at the literature, most previous studies have not discussed their strategies to address the missing data, but some of them have adopted the complete case analysis (CC) approach. Nonetheless, the missing information is shown to be distributed non-uniformly among different severity levels [12]. Indeed, some variables systematically include more missing values for crash records with specific severity outcomes. Thus, removing incomplete instances can flaw the sample in addition to reducing the sample size, leading to biased parameter estimates and erroneous inferences [12]. In this way, the MI technique would be the right choice for handling the missing data especially when analyzing particular crash types with relatively small samples.

Being an intrinsic characteristic of many multi-label datasets, the class imbalance problem is a common pitfall in crash severity data analytics where multiple attribute levels are distributed in different proportions over the data space [13]. As classification models aim to maximize the overall accuracy, the minority classes (severe crashes) are overwhelmed by the majority ones. In other words, the developed classifiers represent high overall accuracy but low recall values for the minority classes. Since the main purpose of research studies is to uncover and delve into the influential factors on severe crash outcomes, such models fail to be informative in reality. Two approaches to cope with class imbalance are: (1) Application of the models that implicitly tackle this issue, and (2) Application of sampling methods in the preprocessing stage. In the first approach, the precision and recall values are utilized to penalize the neglect of minority classes during training [14], or cost-sensitive learning schemes are implemented that take the misclassification cost into consideration [15]. In the second approach, oversampling the minority class [16] or under-sampling the majority class [17] is performed during the pretraining phase. However, the literature focuses mainly on the analysis of specific crash types with small sample sizes, and so, it is not recommended to apply the under-sampling scheme. A recent study done by Fiorentini and Losa [17] reviewed 16 papers on crash severity analysis, and found out that only two studies had accounted for class imbalance, despite its key role in eliminating erroneous minority class predictions. Besides, most previous studies on truck-related crash severity have overlooked the class imbalance issue. As a consequence, the established classifier would make fallacious severity forecasts for the target severe outcome crashes, and draw invalid conclusions on the critical contributing factors to crash injury severity.

The high dimensional feature space in crash datasets results in a large amount of unnecessary computational costs, especially when applying advanced machine learning models. Dimensionality reduction is another important step in preprocessing of big data. Modern safety data warehouses encompass multitudinous features for each observed crash. To decline the computational cost and discard irrelevant variables, the number of features should be reduced. Feature selection and feature extraction are the most widespread approaches for this purpose. The feature extraction techniques such as the Principal Component Analysis (PCA) extract a few combined (and in the case of PCA, orthogonal) components from the preliminary variables that produce a representative variance of the raw data [18]. They are not the appropriate tools when the study intends to discover interpretable influential factors on severe crashes because by this means, the original variables lose their identity via being merged into a smaller set of new variables with latent meanings. Accordingly, studies on crash severity mostly utilize supervised feature selection algorithms. Among the most common systematic feature selection techniques in the traffic safety literature is the random forest feature importance method. This method keeps only the most significant factors influencing the severity of the crashes, and excludes the less representative ones. Even though only a few variables explain a large variation in the target variable (e.g., crash severity) [19], most recent studies on the truck-involved crash severity have not dropped the irrelevant variables in the preprocessing stage. Moreover, the few studies which discarded less important attributes took non-systematic approaches. As a result, there is a need for the application of systematic dimensionality reduction (feature selection) techniques in the injury severity analysis of truck-involved crashes.

### 1.3 Literature review

The previous research on the crash severity classification concentrated on the impacts of road-environmental and human factors as well as crash characteristics on injury severity using statistical and ML methods [12, 20–25] while the effects of vehicle-related features and Emergency Medical Service (EMS) on crash severity outcomes has been usually overlooked [26–29]. There is also a limited number of studies that concentrate mainly on truck-involved crash severity. Zhu and Srinivasan [12] explored the contribution of crashes, vehicles, and drivers’ specifications to the severity of truck-involved crash events. They found out that driver distraction, alcohol use, and emotional factors are associated with higher crash severities. Hosseinzadeh et al. [25] employed the mixed logit and Support Vector Machine (SVM) models to predict the large truck crash severity. The results highlighted the effects of truck drivers’ fatigue and deviation to the left as the most influential predictors. In addition, the SVM algorithm outperformed the statistical model in terms of accuracy. Moreover, studies also investigated hazardous material truck crashes, and the results suggested that gender, weekday, rural highways, and illumination are related to crash severity [30]. Naik et al. [31] concluded that air temperature, rain humidity, and wind speed are associated with single-vehicle truck crash injury severity. Ahmed et al. [32] reported that the severity level of truck crashes was slightly higher on icy roads and snowy weather conditions. Zou et al. [33] investigated the association between spatiotemporal variables and truck-involved crash severity. Based on their results, the multi-vehicle crashes were more severe in the afternoon, while the single-vehicle crashes tended to be less severe at the same time. The recent studies on risk factors of truck-involved crash severity are detailed in Table 1. Various studies compared statistical models including multinomial logit, mixed logit, ordered probit model, ordered logit, and logistic regression with ML algorithms including RF, Gradient-Boosted Decision Trees (GBDT), Adaptive Boosting (AdaBoost), SVM, K-Nearest Neighbors (KNN), Decision Tree (DT), Artificial Neural Network (ANN) with different architectures, and XGBoost in terms of prediction accuracy, where the better performance of ML techniques in crash severity classification was concluded [27, 34–39]. Moreover, the RF and ANN models often had better predictive performance than their other ML counterparts although there is no rule of thumb for model selection, and the results of the previous research might be data-specific.

**Table 1:** Studies of the risk factors of truck-involved crash severity.

Authors	Risk factors	Model types	Treatments for: missing values/class imbalance/feature reduction
Zhu and Srinivasan [12]	Driver, crash and vehicle characteristics	Ordered probit model	replaced with dummies/no treatment/applied but not discussed
Chang and Chien [40]	Driver, road, environment, crash and vehicle characteristics	CART	CC approach/no treatment/not applied
Osman et al. [41]	Road, environment and crash characteristics	Multinomial logit, nested logit, ordered logit, and generalized ordered logit	no discussed treatment/no treatment/ not applied
Naik et al. [31]	Driver, road, environment and crash characteristics	Mixed ordered logit and mixed multinomial logit	no discussed treatment/no treatment/ not applied
Uddin and Huynh [30]	Driver, road, environment, crash and vehicle characteristics	Mixed logit	no discussed treatment/no treatment/ not applied
Zou et al. [33]	Driver, road, environment, crash and vehicle characteristics	Spatial generalized ordered probit and mixed ordered probit	no discussed treatment/no treatment/ applied based on previous research and intuitive considerations
Zheng et al. [19]	Driver, road, environment, crash and vehicle characteristics	GBDT	no treatment/no treatment /The authors exclude irrelevant, privacy variables and redundant variables
Azimi et al. [42]	Driver, road, environment, crash and vehicle characteristics	Mixed ordered logit	no discussed treatment/no treatment/ not applied
Li et al. [38]	Driver, road, environment and crash characteristics	Mixed logit, GBDT, AdaBoost and RF	no discussed treatment/no treatment /variables were selected based on their categories, their correlations between each other, their relationship to the dependent variable, and the quality of data
Haq et al. [43]	Driver, road, environment and crash characteristics	Bayesian binary logit models with both fixed- and random-effects	CC approach/no treatment/not applied
Zhou et al. [44]	Driver, road, environment, crash and vehicle characteristics	Multinomial logistic regression, NB, CART, SVM, and XGBoost	no discussed treatment/over-sampling, under-sampling, a hybrid method, and a cost-sensitive learning method/applied but not discussed
Hosseinzadeh et al. [25]	Driver, road, environment, and crash characteristics	Mixed logit and SVM	CC approach/no treatment/not applied
Tahfim and Yan [45]	Road, environment, crash and vehicle characteristics	K-prototypes clustering-based GBDT	CC approach/no treatment/applied but not discussed
Song and Fan [46]	Driver, road, environment and crash characteristics	LCA clustering-based ordered logit	no discussed treatment/no treatment/ applied but not discussed

AdaBoost: Adaptive Boosting; GBDT: Gradient-Boosted Decision Trees; SVM: Support Vector Machine; CC: Complete-Case analysis; RF: Random Forest; XGBoost: eXtreme Gradient Boosting; CART: Classification and Regression Tree; NB: Naïve Bayes; LCA: Latent class analysis

Tang et al. [47] developed a two-layer ensemble learning model, in which the first layer combines the prediction power of RF, AdaBoost, and GBDT models, while the second layer completes the classification based on a logistic regression. The proposed model outperformed the SVM, MLP, and RF algorithms. Some research studies also developed hybrid clustering-based algorithms to deal with the inherent unobserved heterogeneity within the crash data [18, 45]. Few studies applied deep learning algorithms for crash severity analysis [14], where the forecasts made by deep neural networks were more veracious than those of other ML algorithms. However, Abdulhafedh [48] pointed out that this class of models may represent a black box with random nature since they do not provide any perspicuous interpretation of their estimated parameters. Shiran et al. [49] developed crash severity models using a variety of ML techniques, and identified the key factors having the largest impact on the collision severity for the state highways in California, USA via a sensitivity analysis. The adopted models included the multinomial logistic regression, artificial neural network multi-layer perceptron, and two decision tree techniques (CHi-square Automatic Interaction Detector (CHAID) and C5.0). The outcome of the sensitivity analysis revealed that the decision tree C5.0 had the highest reliability among all models, introducing the cause of the crash and the number of vehicles as the best-performing predictors for the crash severity classification. Hence, in spite of representing a high level of accuracy, the complexity of artificial neural networks which is accompanied by high computational costs and lack of parameter interpretability has reduced their chance of becoming the dominant models.

### 1.4 Motivation and objectives

Based on a thorough review of the research literature, there are few studies that have used advanced MI techniques to impute missing values in the traffic safety field [11, 50, 51]. In addition, unfortunately, there is an evident trend toward neglecting the class imbalance problem in truck-involved crash severity analysis as elaborated by Table 1. This has led to classifiers which are not capable of predicting high severity classes accurately. Besides, the utilization of systematic dimensionality reduction (feature selection) techniques has not been investigated yet in the severity classification of truck-involved crashes. Overall, despite its crucial influence on the validity of the results, data cleansing and preprocessing has not gained sufficient attention in the traffic safety literature. Hence, this study aims to introduce a comprehensive multi-step preprocessing framework to deal with missing values, class imbalance, and high dimensionality issues. The proposed scheme is then employed to the dataset for the Trucks Involved in Fatal Accidents in 2010 (TIFA 2010) which suffers from the missing, imbalanced, and high dimensional data. In contrast to the most case studies selected by the traffic safety literature, the TIFA 2010 dataset also contains the data regarding the vehicle-related features and Emergency Medical Service (EMS) as well as the other typical variables for crash severity prediction. Thus, this paper provides novel insights into the contributing factors to truck-involved crash severity. In the end, as there is a wide range of ML algorithms, and no concrete conclusion has been achieved on the most appropriate classifier for crash severity forecasting, this research also performs a comparative analysis of a variety of ML models, including lazy methods, deep learning, tree-based bagging/boosting ensemble learning, and support vector machines. Consequently, this study adds to the body of knowledge on the application of data mining in crash severity analysis by addressing the aforementioned research gaps.

## 2. Methodology

The data preprocessing scheme is carried out prior to applying the ML algorithms to the TIFA 2010 dataset. In the pretraining stage, the dataset is first cleansed from irrelevant, duplicated, and invariant features. The variables with more than 10% of missing values are also discarded to preclude biased inferences from low-quality data. Considering the limited sample size of the target crash type for analysis, the remaining missing values are imputed using a decision tree-based algorithm to preserve the available information. As the next step in the preprocessing, oversampling the minority classes is conducted by the synthetic minority oversampling technique (SMOTE) for data balancing. In the end, the high dimensional feature space is searched by the random forest algorithm to find the variables with the most discriminative power in classifying the crash severity levels. Employing the gain ratio impurity index while using the RF feature importance technique, 30 of the most influential features are selected out of the total 110 relevant attributes. In this regard, there exist three datasets for further analysis, including the original dataset after data cleansing and MI (N = 3643), the balanced dataset with all variables (N = 6576), and the balanced dataset with the selected 30 features (N = 6576). To assess and compare the performance of different classifiers in crash severity prediction, five ML classification models are developed in this paper. The following subsections elaborate on the selected case study, data preprocessing, modeling, and performance evaluation.

### 2.1 Data description

The Trucks Involved in Fatal Accidents (TIFA) survey was carried out by the Center for National Truck and Bus Statistics at the University of Michigan Transportation Research Institute [52]. The TIFA 2010 dataset contains records for all the medium and heavy trucks involved in fatal traffic crashes in the 50 states of the U.S. as well as the District of Columbia during the calendar year of 2010. The target variable in this study was the truck occupant injury severity represented by the KABCO scale, where the letters K, A, B, C, and O stand for fatal injury, incapacitating injury, non-incapacitating injury, possible injury, and property damage only (PDO), respectively. The severity class label attributes are aggregated into three levels, including KA (fatal and severe injury), BC (less severe injury), and O (PDO). The TIFA dataset contains all the variables from the Fatality Analysis Reporting System (FARS) data, including the crash variables, the vehicle variables (for the truck), and the occupant variables (for the driver of the truck). While the FARS data covers detailed information on the crash environment and events, the information on the vehicles involved is limited. This gap is bridged by a comprehensive telephone survey. The TIFA survey collected detailed descriptions of the trucks; that is, the cab style of the power unit; gross vehicle weight rating and gross combination weight rating; trailer type, cargo body style, cargo type, the company type, and operating authority of the operator of the vehicle [52]. Also, the survey added detailed human-related factors such as the truck driving experience and driver’s workload. The TIFA 2010 dataset consists of 3643 crash events and 190 variables, and can be fully accessed at the webpage of the U.S. National Highway Traffic Safety Administration (NHTSA) [53]. The tabular data is presented in standard SAS format and is available via the link provided in the Supporting information section of the paper (See S1 File). The description of the whole 190 variables are detailed in the TIFA codebook provided by the University of Michigan Transportation Research Institute [52]. This study explores the entire dataset attributes without inducing any particular restrictions to provide a comprehensive perspective of the influential factors on truck-involved crash severity.

### 2.2 Data preprocessing

In the data cleansing stage, the maximum acceptable ID-ness, stability, and missing values criteria are utilized to eliminate low-quality attributes for further analysis. The ID-ness identified data columns in which nearly all values (⩾98%) are different. Since such variables do not represent valuable information e.g., ID numbers, they should be dropped. Also, the stability criterion eliminated attributes with the same values for at least 90% of the observations because these variables account for minor variations in the data. Besides, the variables with over 10% of missing values are also discarded as the results derived from the incomplete features would be biased due to a large share of missing information [54]. On the other hand, some variables make no sense to predict crash severity or duplicate the class label attribute. Therefore, they are also discarded before the model development, such as driver’s race, number of fatalities/injuries in the crash, occupant deaths at the crash scene, etc. At last, there remained only 110 attributes for the crash severity analysis out of 190 variables in the preliminary dataset.

The preprocessing scheme begins with missing data imputation. 48 features among the remaining attributes have less than 10% of missing values. The Decision tree-based Missing value Imputation (DMI) algorithm, introduced by Rahman and Islam [55], is employed. This method firstly partitions the entire dataset *D*_*Full*_ into two sub-datasets, one containing observations with missing values (*D*_*Miss*_) and the other containing complete data records (*D*_*Complete*_). Afterward, the DMI algorithm develops decision trees on (*D*_*Complete*_) taking the attributes which are missing in *D*_*Miss*_ as the class labels. Then, it assigns each observation in *D*_*Miss*_ to the leaf to which it belongs in order to make a single prediction on the missing value. Finally, the DMI method imputes the categorical missing value using the majority class voting within the leaves.

Since the crash severity levels are highly imbalanced, the SMOTE is implemented to balance severity classes, and obviate the minority class underrepresentation. The SMOTE is an over-sampling technique that increases the minority class instances by creating synthetic examples rather than over-sampling with replacement [56]. The SMOTE generates the new examples along the lines between a randomly chosen record of minority class and its K-nearest neighbors of the same class instead of simply duplicating less frequent observations. Depending on the amount of over-sampling needed, the parameter K as the number of the randomly sampled nearest neighbors is determined. The new examples are synthetized in the following way: Subtract the considered feature vector from its nearest neighbor, multiply the subtraction result by a random weight between 0 and 1, and add it to the feature vector of the considered example. This locates the new synthetic example along the line segment between the two specific actual observations, and thus, boosts the resolution for the decision space of the minority classes. Besides, it prevents the classifier from overfitting. A more detailed explanation on the SMOTE process is explained by Chawla et al. [56].

The final step in data preprocessing is the feature selection which declines computational costs, especially when dealing with big data. The RF feature importance algorithm applies the information gain ratio criterion to select the most influential 30 factors on truck-involved crash severity. The selected attributes are illustrated in Table 2. More details on the coding of the variables are reported in the TIFA codebook 2010 [52]. All the computations in this paper are conducted in RapidMiner Studio version 9.10.

**Table 2:** The selected explanatory variables.

Variable	Description	Min, Max	Mean (SD)
**crash date—month**	1–12	1, 12	6.79 (3.37)
**case state**	State in which the crash occurred	Rhode Island, Texas	-
**crash time—hour**	0–23	0, 23	11.72 (6.51)
**crash date—day**	1–31	1, 31	15.61 (8.74)
**transported to medical facility by**	8 categories (EMS Ground, EMS Air, etc.)	Not Reported, Not Transported	-
**number of person forms**	Number of persons involved in crash	1, 56	2.89 (2.25)
**roadway function class**	6 Rural and 6 Urban classes	Unknown Rural, Principal Arterial-Other	-
**manner of collision**	9 categories (Front-to-rear, Front-to-front, Angle, etc.)	Rear-to-rear, Angle	-
**route signing**	8 classes (Interstate, U.S. Highway, State Highway, etc.)	Frontage Road, State Highway	-
**number of vehicle forms**	Number of motor vehicles on-transit involved in crash	1, 15	2.14 (1.20)
**national highway system (NHS)**	Binary (yes/no)	No, yes	-
**day of week**	1–7	1 (Sunday), 7 (Saturday)	4.12 (1.78)
**speed limit**	18 categories	5 mph, 80 mph	56.3 (13.16)
**trafficway description**	8 categories (One-Way Trafficway, Two-Way-Not Divided, etc.)	Non-Trafficway Area, Two-Way, Not Divided	-
**vehicle make**	15 companies	Iveco/Magirus, Freightliner	-
**model year**	1991–2011	1991, 2011	2002 (5.98)
**land use**	land use–FHWA classification	Urban area, Rural area	-
**registration state**	Vehicle registration State	Guam, Multiple State Registration	-
**vehicle make-model**	Over 40 categories	NA, International Harvester/Navistar medium/heavy—cab behind engine	-
**relation to junction—junction**	11 categories (Non-Junction, Intersection-Related, etc.)	Acceleration/Deceleration Lane, Non-Junction	-
**roadway grade**	8 categories (Non-Trafficway Area, Level, Hillcrest, etc.)	Non-Trafficway Area, Level	-
**total lanes in roadway**	9 categories (Non-Trafficway Area, One lane, Two lanes, etc.)	Seven or more lanes, Two lanes	-
**light condition**	6 categories (Daylight, Dark—Not Lighted, Dark—Lighted, etc.)	Dark—Unknown Lighting, Daylight	-
**roadway surface type**	8 categories (Concrete, Brick or Block, etc.)	Brick or Block, Blacktop, Bituminous, or Asphalt	-
**cargo body type**	18 categories (Cargo Tank, Van/Enclosed Box, etc.)	Bus, Van/Enclosed Box	-
**atmospheric conditions**	10 categories (Clear, Rain, Snow, etc.)	Blowing Sand, Clear	-
**registered vehicle owner**	8 categories (Driver (in this crash) were Registered Owner, etc.)	Vehicle was Stolen, Vehicle Registered as Business/Company/Government Vehicle	-
**body type**	20 categories (Standard pickup, Truck-tractor, etc.)	Unknown body type, Truck-tractor	-
**areas of impact—initial damage**	23 categories (Left-Front Half, Right-Front Half, etc.)	Top, 12 Clock Point	-
**extent of damage**	4 categories (No Damage, Minor Damage, etc.)	No Damage, Disabling Damage	-

The “max” and “min” values in the third column for categorical variables denote the most and the least frequent categories. NA: not applicable due to multiple categories with the same frequency.

### 2.3 Support vector machine (SVM)

The SVM algorithm maps the explanatory feature vector, *x*_*i*_, into a high-dimensional feature space, using a kernel function *∅*(*x*_*i*_), and then, constructs an optimal hyperplane in the feature space by maximizing the margin among the linear decision boundaries; which can be employed for classification. In fact, optimal hyperplanes are useless when the training set is not linearly separable. Kernel machines can represent complicated decision boundaries that match any training set. But this is not very wise when the problem is very noisy. Cortes and Vapnik [57] show that noisy problems are best addressed by allowing some observations to violate the margin constraints in the primal problem. These potential violations are represented using positive slack variables *ξ*_*i*_. An additional parameter *C* controls the compromise between large margins and small margin violations [58]. Moreover, *w* and *b* define the decision hyperplane, which separates positives from negatives, {*x* | *w*^*T*^ *x* + *b* = 0}. Accordingly, *w* is perpendicular to the hyperplane. In addition, |*w*_*i*_| is the weight of the corresponding feature dimension: if *w*_*i*_ = 0, that feature is ignored, and if |*w*_*i*_| is high, that feature is important to the SVM’s decision. Based on these explanations, in binary classification, given a training set of input vector-class label pairs of (*x*_*i*_, *y*_*i*_), the SVM classifier solves the following optimization problem [57]:

$$\underset{w,b,\xi }{\min}\frac{1}{2}w^{T}w+C\sum_{i=1}^{N}\xi_{i}$$ (1)

subject to:

$$y_{i}(w^{T}\phi (x_{i})+b)\geq 1-\xi_{i};\;i=1,\;...\;,\;N$$ (2)

$$\xi_{i}\;\geq \;0;\;i=1,\;...\;,\;N$$ (3)

where *w* is the weight vector to define the decision boundary between classes, *ξ*_*i*_ is the slack variable measuring misclassification errors, *C* shows the penalty parameter, ∅(*x*_*i*_) is the Kernel function to map data from *X* space to a multi-dimensional *Z* space, *b* is the intercept term associated with decision boundaries, *y*_*i*_ is the class label for *i*^*th*^ observation, *i* is a counter variable, *N* is the sample size, and *T* is the matrix transpose operator.

The quadratic optimization problem for the estimation of SVM parameters can be solved by Lagrange multipliers. Among several Kernel functions, this study adopted the commonly used Gaussian kernel function. The discussed SVM binary classifier can be extended to solve multi-class classification problems using one-vs-one strategy based on a voting approach. This study developed three SVM models, using only two classes, including severe injury vs. PDO, severe injury vs. less severe injury, and PDO vs. less severe injury. To forecast a new observation, all the models are employed to generate votes for the three classes. Then, the class with the most votes is selected as the predicted class label for that observation.

### 2.4 K-nearest neighbor (KNN)

The KNN is a well-known machine learning algorithm for classifying the target variable based on its nearest training samples in the problem space. The KNN is a lazy learning algorithm where the function is approximated locally, and all computations are delayed until classification. The target variable is classified by a majority vote of its neighbors, with the variable being assigned to a class that is the most common among its *k* nearest neighbors (*k* is a small positive integer value) [59]. To find the nearest neighbors, a distance function should be used. The Euclidean distance, interpreted as the physical distance between two spatial points, is adopted as the distance function in this study. In addition, the optimal value of *k* is set to one after an iterative performance evaluation of various plausible choices.

### 2.5 Random forest (RF)

The RF model is a bagging (bootstrap aggregating) ensemble learning algorithm [60]. Ensemble learning is a meta-approach to machine learning that achieves superior performance via combining the predictive power of multiple weak machine learners. The RF grows multiple Decision Trees (DT) with independently and identically distributed random vectors on bootstrapped data from the original training dataset. The classification process within each tree is based on minimizing the impurity index at each node. Hence, in each node, one variable from a subset of randomly selected attributes is selected which better divides the class labels into homogenous subsets. The process continues until the stopping condition for node impurity is reached or the maximum allowable tree depth is violated. Each tree assigns a vote for the target variable. Finally, the class label attribute for each Out-Of-Bag (OOB) observation is predicted based on the majority vote of trees. The random selection of attributes at each node mitigates the multicollinearity issue when some attributes are highly correlated. These random properties of the RF model also preserve it from the overfitting issue. There are two hyperparameters for the RF model to be set, including the number of trees to grow and the number of attributes sampled at each node as candidate splitters.

The feature importance is a by-product of the training process of the RF algorithm. The RF ranks the variables’ importance in predicting the target variable using a weight function (*W*_*v*_). The weight is calculated for each feature based on the cumulative frequency and the discriminative value of that feature (*v*) in splitting the class label attribute across all trees. The formulation is presented in Eqs (4)–(5) [61]:

$$W_{v}=\sum_{k=1}^{K}\sum_{l=1}^{L}I(V_{k}^{l},v)\times \Delta i_{v}(l,k)$$ (4)

$$I(V_{k}^{l},v)={\{}_{0,}^{1,}{\;}_{otherwise}^{if\;\;V_{k}^{l}=v}$$ (5)

where *W*_*v*_ is the importance or weight of variable *v*, *I*(⋅) is an indicator function, *l* and *k* are two counter variables, *K* represents the number of trees, *L* indicates the number of nodes of the *k*^*th*^ tree, $V_{k}^{l}$ denotes the feature related to the node *l*, and *Δi*_*v*_ (*l*, *k*) is the decrease in impurity index resulting from the node *l* split in the *k*^*th*^ tree using the variable *v*.

The normalized weights based on the information gain ratio as the impurity index are utilized for the feature importance analysis and feature selection.

### 2.6 Gradient boosting decision tree (GBDT)

The GBDT is a boosting ensemble learning algorithm that seeks to “boost” the accuracy of decision trees sequentially, where each weak classifier is trained based on the error of a previous weak learner [62, 63]. A GBDT model can be considered as the expansion of a series to approximate the actual functional relationship, which is represented as follows [62]:

$$f(x)=\sum_{n}f_{n}(x)=\sum_{n}\beta_{n}g(x,\gamma_{n})$$ (6)

where *x* denotes the set of regressors, n is a counter variable, *f*(*x*) is the approximation of the class label attribute, and *β*_*n*_ represents the coefficients that are estimated by minimizing a loss function. In addition, *g*(*x*, *y*_*n*_) is each single decision tree where *γ*_*n*_ stands for the split variables.

The loss function is a prediction performance measure that is optimized using a gradient descent algorithm. The GBDT is sensitive to overfitting, especially without proper hyperparameter tuning. The tree complexity and learning rate have been allocated as the regularization parameters of the model, which both should be tuned to avoid overfitting issues. The learning rate regularizes the speed of updating after each iteration. A small learning rate results in a higher computational cost, and in return, a better forecast accuracy would be achieved. Moreover, the tree complexity indicates the number of nodes of each single decision tree. A grid search is implemented to select the hyperparameters of the GBDT model.

### 2.7 Multi-layer perceptron neural network (MLP)

**Fig 1:**
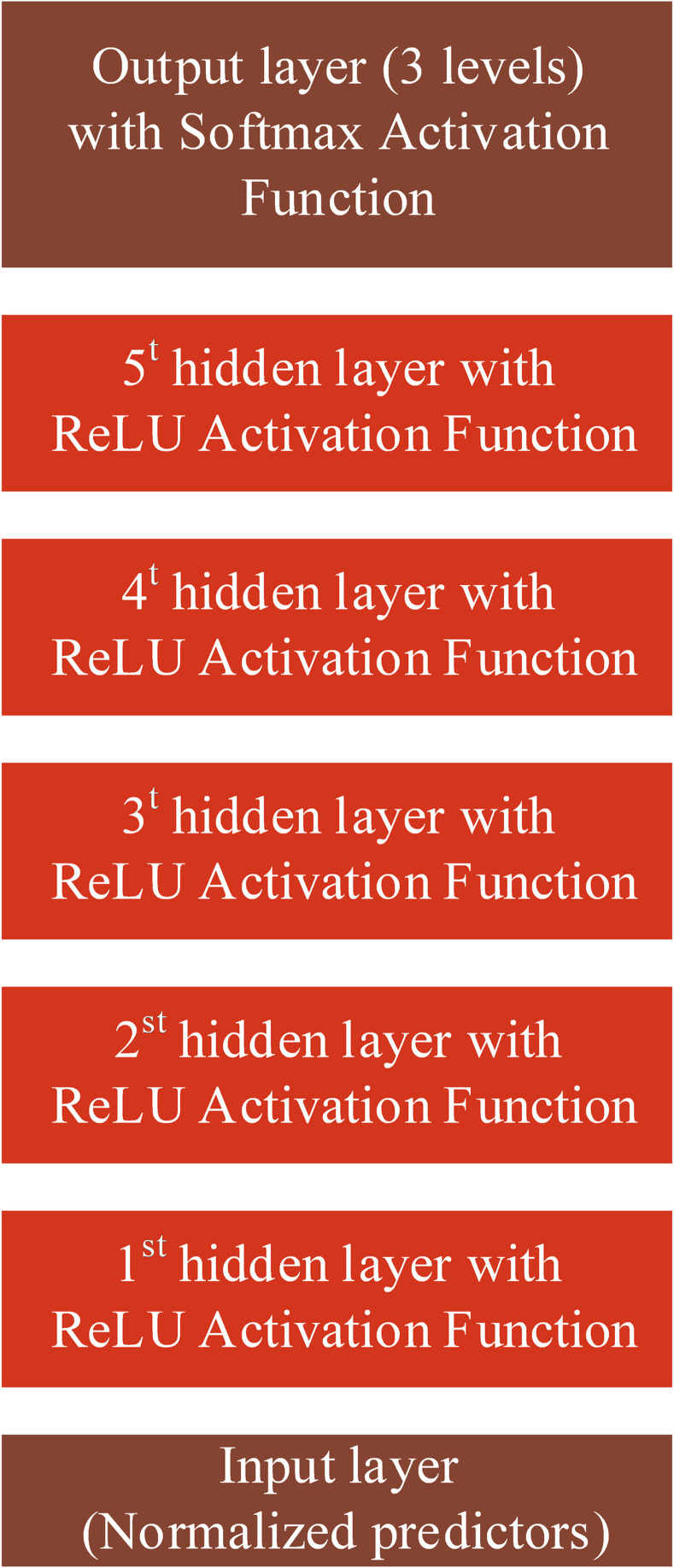
Network architecture of the proposed MLP model.

MLP neural network is a learning algorithm inspired by the brain learning system. The MLP consists of three kinds of layers, including the input layer, hidden layer, and output layer. In a neural network, the number of hidden layers and the number of neurons within each layer is crucial since the network’s architecture affects the number of parameters and, consequently the generalizability of the developed model. The MLP is a feed-forward neural network that maps input feature vectors into a set of proper outputs. The method uses the backpropagation learning algorithm in the training phase to estimate the networks’ weights. The previous studies on crash severity analysis have mostly employed shallow neural networks with only one hidden layer [64–66]. Nonetheless, the flexibility of deep neural networks with multiple hidden layers is superior in discovering the latent effects of input variables on crash severity due to their complex mechanism. Therefore, after tuning the hyperparameters (number of hidden layers and nodes within each hidden layer) via a grid search, this study applied a deep MLP neural network with five hidden layers, each containing fifty neurons. The architecture of the MLP model is illustrated by Fig 1.

### 2.8 Performance metrics

Regarding the class imbalance issue in crash severity datasets, one should be cautious to evaluate the developed models objectively and correctly. The recent studies in the literature revealed that Area under the Curve (AUC), accuracy, F-measure, and precision are sensitive to outcome class distribution. By contrast, specificity and recall (sensitivity) are shown to be robust against class imbalance [67]. However, only a few studies on crash severity analysis focused on whether these performance metrics have appropriate values for the imbalanced data [66]. The overall accuracy, the most common performance metric in classification problems, is defined as the proportion of the test dataset which has been accurately predicted by the model. The accuracy performs poorly when dealing with highly imbalanced datasets. The geometric mean (G-mean) of the sensitivity values for all different multiple classes can be utilized as a robust performance metric to compare the predictive power of ML classifiers for the analysis of imbalanced datasets. The G-mean for multi-class classification is formulated in Eq (7):

$$G-mean={\left(Class\_1\;accuracy\;\times Class\_2\;accuracy\times \ldots \times \;Class\_K\;accuracy\right)}^{\frac{1}{K}}$$ (7)

where, *K* is the number of classes, and the *Class*_i *accuracy* corresponds to the sensitivity value of class i.

## 3. Results

After preprocessing the raw data, the crash severity prediction power of five ML models is assessed and compared. The models are evaluated using the stratified K-fold cross-validation technique. This method proposes a robust evaluation of the average predictive performance at the cost of training each model for K times. It divides the dataset into K equal partitions where the ratios of each target class are equal to the corresponding ratio in the full dataset. Each time, the model is trained on the K-1 folds, and the remaining part is employed for model validation. Finally, the performance metrics for all K models are averaged for each ML algorithm. The results of the stratified 10-fold cross-validation for the developed ML algorithms are summarized in Table 3.

**Table 3:** Classification performance with/without data treatment for truck driver crash injury severity analysis.

Model	Performance metric	SMOTE oversampling		Feature selection and SMOTE
		Yes	No	
**RF**	Overall accuracy (%)	92.6	78.4	89.3
	Class KA recall (%)	94.3	61.3	87.2
	Class BC recall (%)	93.0	34.4	89.5
	Class O recall (%)	90.3	99.2	91.2
	G-mean (%)	92.5	59.4	89.3
**KNN**	Overall accuracy (%)	90.0	64.1	87.1
	Class KA recall (%)	98.9	57.4	96.9
	Class BC recall (%)	98.2	34.7	95.4
	Class O recall (%)	73.0	76.6	69.2
	G-mean (%)	89.2	54.9	86.1
**MLP**	Overall accuracy (%)	85.5	71.2	80.5
	Class KA recall (%)	92.4	66.2	84.4
	Class BC recall (%)	84.1	37.1	78.9
	Class O recall (%)	80.0	84.8	78.3
	G-mean (%)	85.3	59.3	80.5
**GBDT**	Overall accuracy (%)	84.6	82.9	57.5
	Class KA recall (%)	91.3	69.9	91.3
	Class BC recall (%)	73.3	53.8	38.7
	Class O recall (%)	88.8	97.6	43.2
	G-mean (%)	84.1	71.6	53.5
**SVM**	Overall accuracy (%)	80.1	67.5	69.8
	Class KA recall (%)	88.5	68.5	84.2
	Class BC recall (%)	86.5	55.1	81.1
	Class O recall (%)	65.5	71.2	61.8
	G-mean (%)	79.4	64.5	75.0

Based on the results, the SMOTE oversampling method has efficiently improved the overall accuracy of the models by around 14%. Moreover, the feature reduction has decreased the overall accuracy up to only 5% in most of the models; including RF, KNN, and MLP. Whereas the accuracy of the SVM model falls by more than 5% as an aftermath of the RF feature selection, the performance measure still stands higher than that of the SVM without oversampling. The results indicate that the Gradient-Boosted Decision Trees (GBDT) algorithm outperforms other models for the original imbalanced dataset, having the highest overall accuracy and severe class KA recall values. Unlike all the other models, the GBDT is the only exception which does not make a forecast with a higher accuracy once both the SMOTE and feature selection are conducted. The model also misclassifies the less severe crashes (classes BC and O) to a relatively noticeable degree after the feature selection is applied to the dataset. This reverse imbalance has exacerbated the GBDT’s overall accuracy and G-mean values. However, the KA class recall value which is a more objective performance metric is larger for all the preprocessed ML models than the models with no preprocessing. The K-Nearest Neighbor (KNN) model with preprocessed data shows the highest average recall value for injury crashes (KA and BC classes) among all the competing algorithms, but in return, its G-mean value is ranked second due to a low recall value for the non-injury severity class (class O). The Random Forest (RF) algorithm with SMOTE oversampling and RF feature importance approach demonstrates the most promising results in the classification of truck-involved crash severity with a G-mean value of 89.3%. The worst class recall value of the RF algorithm without prior oversampling is only 34.4% in comparison to the corresponding value of 90.3% in the up-sampled model. More on the impacts of the preprocessing scheme on the quality of the final forecasts, the recall values of the high and low severity classes (KA and BC) increase when the SMOTE is carried out on the data, and see a decrease after the application of the RF feature selection. The corresponding value for the non-injury class label, nevertheless, has almost always been declined by both of the oversampling and feature selection processes.

**Fig 2:**
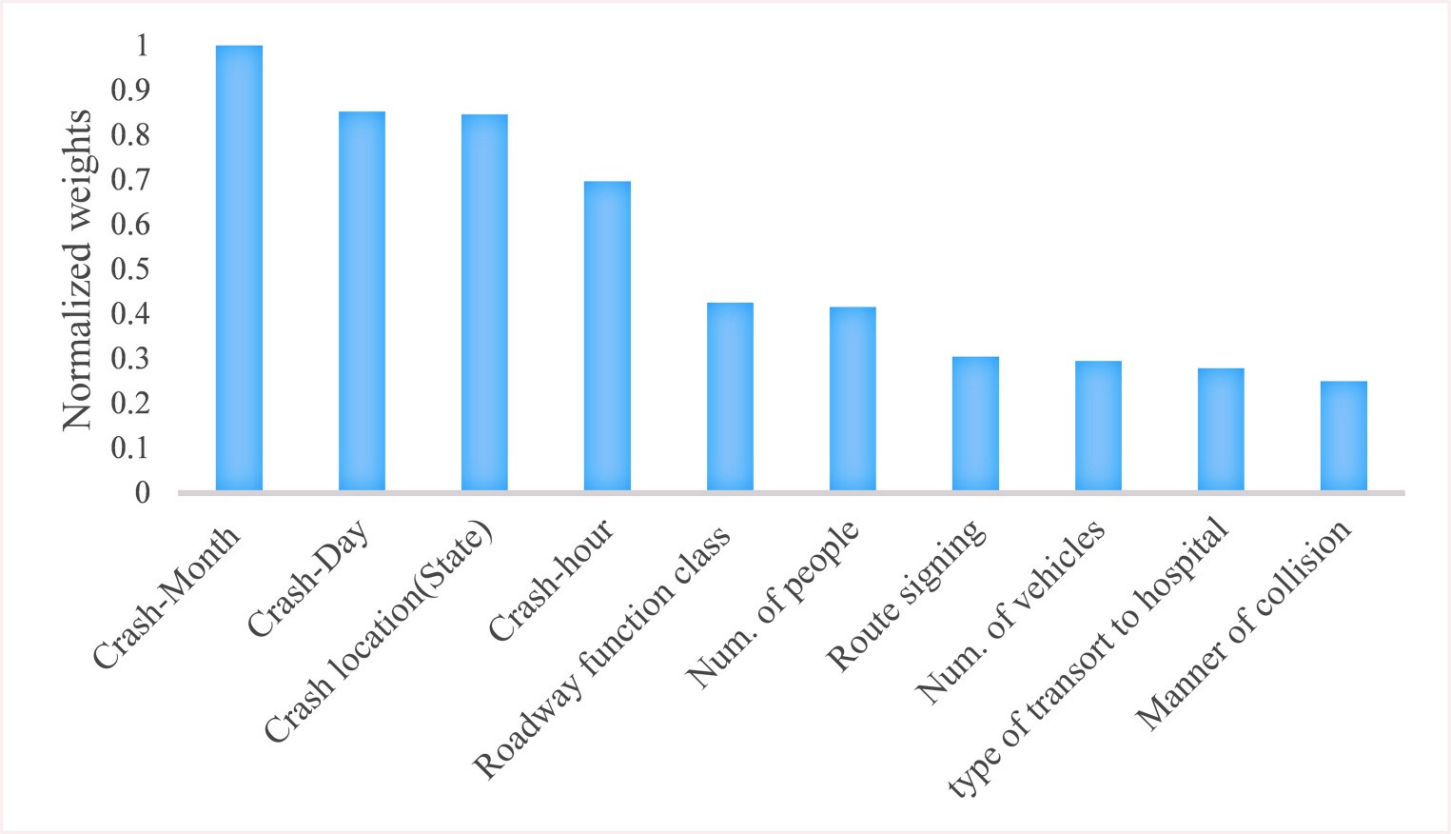
Random forest feature importance plot.

It should be noted that the feature importance analysis performed by the RF model indicated that the month, day, the hour of the crash, and location of crash occurrence (States), roadway function class, number of people involved, route signing, number of vehicles involved, type of transport to the hospital, and the manner of collision are the most important contributing factors to truck-involved crash severity (see Fig 2), while the roadway surface type, light and atmosphere conditions, the total number of lanes, vehicle and cargo body types, vehicle’s owner and vehicle’s registration state are poorly related to the injury severity levels of truck crashes. Finally, the feature importance analysis suggests that “the type of transport to hospital” is a significant EMS-related factor in injury severity of truck-involved crash events, but none of the considered environmental and vehicle-related attributes turns out to have a strong statistical association with the study target variable.

## 4. Discussion

It requires a thorough consideration to evaluate the classification performance of crash severity prediction models, and compare it to the literature. First, regarding the class imbalance issue, it is clear that the number of classes influences the results such that the multiclass classification models almost always indicate weaker results than binary classification models. Secondly, the research studies in the literature have examined datasets with different sizes, information richness, and variable counts, and it is obvious that more details add to the accuracy of the models. Thirdly, pretraining data treatment, especially the application of the class balancing methods, can largely affect the quality of the forecast on minority classes. To make the results comparable to previous studies, the class label attributes are aggregated into three categories, and the number of variables is reduced to a comparable value of 30. The prediction performance of the models from the literature which have three class labels is presented in Table 4. The table suggests that the estimated GBDT model in this paper is comparable with the best results in the literature using the imbalanced data. In addition, the overall accuracy of the proposed RF model with the preprocessed data is higher than that of most competing models in the literature, and in the only exceptional case, the difference is negligible. However, considering the imbalanced characteristics of the crash datasets, the G-mean would be a more reliable performance metric than the overall accuracy, and in this regard, the developed RF classifier has shown the best performance of all its ML counterparts from the literature.

**Table 4:** Comparison of crash severity prediction performance of proposed models with previous studies.

Study	Original crash data	Class balancing	ML Algorithm	G-mean	Overall accuracy
Jamal et al. [39]	(N = 13,546)	No	XGBoost	41.0	93.1
	22 Variables				
Zhou et al. [44]	(N = 28,605)	Yes	XGBoost	47.2	57.0
	16 Variables				
Nickkar et al. [23]	(N = 99,578)	No	Random Forest	60.9	88.6
	12 Variables				
Chen et al. [74]	(N = 23,433)	No	Bayesian Network (BN)	42.6	65.8
	22 Variables				
Gupta et al. [75]	(N = 5,402)	Yes	Random Forest	61.8	84.0
	20 Variables				
Chang and Chien [40]	(N = 1,620)	No	Classification and Regression Tree (CART)	57.7	67.7
	22 Variables				
Zheng et al. [76]	(N = 21,436)	Yes	Convolutional Neural Network (CNN)	22.5	90.2
	12 Variables				
Kunt et al. [65]	(N = 1,063)	No	MLP	76.6	77.4
	12 Variables				
Nguyen et al. [77]	(N = 42,102)	Yes	Decision Tree (C4.5)	84.9	90.2
	14 Variables				
**This paper**	(N = 3,643)	Yes	Random Forest	89.3	89.3
	30 Variables				
**This paper**	(N = 3,643)	No	GBDT	71.6	82.9
	110 Variables				

Considering the classification accuracy values in Table 3, it is worth noting that the GBDT algorithm outperforms its other counterparts significantly when applied directly to the imbalanced data while the RF algorithm is superior to the other ML alternatives after the SMOTE oversampling is utilized in the preprocessing stage. The GBDT is an ensemble learning approach based on boosting decision trees, and it produces a combination of sequential trees from weighted training data with a slow learning rate. At each step, a new decision tree is trained to fit the residuals of the ground truth and prediction [68]. This boosting design enables the GBDT model to focus more on difficult cases (crash minority classes) through generating an optimal set of trees, and therefore, is desirable to handle imbalanced data. In this way, the GBDT algorithm can not only classify the crash severity classes with small sizes (e.g., class KA), but also improve the overall performance metric [69]. There is also significant empirical evidence in the field of finance that GBDT performs better than other ML algorithms in the classification of imbalanced data [70, 71]. On the other hand, the efficiency and accuracy of the RF algorithm in crash severity classification is also repeatedly reported in the literature. A comprehensive literature review paper by Wang et al. [72], presented the state-of-the-art studies that compare different analytical methods applied in injury severity prediction of the road traffic crashes. This research analyzed 56 of such research papers published during 2001 to 2021. The review paper reported that RF was the algorithm with the best results, achieving the best performance in 70% of the times that it was used, and in 29% of all the inspected studies [73]. Hence, the existing methodological reasons and empirical evidence in the literature verify the obtained results in this research.

The RF feature selection is employed in this study, where 70% of variables are discarded, leading to a 5% decrease in the overall accuracy in most of the models. Indeed, eighty discarded variables explain only negligible variations in crash severity levels. Hence, the RF feature selection step prevents high computational costs without significantly increasing the classification error. These results are aligned with the previous findings. Wang et al. [72] applied a wrapper-based feature selection algorithm which eliminates 43% of the input features, and worsens in the crash severity classification performance of the SVM, RF, and C4.5 algorithms by an average of 5.7%. Moreover, other studies reported between 1.5% to 7.8% change in the overall accuracy of ML classifiers after implementing various feature selection algorithms to the traffic safety data [78, 79]. The results in Table 1 signify that the increase effect of the SMOTE on the overall accuracy and recall values of the data outweighs the decrease effect of the RF feature selection method, except for the SVM and GBDT models. It indicates that these ML classifiers are too sensitive to feature reduction when learning a crash severity prediction model on a high dimensional dataset while the other three algorithms perform flexibly in the feature selection step.

The DMI algorithm is used to impute the missing values. The simulation studies have confirmed the efficiency of this method when applied to different tabular datasets with various distributions of missing cells and missing ratios, where in all cases, the DMI reached an imputation accuracy of over 70% [50]. Accordingly, the results of this study would be more reliable than those of the former studies on truck-involved crashes, which have simply omitted the crash observations containing missing values [25, 43, 45]. Research has shown that the complete case analysis (CC) would flaw the sample, leading to erroneous inferences [12]. Also, the CC approach aggravates the efficiency of the data mining tools in crash severity classification by discarding valuable information in small datasets. That is especially the case when analyzing specific crash types with low relative frequencies.

The RF feature importance selection method identifies the influential factors on truck crash severity. The factors can be categorized into four groups: (1) Temporal factors, including crash month, day, and hour; (2) Spatial (Roadway) features, such as crash state (location), road function class, and route signing; (3) Crash characteristics, including number of people, and manner of collision; (4) Emergency medical service (EMS): type of transport to hospital. According to the information gain ratios for 110 available variables, the human (truck driver) factors, and environmental attributes have relatively weaker associations with the injury severity of crashes. Another way to interpret this issue would be that the effects of human and environmental factors are captured by the temporal and spatial factors and crash characteristics. For example, the impact of weather is covered by the crash month and state. The TIFA 2010 dataset provides information for both the EMS and vehicle-related factors. The factors pertaining to truck features were not recognized as significant, but the EMS was shown to have an influence on crash severity. Therefore, as the effects of the EMS factors cannot be extracted by other crash severity contributing factors, it is important to consider these variables when investigating influential factors on crash injury severity.

Previous studies on truck-involved crashes have mostly highlighted the importance of highway functional class on crash severity outcomes [23]. Ahmed et al. [32] reported that the odds of a severe crash increase to 2.3 and 4.5 times when a truck has a crash on state and interstate highways, respectively. This matches with the similar result of this study on the significance of the roadway function class. Regarding the influence of weather and lighting conditions on crash severity, the results in the literature are inconsistent. It should be noted that while some studies ranked the weather and lighting conditions as one of the main predictors of the crash severity outcome [19, 31, 75], others are in accordance with the findings of this paper, indicating the insignificance of these variables on crash severity [23, 35]. In addition, the significance of the temporal factors has been confirmed in the literature of subject as Zheng et al. [19] identified the importance of time of day and weekday in the severity levels of crashes involving trucks.

To the best of our knowledge, no study has investigated the effects of the type of transport to the medical service unit and EMS arrival/notification time on the truck-involved crash severity. This study proposes a comprehensive perspective of the impact of various human-related factors (driving while intoxicated, driver experience, driver’s previous penalties/crashes, speeding, etc.), as well as road-environment-related and vehicle-related factors of the crash severity outcomes of truck-involved crashes.

## 5. Conclusions

This paper aims to propose a multi-step preprocessing framework prior to model development to tackle missing, imbalanced, and high dimensional traffic safety data. A decision tree-based (DMI) algorithm is applied to impute the missing values. The SMOTE is implemented for data balancing treatment, and the random forest feature importance analysis is used for the purpose of dimensionality reduction. The feature importance analysis is also utilized to identify the most important predictors of the truck crash severity levels. To reveal the performance of the data preprocessing scheme, it is employed to the TIFA 2010 dataset which encompasses the contributing factors to the truck-involved crash severity regarding roadway, environment, vehicle, crash characteristics, and human (truck driver) as well as the emergency medical service. Several ML models, including the RF, KNN, MLP, SVM, and GBDT classification algorithms are developed and evaluated on both the balanced and imbalanced data using the G-mean, recall, and overall accuracy values measured by the 10-fold cross-validation. Finally, the influential factors on the truck crash severity are reported and interpreted.

Based on the results, of the five estimated ML algorithms, bagging applied to decision trees (e.g., random forest) combined with the SMOTE oversampling achieved the best crash severity classification performance. In addition, the random forest algorithm suggests an efficient feature reduction framework with negligible adverse effects on the classification accuracy, discarding a large amount of unnecessary computational expense. On the other hand, the Gradient Boosting Decision Tree (GBDT) model performed significantly better than its counterparts when applied to the imbalanced dataset. It is concluded that the month, day, and location of crash occurrence (crash state), the hour of the crash, roadway function class, number of people involved, route signing, number of vehicles involved, type of transport to the hospital, and the manner of collision are the most critical influential variables on truck-involved crash severity. These factors were categorized into four groups of temporal, spatial (roadway), crash characteristics, and emergency medical service (EMS). By contrast, the relative importance of roadway surface type, light and atmosphere conditions, total number of lanes, vehicle and cargo body types, vehicle owner, and vehicle registration state variables were identified to be low. The results bring further evidence supporting that the imbalanced crash severity data must be treated before the model estimation to prevent models from being biased toward the majority classes. Moreover, RF is a robust ML algorithm for crash severity prediction with a low computational cost and high accuracy on all the class labels when applied to preprocessed (balanced and dimensionally reduced) data. It also offers a useful dimensionality reduction tool as a by-product with its feature importance criterion. The developed models can be utilized by transport authorities and road safety policymakers to eliminate the socioeconomic burden of severe crashes, targeting the most influential variables. To sum up, the proposed comprehensive data preprocessing scheme is validated to deal with traffic safety datasets which have small sizes, and contain missing, imbalanced, and high dimensional data.

The feature importance analysis highlights the relative significance of the spatiotemporal variations on truck crash severity outcomes. The findings suggest that these spatiotemporal dependencies cause crashes in temporal or spatial proximity to have similar severity outcomes. Nonetheless, the current literature on applying ML techniques to crash severity analysis considers the crash observations as independent events, which might result in biased parameter estimates and inferences. Future research work should shed light on these spatiotemporal associations, adopting more advanced deep neural network models, such as Graph Convolutional Networks (GCN). A second aspect on which future studies can be based is to increase the interpretability of the ML models using different factor importance ranking methods such as SHAP (SHapley Additive exPlanation) value [9]. There also exists need for further research work on the improvement of causal inference in ML models which lets scientists and experts to explore more deeply the crash severity contributing factors, and the interaction effects among them, and thus, achieve a more profound understanding of the crash mechanism. This task can be fulfilled through novel methodologies such as the association rule mining, Bayesian networks, and unsupervised cluster analysis [6, 9, 80]. Finally, the promotion of the efficiency and reduction of the computational cost when fitting complicated ML algorithms to massive traffic safety data is highly important. Hence, Future research should focus more on online learning methods such as parallelized FP-Growth algorithm [5] which can handle large high dimensional datasets appropriately.

## Supporting information

S1 File The dataset used in this study. (ZIP)
