# Supplementary material for: Classification of truck-involved crash severity: Dealing with missing, imbalanced, and high dimensional safety data
Source: PLoS One. 2023 Mar 22;18(3):e0281901. doi: 10.1371/journal.pone.0281901 (PMC10032500; doi:10.1371/journal.pone.0281901)
Supplement: S1 File — (ZIP) [file pone.0281901.s001.zip › TIFA_Codebook2010.pdf]

UMTRI-2012-30 NOVEMBER 2012

Trucks Involved in Fatal Accidents Codebook 2010

Linda Jarossi
Daniel Hershberger
John Woodrooffe

Center for National Truck and Bus Statistics

UMTRI
University of Michigan
Transportation Research Institute

UMTRI–2012-30

TRUCKS INVOLVED IN FATAL ACCIDENTS CODEBOOK 2010
(Version October 22, 2012)

Linda Jarossi

Daniel Hershberger

John Woodrooffe

Center for National Truck and Bus Statistics

University of Michigan Transportation Research Institute

November 2012

The research reported herein was conducted under general research funds provided by the Federal Motor Carrier Safety Administration. The opinions, findings, and conclusions expressed in this publication are those of the authors and not necessarily those of the U.S. Department of Transportation or the Federal Motor Carrier Safety Administration.

Technical Report Documentation Page

1. Report No.	2. Government Accession No.	3. Recipient's Catalog No.
UMTRI-2012-30		

4. Title and Subtitle	5. Report Date
Trucks Involved in Fatal Accidents Codebook 2010 (Version October 22, 2012)	November 2012
	6. Performing Organization Code
7. Authors<br>Linda Jarossi, Daniel Hershberger, and John Woodrooffe	8. Performing Organization Report No.<br>UMTRI-201-30
9. Performing Organization Name and Address<br>University of Michigan<br>Transportation Research Institute<br>2901 Baxter Road<br>Ann Arbor, MI 48109–2150	10. Work Unit No.<br>065819<br>11. Contract or Grant No.<br>DTMC75–08–H–00005
12. Sponsoring Agency Name and Address<br>U.S. Department of Transportation<br>Federal Motor Carrier Safety Administration<br>400 Seventh Street, S.W.<br>Washington, D.C. 20590	13. Type of Report and Period Covered<br>Special Report<br>Task A<br>14. Sponsoring Agency Code

15. Supplementary Notes

16. Abstract

This report provides documentation for UMTRI's file of Trucks Involved in Fatal Accidents (TIFA), 2010, including distributions of the code values for each variable in the file. The 2010 TIFA file is a census of all medium and heavy trucks involved in a fatal accident in the United States. The TIFA database provides coverage of all medium and heavy trucks recorded in the Fatality Analysis Reporting System (FARS) file. TIFA combines vehicle, accident, and occupant records from FARS with information about the physical configuration and operating authority of the truck from the TIFA survey.

17. Key Words	18. Distribution Statement
Medium trucks, heavy trucks, fatal accident data	Unlimited

19. Security Classification (of this report)	20. Security Classification (of this page)	21. No. of Pages	22. Price
Unclassified	Unclassified	139	

Acknowledgments

The data documented in this report are the product of the dedicated efforts of many people. The project originated under the direction of Ken Campbell and Oliver Carsten. Daniel Blower directs the project currently. Robert Allen, Cherie Donzé, Ray Grabel, Bo Hansen, Deborah Keefer, Robert Korniski, Christine Schmidt, Lee Smart, and Lesia Syrowatka made the accuracy of the data a matter of personal pride. Susan Nieuwenhuis ably helped edit the data.

The project would not have been possible without the willing cooperation of thousands of truck owners, operators, and police officers across the country. We are very grateful for their assistance.

The TIFA survey is conducted by the Center for National Truck and Bus Statistics at the University of Michigan Transportation Research Institute with support from the Federal Motor Carrier Safety Administration. The National Highway Traffic Safety Administration's National Center for Statistics and Analysis and FARS analysts in the states provided assistance in acquiring police reports from the respective states. We are sincerely grateful for their efforts.

INTRODUCTION

Overview

This report documents the October 22, 2012 version of the Trucks Involved in Fatal Accidents (TIFA) 2010 dataset. The report summarizes all the information in the computerized data file. The 2010 TIFA file contains records for all the medium and heavy trucks that were involved in fatal traffic accidents in the 50 states and District of Columbia during calendar year 2010. Trucks with a gross vehicle weight rating of 10,000 pounds or less, primarily pickups, are excluded as nonsample, as are emergency vehicles and other non–trucks. All the vehicles described are from Version 3Feb12 of the Fatality Analysis Reporting System (FARS) file for 2010 accidents, developed by the National Highway Traffic Safety Administration (NHTSA).

The 2010 TIFA file is a census file, which means there is one record for each of the 3,699 medium and heavy trucks involved in a fatal accident in 2010. The codebook presents frequencies for the FARS variables and the TIFA survey variables for the actual number of cases in the file.

Missing data rates for TIFA survey variables are generally low. The range of missing data values is illustrated by the following examples. Cab style (variable 1003) could not be determined for 50 (1.4%) of the cases and vehicle configuration (variable 1048) could not be determined for 54 (1.5%) of the 3,699 cases. Gross vehicle weight rating (variable 1050) could not be determined for 63 (1.7%) cases and hours driving (variable 1062) is unknown for 1,400 (37.8%) cases.

While we have tried to make variable numbering consistent from year to year, we have no control over changes made to the FARS variables. Thus, variable numbers and code values in the 2010 TIFA file are not fully consistent with the files for 1980 through 2009. Accordingly, the 2010 codebook should not be used as a guide to the earlier datasets.

The dataset includes virtually all the variables from the public version of the FARS file: the crash variables, the vehicle variables (for the truck), and the occupant variables (for the driver of the truck). A few cases had no occupant record because the vehicle was not occupied at the time of the accident. These cases have been padded with the appropriate missing data codes. All variables are at the vehicle level; that is, there is one record for each truck involved. In addition to the variables from FARS (variables 1 through 363), there is a set of variables (numbers 1001 through 1126) that contains the information from the TIFA survey form. The bulk of this information is produced by telephone interviews with the driver, owner, or some other involved party. Some of this information is transcribed from police reports collected from the states.

While the FARS file includes much information on the crash environment and events, information on the vehicles involved, particularly trucks, is limited. Details about the physical configuration of the trucks involved are supplied by the TIFA survey. (The survey form is reproduced in the appendix of this codebook.) The TIFA survey collects a detailed physical description of the truck. These data include the cab style of the power unit; gross vehicle weight rating and gross combination weight rating; trailer type, cargo body style and cargo type; and the company type and operating authority of the operator of the vehicle. The combination of the

FARS accident level variables with the physical detail of the TIFA survey produces the most detailed account of fatal truck accidents available.

Sources of Information

The first step in the acquisition of the data to supplement FARS was to obtain, from the states, copies of the police reports on all fatal accidents involving at least one truck. While the format of these reports varies considerably from state to state, they all include the identities of the owner and the driver of the vehicles involved (though some states remove this information) and a description, sometimes very brief, of what occurred. These police reports were subsequently used in identifying the appropriate respondent to contact and in checking responses for accuracy.

Police reports could not be obtained from five states, representing 210 cases. Michigan and Mississippi were unable to send 2 reports, Nevada was missing only one.

The majority of missing police reports were from Pennsylvania; they supplied none for their 171 cases. Utah was unable to send any of their 31 police reports.

Information was collected primarily by telephone interviews. The person or company contacted was, when possible, the owner of the vehicle as listed in the police report. If no contact could be made with the owner, an attempt was made to reach the driver. If neither the owner nor the driver could be reached, as much information as possible was collected from other parties, such as the police officer who investigated the accident or the tow truck operator if the vehicle was towed from the scene. Finally, if no knowledgeable respondent could be found, as much information as possible was coded from the police report. For these cases, variable 1063, which documents whether an interview was conducted, is coded "no," and variable 1064, police report, is coded "yes."

Editors carefully checked each completed interview. All modifications to the survey responses received from an interview are indicated in variables 1066 through 1070. Imputations made by the editors to fill in missing data elements are also indicated there. The numbers coded in these variables are the question numbers on the interview form (see appendix). Thus a "17" in variable 1067 indicates that the second item corrected or derived for that particular case was the response to question 17 on the interview form. There is no particular pattern to the order in which such modifications are indicated. Derivations were made when the editor was able to deduce a piece of information to fill in something missing on the interview form. For example, the type of operating authority was sometimes deduced from the name of the vehicle’s owner and their business type.

Number of Cases

Version 3Feb12 of the 2012 FARS file lists 3,789 vehicles (excluding fire trucks) involved in fatal accidents in the United States, that were identified as possible medium or heavy trucks. However, some of the selected vehicles were subsequently found to be light, rather than medium or heavy, trucks, and twenty-two cases were buses. In particular, a number of vehicles coded by FARS as trucks turned out to be pickups, emergency vehicles, or other light vehicles. Also, vehicles that did not conform to the rules for inclusion in FARS were designated nonsample. These included vehicles parked off the roadway (e.g., on the shoulder) or legally parked at the side of the road, or cases in which the fatality occurred prior to the crash (e.g., heart attacks). Altogether 68 cases were determined to be nonsample, as defined, and 22 were

buses. Subtracting those 90 from the FARS file total of 3,789 cases leaves 3,699, which is the number of trucks involved in fatal accidents in 2010.

Format of Codebook

The main body of the codebook provides frequency distributions of all variables and code levels in the TIFA 2010 data file (Motor Vehicles In-Transport). For each variable, the codebook provides information about the variable, name, format, and type. “Variable” (such as V19) is the name of the variable in the SAS data file. “Name” (e.g. Route Signing) is the descriptive title corresponding to the FARS or TIFA variable. “Format” refers to the name of the SAS format that provides labels for specific code levels of the variable. “Type” describes the storage type of the variable. Data are stored either as Numeric or Char (character) data. “Length” is the SAS storage length in bytes for the variable.

Obtaining Information from the Dataset

This report provides counts and distributions of the code values for each variable in the file. These tabulations are useful for understanding the variables available in the file, the completeness of the data, and the number of cases with any specific code value.

Many research questions require more detailed cross-classification of the data. In general, different types of trucks are used differently. In comparing the accident experience of straight trucks with that of tractor–semitrailers, for example, one might wish to examine the distributions of trip type and carrier type. While this dataset is not made available to the public by UMTRI, the staff of the Center for National Truck and Bus Statistics at UMTRI will be pleased to make the appropriate runs for outside users. Requests for consultation on and analysis of the data are welcome and may be addressed to Daniel Blower at (734) 764–0248. Finally, while every effort has been made to check the accuracy of the data, the file may contain errors not yet detected.

TRUCKS INVOLVED IN FATAL ACCIDENTS, 2010
FARS CRASH VARIABLES

Variable Number	Variable Name	Page Number
v1	CASE STATE	1
v2	CASE NUMBER	2
v7	CITY	2
v8	COUNTY	3
v9	CRASH DATE - MONTH	3
v10	CRASH DATE - DAY	4
v11	CRASH DATE - YEAR	4
v12	CRASH TIME - HOUR	4
v13	CRASH TIME - MINUTE	5
v14	NUMBER OF VEHICLE FORMS - MVIT ONLY	5
v14B	VEHICLE FORMS SUBMITTED - ALL	6
v15	NUMBER OF PERSON FORMS	6
v16	LAND USE	7
v17	NATIONAL HIGHWAY SYSTEM	7
v18	ROADWAY FUNCTION CLASS	8
v19	ROUTE SIGNING	8
v20	TRAFFICWAY IDENTIFIER #1	9
v20_2	TRAFFICWAY IDENTIFIER #2	9
v21	MILEPOINT	9
v22	SPECIAL JURISDICTION	9
v23	FIRST HARMFUL EVENT	10
v24	MANNER OF COLLISION	13
v25A	RELATION TO JUNCTION WITHIN INTERCHANGE AREA	13
v25B	RELATION TO JUNCTION - JUNCTION	13
v26	RELATION TO ROADWAY	14
v27	TRAFFICWAY DESCRIPTION	14
v28	TOTAL LANES IN ROADWAY	15
v29	SPEED LIMIT	15
v30	ROADWAY ALIGNMENT	16
v31	ROADWAY GRADE	16
v32	ROADWAY SURFACE TYPE	17
v33	ROADWAY SURFACE CONDITION	17
v37	LIGHT CONDITION	17
v38	ATMOSPHERIC CONDITIONS	18
v39	WORK ZONE	18
v40	EMS NOTIFIED - HOUR	19
v41	EMS NOTIFIED - MINUTE	19
v42	EMS ARRIVAL - HOUR	19
v43	EMS ARRIVAL - MINUTE	20
v46	SCHOOL BUS RELATED	20
v48	RAIL GRADE CROSSING ID	20
v49	NUMBER FATALITIES IN CRASH	21
v50	DAY OF WEEK	21
v51	NUMBER DRINKING DRIVERS	21
v52	CRASH DATE - JULIAN	22
v53	NUMBER UNINJURED IN CRASH	22
v54	NUMBER C-INJURED IN CRASH	22
v55	NUMBER B-INJURED IN CRASH	23
v56	NUMBER A-INJURED IN CRASH	23
v57	NUMBER K-INJURED IN CRASH	23
v58	NUM UNK INJURED IN CRASH	24
v59	CRASH RELATED FACTORS #1	24

TRUCKS INVOLVED IN FATAL ACCIDENTS, 2010
FARS CRASH VARIABLES

Variable Number	Variable Name	Page Number
v60	CRASH RELATED FACTORS #2	25
v61	CRASH RELATED FACTORS #3	26
v62	ATMOSPHERIC CONDITIONS #1	26
v63	ATMOSPHERIC CONDITIONS #2	26
v71	LATITUDE	27
v72	LONGITUDE	27
v80	TYPE OF INTERSECTION	27

TRUCKS INVOLVED IN FATAL ACCIDENTS, 2010
FARS VEHICLE VARIABLES

Variable Number	Variable Name	Page Number
v104	VEHICLE NUMBER	29
v105	UNIT TYPE	29
v107	NUMBER OF OCCUPANTS	29
v108	VEHICLE MAKE	30
v109	VEHICLE MAKE-MODEL	30
v110	BODY TYPE	33
v111	MODEL YEAR	34
v112	VIN	35
v123	REGISTRATION STATE	35
v124	REGISTERED VEHICLE OWNER	37
v125	ROLLOVER	37
v125B	LOCATION OF ROLLOVER	37
v126	JACKKNIFE	38
v127	TRAVEL SPEED	38
v129	VEHICLE TRAILERING	38
v130	VEHICLE CONFIGURATION	39
v132	CARGO BODY TYPE	39
v133	SPECIAL USE	40
v134	EMERGENCY USE	40
v135	AREAS OF IMPACT - INITIAL DAMAGE	40
v136	AREAS OF IMPACT - MOST DAMAGE	41
v137	EXTENT OF DAMAGE	42
v139	VEHICLE REMOVAL	42
v140	FIRE OCCURRENCE	43
v144	MOST HARMFUL EVENT	43
v145	NUMBER OF DEATHS IN VEH	45
v151	VIN TRUCK FUEL CODE	46
v152	VIN TRUCK WEIGHT CODE	46
v153	VIN TRUCK SERIES	46
v155	LENGTH OF VIN	47
v156	NUMBER UNINJURED IN VEH	47
v157	NUMBER C-INJURED IN VEH	47
v158	NUMBER B-INJURED IN VEH	47
v159	NUMBER A-INJURED IN VEH	48
v160	NUMBER K-INJURED IN VEH	48
v161	NUM UNK INJURED IN VEH	48
v164	UNDERRIDE/OVERRIDE	49
v165	TRAFFIC CONTROL DEVICE	49
v166	DEVICE FUNCTIONING	50
v167_1	VEHICLE RELATED FACTORS #1	50
v167_2	VEHICLE RELATED FACTORS #2	51
v170	MOTOR CARRIER ID	51
v171	BUS USE	51
v172	GVWR	52
v179	HAZARDOUS MATERIAL CLASS NUMBER	52
v180	HAZARDOUS MATERIAL ID NUMBER	53
v181	HAZARDOUS MATERIAL INVOLVEMENT	53
v182	HAZARDOUS MATERIAL PLACARD	53
v183	HAZARDOUS MATERIAL RELEASED	53
v184	MCID ISSUING AUTHORITY	54
v185	MCID IDENTIFICATION NUMBER	54
v186	HIT AND RUN	54

Variable Number	Variable Name	Page Number
v190	PRE-EVENT MOVEMENT (PRIOR TO RECOGNITION OF CRITICAL EVENT	55
v191	CRITICAL EVENT - PRECRASH (EVENT)	55
v192	ATTEMPTED AVOIDANCE MANEUVER	57
v193	PRE-IMPACT STABILITY	57
v194	PRE-IMPACT LOCATION	58
v195	CRASH TYPE	58

TRUCKS INVOLVED IN FATAL ACCIDENTS, 2010
FARS DRIVER VARIABLES

Variable Number	Variable Name	Page Number
v207	DRIVER PRESENCE	61
v208	DRIVER DRINKING	61
v209	DRIVER'S LICENSE STATE	61
v210A	NON-CDL LICENSE TYPE	63
v210B	NON-CDL LICENSE STATUS	63
v211	LICENSE CLASS COMPLIANCE	63
v212	CDL LICENSE STATUS	64
v213	COMPLIANCE WITH CDL ENDORSEMENTS	64
v214	LICENSE RESTRICTIONS MET	65
v216	NUMBER OF PREV CRASHES	65
v217	NUMBER PREV SUSPENSIONS	65
v218	NUMBER OF PREV DWI CONV	66
v219	NUM PREV SPEEDING CONV	66
v220	NUM PREV OTHER MV CONV	66
v221	LAST CRASH - MONTH	67
v222	LAST CRASH - YEAR	67
v223	FIRST CRASH - MONTH	68
v224	FIRST CRASH - YEAR	68
v227	DRIVER RELATED FACTORS #1	69
v228	DRIVER RELATED FACTORS #2	71
v229	DRIVER RELATED FACTORS #3	72
v230	DRIVER RELATED FACTORS #4	73
v235	DRIVER HEIGHT (INCHES)	73
v236	DRIVER WEIGHT	73
v240	SPEED RELATED	74

TRUCKS INVOLVED IN FATAL ACCIDENTS, 2010

FARS DRIVER VARIABLES

TRUCKS INVOLVED IN FATAL ACCIDENTS, 2010
FARS OCCUPANT VARIABLES

Variable Number	Variable Name	Page Number
v306	OCCUPANT NUMBER	75
v308	OCCUPANT AGE	75
v309	OCCUPANT SEX	77
v310	OCCUPANT TYPE	77
v311	OCC SEATING POSITION	77
v312	RESTRAINT SYSTEM/HELMET USE	78
v313	AIR BAG AVAIL/FUNCTION	78
v314	OCCUPANT EJECTION	78
v315	EJECTION PATH	79
v316	OCCUPANT EXTRICATION	79
v317	ANY INDICATION OF MIS-USE OF RESTRAINT SYSTEM/HELMET USE	79
v320	DRUG STATUS	80
v321	DRUG INVOLVEMENT	80
v322	DRUG DETERMINATION	80
v323	DRUG TEST TYPE #1	80
v324	DRUG TEST RESULTS #1	81
v325	DRUG TEST TYPE #2	82
v326	DRUG TEST RESULTS #2	82
v327	DRUG TEST TYPE #3	83
v328	DRUG TEST RESULTS #3	83
v329	OCCUPANT INJURY SEVERITY	84
v330	TRANSPORTED TO MEDICAL FACILITY BY	85
v331	OCC DEATH DATE - MONTH	85
v332	OCC DEATH DATE - DAY	86
v333	OCC DEATH DATE - YEAR	86
v334	OCC DEATH TIME - HOURS	86
v335	OCC DEATH TIME - MINUTES	87
v336	LAG TIME CRASH/DEATH - HRS	87
v342	OCC FATAL INJURY AT WORK	88
v345	OCC ALCOHOL INVOLVEMENT	88
v346	OCC METH ALC DETERMINAT	88
v347	ALCOHOL TEST	89
v348	OCC ALCOHOL TEST RESULT	89
v349	ALCOHOL TEST STATUS	89
v361	RACE	90
v362	HISPANIC ORIGIN	90
v363	OCC DEAD ON ARRIVAL	91

TRUCKS INVOLVED IN FATAL ACCIDENTS, 2010
FARS OCCUPANT VARIABLES

TRUCKS INVOLVED IN FATAL ACCIDENTS, 2010
SURVEY VARIABLES

Variable Number	Variable Name	Page Number
v1001	POWER UNIT MAKE	93
v1002	POWER UNIT YEAR	93
v1003	CAB STYLE	95
v1006	TRUCK MODEL	95
v1007	POWER UNIT TYPE	95
v1008	STRT TRUCK BODY STYLE	95
v1009	STRT TRUCK OTHER BODY	96
v1010	POWER UNIT NO. OF AXLES	96
v1015	POWER UNIT CARGO	97
v1017	1ST TRAILER TYPE	97
v1018	1ST TRAILER NO. OF AXLES	98
v1023	1ST TRAILER BODY	98
v1024	1ST TRAILER OTHER BODY	99
v1025	1ST TRAILER CARGO	99
v1027	2ND TRAILER TYPE	100
v1028	2ND TRAILER NO. OF AXLES	100
v1033	2ND TRAILER BODY	101
v1034	2ND TRAILER OTHER BODY	101
v1035	2ND TRAILER CARGO	101
v1037	3RD TRAILER TYPE	102
v1038	3RD TRAILER NO. OF AXLES	102
v1043	3RD TRAILER BODY	102
v1044	3RD TRAILER OTHER BODY	103
v1045	3RD TRAILER CARGO	103
v1047	VEHICLE CONFIGURATION	103
v1048	VEHICLE COMBINATION CODE	104
v1049	NO. OF TRAILERS	106
v1050	GROSS VEHICLE WEIGHT RATING	106
v1050C	GROSS COMBINATION WEIGHT RATING	107
v1055	SPECIFIC CARGO	107
v1056	CARGO SPILLAGE	107
v1057	AREA OF OPERATION	107
v1058	OPERATING AUTHORITY	108
v1059	ACCIDENT TYPE	108
v1061	TRIP TYPE	108
v1062	HOURS DRIVING	109
v1063	INTERVIEW CONDUCTED	109
v1064	POLICE REPORT	109
v1065	FAX/MAIL	110
v1066	1ST QUESTION DERIVED	110
v1067	2ND QUESTION DERIVED	110
v1069	4TH QUESTION DERIVED	111
v1070	5TH QUESTION DERIVED	111
v1091	HAZARDOUS MATERIALS PLACARD	111
v1092	HAZARDOUS MATERIALS CLASS	111
v1093	HAZARDOUS PLACARD 4-DIGIT NUMBER	112
v1101	HEADWAY DETECTION/FORWARD CRASH WARNING	112
v1102	SIDE/OBJECT DETECTION	112
v1103	LANE DEPARTURE WARNING	112
v1104	ROLLOVER WARNING	113
v1105	ELECTRONIC STABILITY CONTROL	113
v1106	POWER UNIT TRACKING	113

Variable Number	Variable Name	Page Number
v1107	TRAILER TRACKING	113
v1108	SPEED LIMITER	113
v1109	AUTOMATIC COLLISION AVOIDANCE BRAKING	114
v1111	DRIVER COMPENSATION	114
v1112	DRIVER COMPENSATION OTHER	114
v1120	BUSINESS TYPE	116
v1121	SPEED LIMITER SETTING	118
v1125	YEARS OF TRUCK DRIVING EXPERIENCE	119
v1126	YEARS OF DRIVING EXPERIENCE FOR COMPANY	120

The CRASH Variables

Variables 1 through 80 are the FARS variables that describe the crash.

Variable	Name	Format	Type	Length
v1	CASE STATE	FSTATES	Numeric	4

N	Prcnt	Code	Label
110	3.0	1	Alabama
6	0.2	2	Alaska
53	1.4	4	Arizona
80	2.2	5	Arkansas
239	6.5	6	California
56	1.5	8	Colorado
23	0.6	9	Connecticut
9	0.2	10	Delaware
4	0.1	11	District of Columbia
194	5.2	12	Florida
143	3.9	13	Georgia
5	0.1	15	Hawaii
17	0.5	16	Idaho
122	3.3	17	Illinois
115	3.1	18	Indiana
90	2.4	19	Iowa
73	2.0	20	Kansas
95	2.6	21	Kentucky
101	2.7	22	Louisiana
14	0.4	23	Maine
35	0.9	24	Maryland
13	0.4	25	Massachusetts
86	2.3	26	Michigan
77	2.1	27	Minnesota
61	1.6	28	Mississippi
88	2.4	29	Missouri
17	0.5	30	Montana
50	1.4	31	Nebraska
20	0.5	32	Nevada
7	0.2	33	New Hampshire
64	1.7	34	New Jersey
44	1.2	35	New Mexico
121	3.3	36	New York

N	Prcnt	Code	Label
109	2.9	37	North Carolina
19	0.5	38	North Dakota
132	3.6	39	Ohio
90	2.4	40	Oklahoma
51	1.4	41	Oregon
171	4.6	42	Pennsylvania
2	0.1	44	Rhode Island
72	1.9	45	South Carolina
19	0.5	46	South Dakota
92	2.5	47	Tennessee
411	11.1	48	Texas
31	0.8	49	Utah
11	0.3	50	Vermont
94	2.5	51	Virginia
37	1.0	53	Washington
44	1.2	54	West Virginia
55	1.5	55	Wisconsin
27	0.7	56	Wyoming

Variable	Name	Format	Type	Length
v2	CASE NUMBER	4	Numeric	4

CASE NUMBER ASSIGNED WITHIN STATES

N	Prcnt	Code	Label
4	0.1	1	
		-	Case number
1	0.0	2779	

Variable	Name	Format	Type	Length
v7	CITY	6	Numeric	8

CITY – GSA GEOGRAPHIC LOCATION CODE

N	Prcnt	Code	Label
2,275	61.5	0	Not applicable

N	Prcnt	Code	Label
1	0.0	5	
		-	GSA code
1	0.0	9898	
13	0.4	9997	Other
2	0.1	9999	Unknown

Variable	Name	Format	Type	Length
v8	COUNTY	6	Numeric	8

COUNTY – GSA GEOGRAPHIC LOCATION CODE

N	Prcnt	Code	Label
72	1.9	1	
		-	GSA code
1	0.0	810	

Variable	Name	Format	Type	Length
v9	CRASH DATE - MONTH	MONTH10F	Numeric	8

N	Prcnt	Code	Label
264	7.1	1	January
249	6.7	2	February
287	7.8	3	March
298	8.1	4	April
290	7.8	5	May
305	8.2	6	June
307	8.3	7	July
372	10.1	8	August
341	9.2	9	September
330	8.9	10	October
351	9.5	11	November
305	8.2	12	December

Variable	Name	Format	Type	Length
v10	CRASH DATE - DAY	DAY10F	Numeric	8

N	Prcnt	Code	Label
115	3.1	1	
		-	Day of month
43	1.2	31	

Variable	Name	Format	Type	Length
v11	CRASH DATE - YEAR	11	Numeric	8

N	Prcnt	Code	Label
3,699	100.0	2010	2010

Variable	Name	Format	Type	Length
v12	CRASH TIME - HOUR	FACHOUR	Numeric	8

N	Prcnt	Code	Label
83	2.2	0	12:00 am - 12:59 am
82	2.2	1	1:00 am - 1:59 am
115	3.1	2	2:00 am - 2:59 am
96	2.6	3	3:00 am - 3:59 am
109	2.9	4	4:00 am - 4:59 am
148	4.0	5	5:00 am - 5:59 am
172	4.6	6	6:00 am - 6:59 am
178	4.8	7	7:00 am - 7:59 am
180	4.9	8	8:00 am - 8:59 am
204	5.5	9	9:00 am - 9:59 am
191	5.2	10	10:00 am - 10:59 am
228	6.2	11	11:00 am - 11:59 am
216	5.8	12	12:00 pm - 12:59 pm
232	6.3	13	1:00 pm - 1:59 pm
249	6.7	14	2:00 pm - 2:59 pm
206	5.6	15	3:00 pm - 3:59 pm
205	5.5	16	4:00 pm - 4:59 pm
165	4.5	17	5:00 pm - 5:59 pm
141	3.8	18	6:00 pm - 6:59 pm
126	3.4	19	7:00 pm - 7:59 pm

N	Prcnt	Code	Label
113	3.1	20	8:00 pm - 8:59 pm
81	2.2	21	9:00 pm - 9:59 pm
78	2.1	22	10:00 pm - 10:59 pm
97	2.6	23	11:00 pm - 11:59 pm
4	0.1	99	Unknown

Variable	Name	Format	Type	Length
v13	CRASH TIME - MINUTE	MINUT10F	Numeric	8

N	Prcnt	Code	Label
185	5.0	0	
		-	Minute
48	1.3	59	
4	0.1	99	Unknown Minutes

Variable	Name	Format	Type	Length
v14	NUMBER OF VEHICLE FORMS - MVIT ONLY	FVEHFORM	Numeric	8

NUMBER OF MOTOR VEHICLES IN-TRANSIT INVOLVED IN CRASH

N	Prcnt	Code	Label
693	18.7	1	1 vehicle
2,355	63.7	2	2 vehicles
428	11.6	3	3 vehicles
119	3.2	4	4 vehicles
40	1.1	5	5 vehicles
14	0.4	6	6 vehicles
15	0.4	7	7 vehicles
10	0.3	8	8 vehicles
3	0.1	9	9 vehicles
13	0.4	11	11 vehicles
2	0.1	12	12 vehicles
2	0.1	14	14 vehicles
5	0.1	15	15 vehicles

Variable	Name	Format	Type	Length
v14B	VEHICLE FORMS SUBMITTED - ALL	FVEHFORM	Numeric	8

N	Prcnt	Code	Label
648	17.5	1	1 vehicle
2,366	64.0	2	2 vehicles
446	12.1	3	3 vehicles
128	3.5	4	4 vehicles
46	1.2	5	5 vehicles
15	0.4	6	6 vehicles
15	0.4	7	7 vehicles
10	0.3	8	8 vehicles
3	0.1	9	9 vehicles
13	0.4	11	11 vehicles
2	0.1	12	12 vehicles
2	0.1	14	14 vehicles
5	0.1	15	15 vehicles

Variable	Name	Format	Type	Length
v15	NUMBER OF PERSON FORMS	FNUMPERS	Numeric	8

NUMBER OF PERSONS INVOLVED IN CRASH

Does not include uninjured bus or railway train occupants.

N	Prcnt	Code	Label
4	0.1	0	0
612	16.5	1	1 person
1,476	39.9	2	2 persons
811	21.9	3	3 persons
325	8.8	4	4 persons
200	5.4	5	5 persons
108	2.9	6	6 persons
53	1.4	7	7 persons
26	0.7	8	8 persons
16	0.4	9	9 persons
10	0.3	10	10 persons
11	0.3	11	11 persons
1	0.0	12	12 persons
18	0.5	13	13 persons

N	Prcnt	Code	Label
6	0.2	14	14 persons
11	0.3	15	15 persons
5	0.1	17	17 persons
1	0.0	19	19 persons
1	0.0	21	21 persons
1	0.0	23	23 persons
2	0.1	24	24 persons
1	0.0	56	56 persons

Variable	Name	Format	Type	Length
v16	LAND USE	V16_F	Numeric	3

LAND USE – FHWA CLASSIFICATION

Recode of Roadway Function Class (v18)

N	Prcnt	Code	Label
1,256	34.0	1	Urban area
2,421	65.5	2	Rural area
22	0.6	9	Unknown

Variable	Name	Format	Type	Length
v17	NATIONAL HIGHWAY SYSTEM	NHS10F	Numeric	8

National Highway System (NHS) includes the entire Interstate System, and consists of principal arterial system routes and some Strategic Highway Network connectors functionally classified below principal arterial.

N	Prcnt	Code	Label
1,702	46.0	0	This section IS NOT on the NHS
1,978	53.5	1	This section IS ON the NHS
19	0.5	9	Unknown if this section is on the NHS

Variable	Name	Format	Type	Length
v18	ROADWAY FUNCTION CLASS	RFUN10F	Numeric	8

N	Prcnt	Code	Label
			Rural
536	14.5	1	Principal Arterial-Interstate
820	22.2	2	Principal Arterial-Other
462	12.5	3	Minor Arterial
386	10.4	4	Major Collector
72	1.9	5	Minor Collector
141	3.8	6	Local Road or Street
4	0.1	9	Unknown Rural
			Urban
369	10.0	11	Principal Arterial-Interstate
140	3.8	12	Principal Arterial-Other Freeways or Expressways
392	10.6	13	Other Principal Arterial
175	4.7	14	Minor Arterial
55	1.5	15	Collector
125	3.4	16	Local Road or Street
22	0.6	99	Unknown

Variable	Name	Format	Type	Length
v19	ROUTE SIGNING	RTSGN10F	Numeric	8

N	Prcnt	Code	Label
905	24.5	1	Interstate
982	26.5	2	U.S. Highway
1,109	30.0	3	State Highway
315	8.5	4	County Road
			Local street
71	1.9	5	Township
217	5.9	6	Municipality
8	0.2	7	Frontage Road

N	Prcnt	Code	Label
82	2.2	8	Other
10	0.3	9	Unknown

Variable	Name	Format	Type	Length
v20	TRAFFICWAY IDENTIFIER #1	$20	Char	20

N	Prcnt	Code	Label
1	0.0	10	
		-	Roadway identifier
1	0.0	ZETTWELL RD	

Variable	Name	Format	Type	Length
v20_2	TRAFFICWAY IDENTIFIER #2	$20	Char	20

N	Prcnt	Code	Label
1	0.0	0022	
		-	Roadway identifier
1	0.0	ZUMBEHLRD	

Variable	Name	Format	Type	Length
v21	MILEPOINT	MILPT10F	Numeric	8

N	Prcnt	Code	Label
625	16.9	0	None
12	0.3	1	
		-	Actual to nearest .1 mile
1	0.0	34850	
203	5.5	99998	Not Reported
230	6.2	99999	Unknown

Variable	Name	Format	Type	Length
v22	SPECIAL JURISDICTION	SPJUR10F	Numeric	8

N	Prcnt	Code	Label
3,690	99.8	0	No Special Jurisdiction

N	Prcnt	Code	Label
1	0.0	1	National Park Service
7	0.2	3	Indian Reservation
1	0.0	9	Unknown

Variable	Name	Format	Type	Length
v23	FIRST HARMFUL EVENT	FHE10F	Numeric	8

FIRST HARMFUL EVENT CAUSING INJURY/PROPERTY DAMAGE

N	Prcnt	Code	Label
136	3.7	1	Rollover/overturn
1	0.0	2	Fire/explosion
0	0.0	3	Immersion
0	0.0	4	Gas inhalation
12	0.3	5	Fell/jumped from vehicle
0	0.0	6	Injured in vehicle (non-collision)
1	0.0	7	Other non-collision
244	6.6	8	Pedestrian
62	1.7	9	Pedalcyclist
16	0.4	10	Railway vehicle
9	0.2	11	Live animal
2,798	75.6	12	Motor vehicle in-transport
31	0.8	14	Parked motor vehicle
11	0.3	15	Non-motorist on personal conveyance
1	0.0	16	Thrown or falling object
0	0.0	17	Boulder
12	0.3	18	Other object (not fixed)
0	0.0	19	Building
3	0.1	20	Impact attenuator/crash cushion
5	0.1	21	Bridge pier or support
4	0.1	23	Bridge rail (includes parapet)
88	2.4	24	Guardrail face
23	0.6	25	Concrete traffic barrier
2	0.1	26	Other traffic barrier
5	0.1	30	Utility pole/light support
13	0.4	31	Other post, other pole or other supports
14	0.4	32	Culvert
12	0.3	33	Curb
22	0.6	34	Ditch

N	Prcnt	Code	Label
16	0.4	35	Embankment
8	0.2	38	Fence
7	0.2	39	Wall
1	0.0	40	Fire Hydrant
1	0.0	41	Shrubbery
48	1.3	42	Tree (standing only)
3	0.1	43	Other fixed object
1	0.0	44	Pavement surface irregularity (ruts, potholes, grates, etc.)
6	0.2	45	Working motor vehicle
1	0.0	46	Traffic signal support
0	0.0	48	Snow bank
0	0.0	49	Ridden animal or animal drawn conveyance
7	0.2	50	Bridge overhead structure
8	0.2	51	Jackknife (harmful to this vehicle)
10	0.3	52	Guardrail end
10	0.3	53	Mail box
19	0.5	54	Motor vehicle in-transport strikes or is struck by cargo, persons or objects set-in-motion from/by another motor vehicle in transport
0	0.0	55	Motor vehicle in motion outside the trafficway
9	0.2	57	Cable barrier
3	0.1	58	Ground
11	0.3	59	Traffic sign support
5	0.1	72	Cargo/equipment loss or shift (harmful to this vehicle)
0	0.0	98	Not reported
0	0.0	99	Unknown

The following list shows the code values given above grouped by collision type.

Non-collision Harmful Events
- 01 = Rollover/overturn
- 02 = Fire/explosion
- 03 = Immersion
- 04 = Gas inhalation
- 05 = Fell/jumped from vehicle
- 06 = Injured in vehicle (non-collision)
- 07 = Other non-collision
- 16 = Thrown or falling object
- 44 = Pavement surface irregularity (ruts, potholes, grates, etc.)

51 = Jackknife (harmful to this vehicle)
72 = Cargo/equipment loss or shift (harmful to this vehicle)

Collision with Motor Vehicle
12 = Motor vehicle in–transport
54 = Motor vehicle in–transport strikes or is struck by cargo, persons, or objects set–in–motion from/by another motor vehicle in–transport
55 = Motor vehicle in motion outside the trafficway

Collision with Object Not Fixed
08 = Pedestrian
09 = Pedalcyclist
10 = Railway vehicle
11 = Live animal
14 = Parked motor vehicle
15 = Nonmotorist on personal conveyance
18 = Other object (not fixed)
45 = Working motor vehicle
49 = Ridden animal or animal–drawn conveyance

Collision with Fixed Object
17 = Boulder
19 = Building
20 = Impact attenuator/crash cushion
21 = Bridge pier or support
23 = Bridge rail (includes parapet)
24 = Guardrail face
25 = Concrete traffic barrier
26 = Other traffic barrier
30 = Utility pole/light support
31 = Other post, other pole or other supports
32 = Culvert
33 = Curb
34 = Ditch
35 = Embankment
38 = Fence
39 = Wall
40 = Fire hydrant
41 = Shrubbery
42 = Tree (standing only)
46 = Traffic signal support
48 = Snow bank
50 = Bridge overhead structure
52 = Guardrail end

53 = Mail box
57 = Cable barrier
58 = Ground
59 = Traffic sign support

Variable	Name	Format	Type	Length
v24	MANNER OF COLLISION	MCOLL10F	Numeric	8

N	Prcnt	Code	Label
882	23.8	0	Not a collision with motor vehicle in-transport
683	18.5	1	Front-to-rear
580	15.7	2	Front-to-front
1,191	32.2	6	Angle
134	3.6	7	Sideswipe - same direction
182	4.9	8	Sideswipe - opposite direction
14	0.4	9	Rear-to-side
1	0.0	10	Rear-to-rear
28	0.8	11	Other
3	0.1	98	Not reported
1	0.0	99	Unknown

Variable	Name	Format	Type	Length
v25A	RELATION TO JUNCTION WITHIN INTERCHANGE AREA	RJINT10F	Numeric	8

WITHIN INTERCHANGE AREA

N	Prcnt	Code	Label
3,424	92.6	0	No
244	6.6	1	Yes
31	0.8	8	Not Reported

Variable	Name	Format	Type	Length
v25B	RELATION TO JUNCTION - JUNCTION	RLJCT10F	Numeric	8

SPECIFIC LOCATION

N	Prcnt	Code	Label
2,352	63.6	1	Non-Junction
850	23.0	2	Intersection

N	Prcnt	Code	Label
148	4.0	3	Intersection-Related
28	0.8	4	Driveway Access
76	2.1	5	Entrance/Exit Ramp Related
19	0.5	6	Railway Grade Crossing
22	0.6	7	Crossover-Related
118	3.2	8	Driveway Access Related
3	0.1	17	Acceleration/Deceleration Lane
72	1.9	18	Through Roadway
10	0.3	19	Other location within Interchange Area
1	0.0	98	Not Reported

Variable	Name	Format	Type	Length
v26	RELATION TO ROADWAY	RRDWY10F	Numeric	8

N	Prcnt	Code	Label
3,170	85.7	1	On Roadway
99	2.7	2	On Shoulder
90	2.4	3	On Median
280	7.6	4	On Roadside
29	0.8	5	Outside Trafficway
18	0.5	6	Off Roadway-Location Unknown
5	0.1	8	Gore
7	0.2	10	Separator
1	0.0	98	Not Reported

Variable	Name	Format	Type	Length
v27	TRAFFICWAY DESCRIPTION	TFLOW10F	Numeric	8

A trafficway may include several roadways if it is a physically divided highway. Trafficways are not physically divided unless the divider is a median, barrier, or other constructed device. Pavement markings do qualify when they meet the definition of a media.

N	Prcnt	Code	Label
8	0.2	0	Non-Trafficway Area
1,902	51.4	1	Two-Way, Not Divided
877	23.7	2	Two-Way, Divided, Unprotected (Painted > 4 Feet) Median
707	19.1	3	Two-Way, Divided, Positive Median Barrier
29	0.8	4	One-Way Trafficway

N	Prcnt	Code	Label
120	3.2	5	Two-Way, Not Divided With a Continuous Left-Turn Lane
44	1.2	6	Entrance/Exit Ramp
3	0.1	8	Not Reported
9	0.2	9	Unknown

Variable	Name	Format	Type	Length
v28	TOTAL LANES IN ROADWAY	LANES10F	Numeric	8

A roadway (through lanes only) is one part of a divided trafficway or, if undivided, the same as the through lanes of the trafficway. A lane that can be used for through or turning traffic (dual purpose) will be considered a through lane.

N	Prcnt	Code	Label
8	0.2	0	Non-Trafficway Area
50	1.4	1	One lane
2,617	70.7	2	Two lanes
293	7.9	3	Three lanes
582	15.7	4	Four lanes
57	1.5	5	Five lanes
55	1.5	6	Six lanes
2	0.1	7	Seven or more lanes
13	0.4	8	Not Reported
22	0.6	9	Unknown

Variable	Name	Format	Type	Length
v29	SPEED LIMIT	FSPLMT	Numeric	8

N	Prcnt	Code	Label
10	0.3	0	No statutory limit
1	0.0	5	5 mph
1	0.0	10	10 mph
2	0.1	15	15 mph
2	0.1	20	20 mph
67	1.8	25	25 mph
85	2.3	30	30 mph
155	4.2	35	35 mph
137	3.7	40	40 mph
369	10.0	45	45 mph

N	Prcnt	Code	Label
160	4.3	50	50 mph
1,255	33.9	55	55 mph
203	5.5	60	60 mph
697	18.8	65	65 mph
382	10.3	70	70 mph
84	2.3	75	75 mph
2	0.1	80	80 mph
23	0.6	98	Not Reported
64	1.7	99	Unknown

Variable	Name	Format	Type	Length
v30	ROADWAY ALIGNMENT	VALIG10F	Numeric	8

N	Prcnt	Code	Label
8	0.2	0	Non-Trafficway Area
3,054	82.6	1	Straight
246	6.7	2	Curve Right
291	7.9	3	Curve Left
84	2.3	4	Curve - Unknown Direction
9	0.2	8	Not Reported
7	0.2	9	Unknown

Variable	Name	Format	Type	Length
v31	ROADWAY GRADE	VGRAD10F	Numeric	8

N	Prcnt	Code	Label
8	0.2	0	Non-Trafficway Area
2,571	69.5	1	Level
625	16.9	2	Grade, Unknown Slope
99	2.7	3	Hillcrest
18	0.5	4	Sag (Bottom)
144	3.9	5	Uphill
181	4.9	6	Downhill
37	1.0	8	Not Reported
16	0.4	9	Unknown

Variable	Name	Format	Type	Length
v32	ROADWAY SURFACE TYPE	PAVET10F	Numeric	8

N	Prcnt	Code	Label
8	0.2	0	Non-Trafficway Area
417	11.3	1	Concrete
2,783	75.2	2	Blacktop, Bituminous, or Asphalt
3	0.1	3	Brick or Block
29	0.8	4	Slag, Gravel or Stone
4	0.1	5	Dirt
4	0.1	7	Other
433	11.7	8	Not Reported
18	0.5	9	Unknown

Variable	Name	Format	Type	Length
v33	ROADWAY SURFACE CONDITION	SURFC10F	Numeric	8

N	Prcnt	Code	Label
8	0.2	0	Non-Trafficway Area
3,040	82.2	1	Dry
444	12.0	2	Wet
70	1.9	3	Snow
91	2.5	4	Ice/Frost
2	0.1	5	Sand
9	0.2	6	Water (Standing or Moving)
3	0.1	8	Other
18	0.5	10	Slush
4	0.1	11	Mud, Dirt or Gravel
3	0.1	98	Not Reported
7	0.2	99	Unknown

Variable	Name	Format	Type	Length
v37	LIGHT CONDITION	LGTCD10F	Numeric	8

N	Prcnt	Code	Label
2,371	64.1	1	Daylight
822	22.2	2	Dark - Not Lighted
336	9.1	3	Dark - Lighted

N	Prcnt	Code	Label
85	2.3	4	Dawn
63	1.7	5	Dusk
21	0.6	6	Dark - Unknown Lighting
1	0.0	9	Unknown

Variable	Name	Format	Type	Length
v38	ATMOSPHERIC CONDITIONS	ATMCD10F	Numeric	8

N	Prcnt	Code	Label
2,732	73.9	1	Clear
273	7.4	2	Rain
33	0.9	3	Sleet, Hail (Freezing Rain or Drizzle)
110	3.0	4	Snow
76	2.1	5	Fog, Smog, Smoke
10	0.3	6	Severe Crosswinds
3	0.1	7	Blowing Sand, Soil, Dirt
5	0.1	8	Other
439	11.9	10	Cloudy
11	0.3	11	Blowing Snow
1	0.0	98	Not Reported
6	0.2	99	Unknown

Variable	Name	Format	Type	Length
v39	WORK ZONE	CZONE10F	Numeric	8

Use of this code does not imply the accident was caused by the construction, maintenance or utility activity.

N	Prcnt	Code	Label
3,543	95.8	0	None
120	3.2	1	Construction
23	0.6	2	Maintenance
12	0.3	4	Work Zone, Type Unknown
1	0.0	8	Not Reported

Variable	Name	Format	Type	Length
v40	EMS NOTIFIED - HOUR	NOTHR10F	Numeric	8

N	Prcnt	Code	Label
39	1.1	0	0:00am-0:59am
50	1.4	1	
		-	Hour
49	1.3	23	
350	9.5	88	Not Applicable (Not Notified)
1,147	31.0	99	Unknown

Variable	Name	Format	Type	Length
v41	EMS NOTIFIED - MINUTE	NOTMN10F	Numeric	8

N	Prcnt	Code	Label
36	1.0	0	On the Hour
47	1.3	1	
		-	Minute
32	0.9	59	
350	9.5	88	Not Applicable (Not Notified)
18	0.5	98	Unknown if Notified
1,129	30.5	99	Unknown

Variable	Name	Format	Type	Length
v42	EMS ARRIVAL - HOUR	ARRHR10F	Numeric	8

N	Prcnt	Code	Label
40	1.1	0	0:00 am-0:59am
48	1.3	1	
		-	Hour
50	1.4	23	
350	9.5	88	Not Applicable
1,219	33.0	99	Unknown EMS Scene Arrival Hour

Variable	Name	Format	Type	Length
v43	EMS ARRIVAL - MINUTE	ARRMN10F	Numeric	8

N	Prcnt	Code	Label
44	1.2	0	
		-	Minute
42	1.1	59	
350	9.5	88	Not Applicable
6	0.2	97	Officially Cancelled
19	0.5	98	Unknown if Arrived
1,195	32.3	99	Unknown EMS Scene Arrival Minutes

Variable	Name	Format	Type	Length
v46	SCHOOL BUS RELATED	SCHBS10F	Numeric	8

Identifies crashes in which a school bus was directly or indirectly involved, such as a crash involving children alighting from a school bus. The school bus does not have to be a traffic unit in the crash.

N	Prcnt	Code	Label
3,693	99.8	0	No
6	0.2	1	Yes

Variable	Name	Format	Type	Length
v48	RAIL GRADE CROSSING ID	$RAIL10F	Char	7

RAIL GRADE CROSSING ID – FRA CODE

N	Prcnt	Code	Label
3,677	99.4	0000000	Not Applicable
1	0.0	023114C	
		-	FRA Code
1	0.0	933989B	
5	0.1	9999999	Unknown

Variable	Name	Format	Type	Length
v49	NUMBER FATALITIES IN CRASH	FFATALS	Numeric	8

N	Prcnt	Code	Label
3,317	89.7	1	1 killed
301	8.1	2	2 killed
53	1.4	3	3 killed
20	0.5	4	4 killed
5	0.1	5	5 killed
1	0.0	6	6 killed
1	0.0	7	7 killed
1	0.0	11	11 killed

Variable	Name	Format	Type	Length
v50	DAY OF WEEK	DAYWK10F	Numeric	8

N	Prcnt	Code	Label
238	6.4	1	Sunday
614	16.6	2	Monday
610	16.5	3	Tuesday
603	16.3	4	Wednesday
626	16.9	5	Thursday
644	17.4	6	Friday
364	9.8	7	Saturday

Variable	Name	Format	Type	Length
v51	NUMBER DRINKING DRIVERS	FDRUNKDR	Numeric	8

N	Prcnt	Code	Label
3,164	85.5	0	0 drivers
521	14.1	1	1 driver
11	0.3	2	2 drivers
3	0.1	3	3 drivers

Variable	Name	Format	Type	Length
v52	CRASH DATE - JULIAN		Numeric	8

The Julian date from March 1, 1990.

N	Prcnt	Code	Label
4	0.1	40119	
		-	
7	0.2	40543	

Variable	Name	Format	Type	Length
v53	NUMBER UNINJURED IN CRASH	FUNINJ	Numeric	8

N	Prcnt	Code	Label
1,266	34.2	0	0 uninjured
1,764	47.7	1	1 uninjured
403	10.9	2	2 uninjured
133	3.6	3	3 uninjured
48	1.3	4	4 uninjured
31	0.8	5	5 uninjured
25	0.7	6	6 uninjured
20	0.5	7	7 uninjured
3	0.1	9	9 uninjured
6	0.2	15	15 uninjured

Variable	Name	Format	Type	Length
v54	NUMBER C-INJURED IN CRASH	FCINJ	Numeric	8

N	Prcnt	Code	Label
3,103	83.9	0	0 C-injured
450	12.2	1	1 C-injured
91	2.5	2	2 C-injured
20	0.5	3	3 C-injured
13	0.4	4	4 C-injured
14	0.4	5	5 C-injured
4	0.1	6	6 C-injured
2	0.1	7	7 C-injured
1	0.0	20	20 C-injured
1	0.0	49	49 C-injured

Variable	Name	Format	Type	Length
v55	NUMBER B-INJURED IN CRASH	FBINJ	Numeric	8

N	Prcnt	Code	Label
2,832	76.6	0	0 B-injured
655	17.7	1	1 B-injured
153	4.1	2	2 B-injured
33	0.9	3	3 B-injured
11	0.3	4	4 B-injured
8	0.2	5	5 B-injured
2	0.1	6	6 B-injured
1	0.0	7	7 B-injured
1	0.0	9	9 B-injured
1	0.0	10	10 B-injured
1	0.0	12	12 B-injured
1	0.0	17	17 B-injured

Variable	Name	Format	Type	Length
v56	NUMBER A-INJURED IN CRASH	FAINJ	Numeric	8

N	Prcnt	Code	Label
3,038	82.1	0	0 A-injured
489	13.2	1	1 A-injured
112	3.0	2	2 A-injured
34	0.9	3	3 A-injured
14	0.4	4	4 A-injured
10	0.3	5	5 A-injured
1	0.0	6	6 A-injured
1	0.0	7	7 A-injured

Variable	Name	Format	Type	Length
v57	NUMBER K-INJURED IN CRASH	FKINJ	Numeric	8

N	Prcnt	Code	Label
3,317	89.7	1	1 killed
301	8.1	2	2 killed
53	1.4	3	3 killed
20	0.5	4	4 killed

N	Prcnt	Code	Label
5	0.1	5	5 killed
1	0.0	6	6 killed
1	0.0	7	7 killed
1	0.0	11	11 killed

Variable	Name	Format	Type	Length
v58	NUM UNK INJURED IN CRASH	FUNKINJ	Numeric	8

NUMBER OF UNKNOWN INJURED IN CRASH

N	Prcnt	Code	Label
3,675	99.4	0	0 unknown injured
16	0.4	1	1 unknown injured
2	0.1	2	2 unknown injured
3	0.1	3	3 unknown injured
2	0.1	4	4 unknown injured
1	0.0	6	6 unknown injured

Variable	Name	Format	Type	Length
v59	CRASH RELATED FACTORS #1	ARF10F	Numeric	8

FACTORS AT CRASH LEVEL – RESPONSE #1

N	Prcnt	Code	Label
3,406	92.1	0	None
0	0.0	1	Inadequate Warning of Exits, Lanes Narrowing, Traffic Controls, etc.
22	0.6	3	Other Construction-Created Condition
2	0.1	4	No or Obscured Pavement Marking
3	0.1	5	Surface Under Water
0	0.0	6	Inadequate Construction or Poor Design of Roadway, Bridge, etc.
			Special circumstances
0	0.0	7	Surface Washed Out(caved-in, road slippage)
0	0.0	13	Aggressive Driving / Road Rage by Non-contact Vehicle Driver
120	3.2	14	Motor Vehicle struck by falling cargo, or something that came loose from or something that was set in motion by a vehicle

N	Prcnt	Code	Label
5	0.1	15	Non-occupant struck by falling cargo, or something that came loose from, or something that was set in motion by a vehicle
5	0.1	16	Non-occupant struck vehicle
2	0.1	17	Vehicle set-in-motion by non-driver
4	0.1	18	Date of Crash and Date of EMS Notification were not the same day
93	2.5	19	Recent/Previous Crash scene Nearby
12	0.3	20	Police Pursuit Involved
0	0.0	21	Within Designated School Zone
1	0.0	22	Speed Limit is a Statutory Limit as Recorded or was determined as this state`s `basic rule`
12	0.3	23	Indication of a Stalled/Disabled Vehicle
4	0.1	99	Unknown

Variable	Name	Format	Type	Length
v60	CRASH RELATED FACTORS #2	ARF10F	Numeric	8

FACTORS AT CRASH LEVEL – RESPONSE #2

N	Prcnt	Code	Label
3,665	99.1	0	None
			Special circumstances
2	0.1	14	Motor Vehicle struck by falling cargo, or something that came loose from or something that was set in motion by a vehicle
3	0.1	15	Non-occupant struck by falling cargo, or something that came loose from, or something that was set in motion by a vehicle
13	0.4	19	Recent/Previous Crash scene Nearby
1	0.0	20	Police Pursuit Involved
11	0.3	23	Indication of a Stalled/Disabled Vehicle
4	0.1	99	Unknown

Variable	Name	Format	Type	Length
v61	CRASH RELATED FACTORS #3	ARF10F	Numeric	8

FACTORS AT CRASH LEVEL – RESPONSE #3

N	Prcnt	Code	Label
3,693	99.8	0	None
			Special circumstances
1	0.0	14	Motor Vehicle struck by falling cargo, or something that came
1	0.0	19	Recent/Previous Crash scene Nearby
4	0.1	99	Unknown

Variable	Name	Format	Type	Length
v62	ATMOSPHERIC CONDITIONS #1	ATMCD10F	Numeric	8

N	Prcnt	Code	Label
2,732	73.9	1	Clear
264	7.1	2	Rain
30	0.8	3	Sleet, Hail (Freezing Rain or Drizzle)
106	2.9	4	Snow
76	2.1	5	Fog, Smog, Smoke
9	0.2	6	Severe Crosswinds
2	0.1	7	Blowing Sand, Soil, Dirt
4	0.1	8	Other
456	12.3	10	Cloudy
13	0.4	11	Blowing Snow
1	0.0	98	Not Reported
6	0.2	99	Unknown

Variable	Name	Format	Type	Length
v63	ATMOSPHERIC CONDITIONS #2	ATMCD10F	Numeric	8

N	Prcnt	Code	Label
3,633	98.2	0	No Additional Atmospheric Conditions
4	0.1	1	Clear
13	0.4	2	Rain
5	0.1	3	Sleet, Hail (Freezing Rain or Drizzle)

N	Prcnt	Code	Label
4	0.1	4	Snow
4	0.1	5	Fog, Smog, Smoke
3	0.1	6	Severe Crosswinds
1	0.0	7	Blowing Sand, Soil, Dirt
1	0.0	8	Other
23	0.6	10	Cloudy
8	0.2	11	Blowing Snow

Variable	Name	Format	Type	Length
v71	LATITUDE	13.8	Numeric	8

LATITUDE IN DECIMAL DEGREE FORMAT

N	Prcnt	Code	Label
1	0.0	19.157817	
		-	Latitude
1	0.0	64.858708	

Variable	Name	Format	Type	Length
v72	LONGITUDE		Numeric	8

LONGITUDE IN DECIMAL DEGREE FORMAT

N	Prcnt	Code	Label
1	0.0	-159.5031	
		-	Longitude
1	0.0	-68.16613	

Variable	Name	Format	Type	Length
v80	TYPE OF INTERSECTION	INTTY10F	Numeric	8

N	Prcnt	Code	Label
2,720	73.5	1	Not an Intersection
675	18.2	2	Four-Way Intersection
255	6.9	3	T-Intersection
32	0.9	4	Y-Intersection
1	0.0	6	Roundabout
5	0.1	7	Five Point, or More

N	Prcnt	Code	Label
5	0.1	8	Not Reported
6	0.2	9	Unknown

The VEHICLE Variables

Variables 104 through 186 describe the vehicle involved in the accident (i.e., the truck).

Variable	Name	Format	Type	Length
v104	VEHICLE NUMBER	FVEHNUM	Numeric	4

N	Prcnt	Code	Label
1,662	44.9	1	Vehicle #1
1,754	47.4	2	Vehicle #2
211	5.7	3	Vehicle #3
41	1.1	4	Vehicle #4
14	0.4	5	Vehicle #5
6	0.2	6	Vehicle #6
2	0.1	7	Vehicle #7
1	0.0	8	Vehicle #8
2	0.1	9	Vehicle #9
1	0.0	10	Vehicle #10
3	0.1	11	Vehicle #11
1	0.0	12	Vehicle #12
1	0.0	13	Vehicle #13

Variable	Name	Format	Type	Length
v105	UNIT TYPE	UNIT10F	Numeric	8

N	Prcnt	Code	Label
3,699	100.0	1	Motor Vehicle In-Transport (Inside or Outside the Trafficway)
0	0.0	2	Motor Vehicle Not In-Transport Within the Trafficway
0	0.0	3	Motor Vehicle Not In-Transport Outside the Trafficway
0	0.0	4	Working Motor Vehicle (highway construction, maintenance, utility only)

Variable	Name	Format	Type	Length
v107	NUMBER OF OCCUPANTS	FNUMOCC	Numeric	8

N	Prcnt	Code	Label
37	1.0	0	0 occupants
3,160	85.4	1	1 occupant
412	11.1	2	2 occupants

N	Prcnt	Code	Label
60	1.6	3	3 occupants
19	0.5	4	4 occupants
9	0.2	5	5 occupants
1	0.0	6	6 occupants
1	0.0	9	9 occupants

Variable	Name	Format	Type	Length
v108	VEHICLE MAKE	MAKE10F	Numeric	8

N	Prcnt	Code	Label
122	3.3	7	Dodge
356	9.6	12	ford
86	2.3	20	Chevrolet
82	2.2	23	GMC
34	0.9	38	Isuzu
247	6.7	51	Volvo
5	0.1	52	Mitsubishi
866	23.4	82	Freightliner
543	14.7	84	International Harvester/Navistar
416	11.2	85	Kenworth
261	7.1	86	Mack
452	12.2	87	Peterbilt
1	0.0	88	Iveco/Magirus
33	0.9	89	White/Autocar White/GMC
183	4.9	98	Other Make
12	0.3	99	Unknown Make

Variable	Name	Format	Type	Length
v109	VEHICLE MAKE-MODEL	11	Numeric	8

N	Prcnt	Code	Label
2	0.1	7461	B-series van/Ram van/Ram wagon
2	0.1	7481	D, W-series pickup
73	2.0	7482	Dodge Ram pickup
34	0.9	7880	Dodge medium/heavy pickup
11	0.3	7881	Dodge medium/heavy -- cab behind engine
1	0.0	12016	Ford Crown Victoria

N	Prcnt	Code	Label
20	0.5	12461	Ford E-series van/Econoline
106	2.9	12481	Ford F-series pickup
3	0.1	12870	Ford medium/heavy van-based vehicle
51	1.4	12880	Ford medium/heavy pickup (pickup-style only - over 10,000 lbs)
165	4.5	12881	Ford medium/heavy -- cab behind engine
3	0.1	12882	Ford medium/heavy -- cab over engine low entry
5	0.1	12884	Ford medium/heavy -- unknown engine location
1	0.0	12898	Ford other (medium/heavy truck)
1	0.0	12981	Ford conventional bus (engine out front)
1	0.0	20398	Chevrolet other (auto)
6	0.2	20461	Chevrolet G-series van
27	0.7	20481	Chevrolet C, K, R, V-series pickup/Silverado
9	0.2	20880	Chevrolet medium/heavy pickup (pickup style only, over 10,000 lbs.)
36	1.0	20881	Chevrolet medium/heavy -- cab behind engine
3	0.1	20882	Chevrolet medium/heavy -- cab over engine low entry
3	0.1	20898	Chevrolet other (medium/heavy truck)
1	0.0	20899	Chevrolet unknown (medium/heavy truck)
4	0.1	23461	GMC G-series van/Savana
1	0.0	23470	GMC van derivative
11	0.3	23481	GMC C, K, R, V-series pickup/Sierra
1	0.0	23870	GMC medium/heavy van-based vehicle
7	0.2	23880	GMC medium/heavy pickup (pickup style only, over 10,000 lbs.)
45	1.2	23881	GMC medium/heavy -- cab behind engine
9	0.2	23882	GMC medium/heavy -- cab over engine low entry
3	0.1	23884	GMC medium/heavy -- unknown engine location
1	0.0	23898	GMC other (medium/heavy truck)
33	0.9	38882	Isuzu medium/heavy -- cab over engine low entry
1	0.0	38898	Isuzu other (medium/heavy truck)
220	5.9	51881	Volvo medium/heavy -- cab behind engine
4	0.1	51882	Volvo medium/heavy -- cab over engine low entry
6	0.2	51883	Volvo medium/heavy -- cab over engine high entry
14	0.4	51884	Volvo medium/heavy -- unknown engine location
1	0.0	51890	Volvo medium/heavy -- cab over engine unknown entry position
1	0.0	51898	Volvo other (medium/heavy truck)
1	0.0	51899	Volvo unknown (medium/heavy truck)
4	0.1	52882	Mitsubishi medium/heavy -- cab over engine low entry
1	0.0	52898	Mitsubishi other (medium/heavy truck)
554	15.0	82881	Freightliner medium/heavy -- cab behind engine

N	Prcnt	Code	Label
1	0.0	82882	Freightliner medium/heavy -- cab over engine low entry
245	6.6	82883	Freightliner medium/heavy -- cab over engine high entry
50	1.4	82884	Freightliner medium/heavy -- unknown engine location
3	0.1	82890	Freightliner medium/heavy -- cab over engine unknown entry position
1	0.0	82898	Freightliner other (medium/heavy truck)
12	0.3	82899	Freightliner unknown (medium/heavy truck)
446	12.1	84881	International Harvester/Navistar medium/heavy -- cab behind engine
2	0.1	84882	International Harvester/Navistar medium/heavy -- cab over engine low entry
35	0.9	84883	International Harvester/Navistar medium/heavy -- cab over engine high entry
48	1.3	84884	International Harvester/Navistar medium/heavy -- unknown engine location
2	0.1	84890	International Harvester/Navistar medium/heavy -- cab over engine unknown entry position
8	0.2	84899	International Harvester/Navistar unknown (medium/heavy truck)
2	0.1	84981	International Harvester/Navistar bus: conventional (engine out front)
392	10.6	85881	Kenworth medium/heavy -- cab behind engine
3	0.1	85882	Kenworth medium/heavy -- cab over engine low entry
3	0.1	85883	Kenworth medium/heavy -- cab over engine high entry
13	0.4	85884	Kenworth medium/heavy -- unknown engine location
3	0.1	85898	Kenworth other (medium/heavy truck)
2	0.1	85899	Kenworth unknown (medium/heavy truck)
214	5.8	86881	Mack medium/heavy -- cab behind engine
25	0.7	86882	Mack medium/heavy -- cab over engine low entry
1	0.0	86883	Mack medium/heavy -- cab over engine high entry
16	0.4	86884	Mack medium/heavy -- unknown engine location
1	0.0	86890	Mack medium/heavy -- cab over engine unknown entry position
1	0.0	86898	Mack other (medium/heavy truck)
2	0.1	86899	Mack unknown (medium/heavy truck)
1	0.0	86999	Unknown Mack
398	10.8	87881	Peterbilt medium/heavy -- cab behind engine
3	0.1	87882	Peterbilt medium/heavy -- cab over engine low entry
26	0.7	87883	Peterbilt medium/heavy -- cab over engine high entry
18	0.5	87884	Peterbilt medium/heavy -- unknown engine location
3	0.1	87890	Peterbilt medium/heavy -- cab over engine unknown entry
1	0.0	87898	Peterbilt other (medium/heavy truck)
3	0.1	87899	Peterbilt unknown (medium/heavy truck)

N	Prcnt	Code	Label
1	0.0	88881	Iveco medium/heavy -- cab behind engine
22	0.6	89881	White/Autocar-White/GMC medium/heavy -- cab behind engine
	0.2	89882	White/Autocar-White/GMC medium/heavy -- cab over engine low entry
3	0.1	89884	White/Autocar-White/GMC medium/heavy -- unknown engine location
1	0.0	89898	White/Autocar-White/GMC other (medium/heavy truck)
52	1.4	98804	Other make Western Star
16	0.4	98806	Other make Hino
14	0.4	98808	Other make UD
78	2.1	98809	Other make Sterling
11	0.3	98881	Other make medium/heavy -- cab behind engine
1	0.0	98882	Other make medium/heavy -- cab over engine low entry
2	0.1	98884	Other make medium/heavy -- unknown engine location
1	0.0	98890	Other make medium/heavy -- cab over engine entry position unknown
8	0.2	98898	Other make other (medium/heavy truck)
1	0.0	99881	Unknown make medium/heavy -- cab behind engine
9	0.2	99884	Unknown make medium/heavy -- unknown engine location
2	0.1	99898	Unknown make other (medium/heavy truck)

Variable	Name	Format	Type	Length
v110	BODY TYPE	BODY10F	Numeric	8

N	Prcnt	Code	Label
1	0.0	4	4-door sedan, hardtop
1	0.0	9	Other or Unknown automobile type
29	0.8	21	Large Van (B150-B350, Sportsman, Royal Maxiwagon, Ram, Tradesman, Voyager [83 and before], ...)
3	0.1	22	Step-van or walk-in van (<= 10,000 lbs. GVWR)
1	0.0	29	Unknown van type
210	5.7	31	Standard pickup (GVWR 4,500 to 10,000 lbs.)(Jeep Pickup, Comanche, Ram Pickup, D100-D350, ...)
1	0.0	39	Unknown (pickup style) light conventional truck type
8	0.2	40	Cab Chassis Based (includes Rescue Vehicle, Light Stake, Dump, and Tow Truck)
2	0.1	50	School Bus
1	0.0	59	Unknown Bus Type
16	0.4	60	Step van (>10,000 lbs. GVWR)
200	5.4	61	Single unit straight truck (10,000 lbs < GVWR <= 19,500 lbs)

N	Prcnt	Code	Label
178	4.8	62	Single unit straight truck (19,500 lbs < GVWR <= 26,000 lbs.)
544	14.7	63	Single unit straight truck (GVWR > 26,000 lbs.)
4	0.1	64	Single unit straight truck (GVWR unknown)
2,389	64.6	66	Truck-tractor (Cab only, or with any number of trailing unit; any weight)
101	2.7	67	Medium/Heavy Pickup (Ford Super Duty 450/550)
1	0.0	71	Unknown if single unit or combination unit Medium Truck (10,000 lbs. < GVWR < 26,000 lbs.)
2	0.1	72	Unknown if single unit or combination unit Heavy Truck (GVWR > 26,000 lbs.)
6	0.2	78	Unknown medium/heavy truck type
1	0.0	99	Unknown body type

Variable	Name	Format	Type	Length
v111	MODEL YEAR	FMODYR	Numeric	8

N	Prcnt	Code	Label
1	0.0	1959	
0		-	Actual model year
28	0.8	1991	1991
31	0.8	1992	1992
59	1.6	1993	1993
62	1.7	1994	1994
95	2.6	1995	1995
99	2.7	1996	1996
106	2.9	1997	1997
135	3.6	1998	1998
218	5.9	1999	1999
282	7.6	2000	2000
188	5.1	2001	2001
139	3.8	2002	2002
168	4.5	2003	2003
196	5.3	2004	2004
345	9.3	2005	2005
356	9.6	2006	2006
449	12.1	2007	2007
204	5.5	2008	2008
189	5.1	2009	2009
135	3.6	2010	2010

N	Prcnt	Code	Label
36	1.0	2011	2011
14	0.4	9999	Unknown

Variable	Name	Format	Type	Length
v112	VIN	$VIN10F	Char	12

VEHICLE IDENTIFICATION NUMBER – FIRST 12 POSITIONS

Variable	Name	Format	Type	Length
v123	REGISTRATION STATE	REGST10F	Numeric	8

N	Prcnt	Code	Label
11	0.3	0	Not Applicable
79	2.1	1	Alabama
5	0.1	2	Alaska
41	1.1	4	Arizona
61	1.6	5	Arkansas
190	5.1	6	California
39	1.1	8	Colorado
12	0.3	9	Connecticut
7	0.2	10	Delaware
188	5.1	12	Florida
105	2.8	13	Georgia
1	0.0	14	Guam
6	0.2	15	Hawaii
20	0.5	16	Idaho
178	4.8	17	Illinois
248	6.7	18	Indiana
86	2.3	19	Iowa
53	1.4	20	Kansas
75	2.0	21	Kentucky
74	2.0	22	Louisiana
19	0.5	23	Maine
30	0.8	24	Maryland
18	0.5	25	Massachusetts
69	1.9	26	Michigan
85	2.3	27	Minnesota

N	Prcnt	Code	Label
53	1.4	28	Mississippi
85	2.3	29	Missouri
14	0.4	30	Montana
48	1.3	31	Nebraska
10	0.3	32	Nevada
8	0.2	33	New Hampshire
62	1.7	34	New Jersey
18	0.5	35	New Mexico
97	2.6	36	New York
115	3.1	37	North Carolina
11	0.3	38	North Dakota
123	3.3	39	Ohio
130	3.5	40	Oklahoma
26	0.7	41	Oregon
127	3.4	42	Pennsylvania
3	0.1	44	Rhode Island
53	1.4	45	South Carolina
24	0.6	46	South Dakota
90	2.4	47	Tennessee
284	7.7	48	Texas
29	0.8	49	Utah
10	0.3	50	Vermont
47	1.3	51	Virginia
46	1.2	53	Washington
28	0.8	54	West Virginia
62	1.7	55	Wisconsin
13	0.4	56	Wyoming
1	0.0	91	Not Reported
29	0.8	92	No Registration
288	7.8	93	Multiple State Registration
7	0.2	94	U.S. Government Tags (includes military)
33	0.9	95	Canada
2	0.1	96	Mexico
23	0.6	99	Unknown

Variable	Name	Format	Type	Length
v124	REGISTERED VEHICLE OWNER	OWNER10F	Numeric	8

N	Prcnt	Code	Label
39	1.1	0	Not Applicable, Vehicle Not Registered
533	14.4	1	Driver (in this crash) was Registered Owner
296	8.0	2	Driver (in this crash) Not Registered Owner (Other Private Owner Listed)
2,728	73.7	3	Vehicle Registered as Business/Company/Government Vehicle
42	1.1	4	Vehicle Registered as Rental Vehicle
1	0.0	5	Vehicle was Stolen (reported by police)
28	0.8	6	Driverless/Motor Vehicle Parked/Stopped Off Roadway
32	0.9	9	Unknown

Variable	Name	Format	Type	Length
v125	ROLLOVER	ROLL10F	Numeric	8

N	Prcnt	Code	Label
3,215	86.9	0	No Rollover
409	11.1	1	Rollover, Tripped by Object/Vehicle
70	1.9	2	Rollover, Untripped
5	0.1	9	Rollover, Unknown Type

Variable	Name	Format	Type	Length
v125B	LOCATION OF ROLLOVER	RLOCT10F	Numeric	8

N	Prcnt	Code	Label
3,215	86.9	0	No Rollover
122	3.3	1	On Roadway
28	0.8	2	On Shoulder
43	1.2	3	On Median/Separator
6	0.2	4	In Gore
244	6.6	5	On Roadside
39	1.1	6	Outside of Trafficway
2	0.1	9	Unknown

Variable	Name	Format	Type	Length
v126	JACKKNIFE	JACKK10F	Numeric	8

Identifies the loss of control of a truck in motion where the trailing unit yaws more than 15 degrees from its normal straight line path behind the cab.

N	Prcnt	Code	Label
1,163	31.4	0	Not an Articulated Vehicle
2,356	63.7	1	No
30	0.8	2	Yes - First Event
150	4.1	3	Yes - Subsequent Event

Variable	Name	Format	Type	Length
v127	TRAVEL SPEED	TSPED10F	Numeric	8

N	Prcnt	Code	Label
241	6.5	0	Stopped Motor Vehicle In- Transport
1	0.0	1	
		-	Actual miles per hour
1	0.0	100	
1,615	43.7	998	Not Reported
272	7.4	999	Unknown

Variable	Name	Format	Type	Length
v129	VEHICLE TRAILERING	VTOW10F	Numeric	8

Trailing unit applies to any device connected to a motor vehicle by a hitch, including tractor-trailer combinations, boat hitched onto a motor vehicle, etc. This does not include towed vehicles, such as a tow truck pulling a vehicle.

N	Prcnt	Code	Label
1,154	31.2	0	No Trailing Units
2,430	65.7	1	One Trailing Unit
94	2.5	2	Two Trailing Units
6	0.2	3	Three or More Trailing Units
1	0.0	4	Yes, Number of Trailing Units Unknown
9	0.2	5	Vehicle Towing Another Motor Vehicle - Fixed Linkage
2	0.1	6	Vehicle Towing Another Motor Vehicle - Non-Fixed Linkage
3	0.1	9	Unknown

Variable	Name	Format	Type	Length
v130	VEHICLE CONFIGURATION	VCONF10F	Numeric	8

N	Prcnt	Code	Label
219	5.9	0	Not Applicable
575	15.5	1	Single-Unit Truck (2- axle and GVWR more than 10,000 lbs)
392	10.6	2	Single-Unit Truck (3 or more axles)
107	2.9	4	Truck Pulling Trailer(s)
66	1.8	5	Truck Tractor (Bobtail)
2,162	58.4	6	Truck Tractor/Semi-Trailer
94	2.5	7	Truck Tractor/Double
5	0.1	8	Truck Tractor/Triple
63	1.7	19	Truck More Than 10,000 lbs, Cannot Classify
4	0.1	20	Bus/Large Van (seats for 9-15 occupants, including driver)
2	0.1	21	Bus (seats for more than 15 occupants, including driver)
5	0.1	98	Not Reported
5	0.1	99	Unknown

Variable	Name	Format	Type	Length
v132	CARGO BODY TYPE	CARBT10F	Numeric	8

N	Prcnt	Code	Label
220	5.9	0	Not Applicable (N/A)
1,564	42.3	1	Van/Enclosed Box
295	8.0	2	Cargo Tank
405	10.9	3	Flatbed
350	9.5	4	Dump
20	0.5	5	Concrete Mixer
27	0.7	6	Auto Transporter
88	2.4	7	Garbage/Refuse
120	3.2	8	Grain/ Chips/ Gravel
20	0.5	9	Pole-Trailer
65	1.8	10	Log
15	0.4	11	Intermodal Container Chassis
8	0.2	12	Vehicle Towing Another Motor Vehicle
6	0.2	22	Bus
13	0.4	28	Not Reported
190	5.1	96	No Cargo Body Type
158	4.3	97	Other

N	Prcnt	Code	Label
129	3.5	98	Unknown Cargo Body Type
6	0.2	99	Unknown

Variable	Name	Format	Type	Length
v133	SPECIAL USE	SPECU10F	Numeric	8

Indicates that the vehicle was used for a function other than the primary function for which it was designed.

N	Prcnt	Code	Label
3,696	99.9	0	No Special Use
0	0.0	1	Taxi
0	0.0	2	Vehicle Used as School Bus
0	0.0	3	Vehicle Used as Other Bus
2	0.1	4	Military
0	0.0	5	Police
1	0.0	6	Ambulance
0	0.0	7	Fire Truck
0	0.0	8	Emergency Services Vehicle
0	0.0	98	Not Reported
0	0.0	99	Unknown

Variable	Name	Format	Type	Length
v134	EMERGENCY USE	EMERG10F	Numeric	8

Refers to a vehicle traveling with physical emergency signals in use, such as red light blinking, siren sounding, etc.

N	Prcnt	Code	Label
3,699	100.0	0	No
0	0.0	1	Yes

Variable	Name	Format	Type	Length
v135	AREAS OF IMPACT - INITIAL DAMAGE	IMPAC10F	Numeric	8

N	Prcnt	Code	Label
142	3.8	0	Non-Collision
136	3.7	1	1 Clock Point

N	Prcnt	Code	Label
51	1.4	2	2 Clock Point
91	2.5	3	3 Clock Point
40	1.1	4	4 Clock Point
70	1.9	5	5 Clock Point
430	11.6	6	6 Clock Point
91	2.5	7	7 Clock Point
76	2.1	8	8 Clock Point
147	4.0	9	9 Clock Point
83	2.2	10	10 Clock Point
167	4.5	11	11 Clock Point
1,938	52.4	12	12 Clock Point
5	0.1	13	Top
52	1.4	14	Undercarriage
19	0.5	18	Set-In-Motion (Not at Clock Point)
25	0.7	61	Left
20	0.5	62	Left-Front Half
29	0.8	63	Left-Back Half
15	0.4	81	Right
26	0.7	82	Right-Front Half
18	0.5	83	Right-Back Half
7	0.2	98	Not Reported
21	0.6	99	Unknown

Variable	Name	Format	Type	Length
v136	AREAS OF IMPACT - MOST DAMAGE	IMPAC10F	Numeric	8

N	Prcnt	Code	Label
104	2.8	0	Non-Collision
125	3.4	1	1 Clock Point
51	1.4	2	2 Clock Point
97	2.6	3	3 Clock Point
37	1.0	4	4 Clock Point
63	1.7	5	5 Clock Point
400	10.8	6	6 Clock Point
88	2.4	7	7 Clock Point
78	2.1	8	8 Clock Point
155	4.2	9	9 Clock Point
79	2.1	10	10 Clock Point

N	Prcnt	Code	Label
163	4.4	11	11 Clock Point
1,823	49.3	12	12 Clock Point
56	1.5	13	Top
67	1.8	14	Undercarriage
18	0.5	18	Set-In-Motion (Not at Clock Point)
32	0.9	61	Left
31	0.8	62	Left-Front Half
25	0.7	63	Left-Back Half
20	0.5	81	Right
22	0.6	82	Right-Front Half
22	0.6	83	Right-Back Half
90	2.4	98	Not Reported
53	1.4	99	Unknown

Variable	Name	Format	Type	Length
v137	EXTENT OF DAMAGE	DEFOR10F	Numeric	8

N	Prcnt	Code	Label
185	5.0	0	No Damage
630	17.0	2	Minor Damage
628	17.0	4	Functional Damage
2,209	59.7	6	Disabling Damage
6	0.2	8	Not Reported
41	1.1	9	Unknown

Variable	Name	Format	Type	Length
v139	VEHICLE REMOVAL	TOWED10F	Numeric	8

N	Prcnt	Code	Label
836	22.6	1	Driven Away
2,200	59.5	2	Towed Due to Disabling Damage
568	15.4	3	Towed Not Due to Disabling Damage
14	0.4	4	Abandoned/Left at Scene
27	0.7	8	Not Reported
54	1.5	9	Unknown

Variable	Name	Format	Type	Length
v140	FIRE OCCURRENCE	FIRE10F	Numeric	8

N	Prcnt	Code	Label
3,486	94.2	0	No or Not Reported
213	5.8	1	Yes

Variable	Name	Format	Type	Length
v144	MOST HARMFUL EVENT	MHARM10F	Numeric	8

N	Prcnt	Code	Label
227	6.1	1	Rollover/Overturn
109	2.9	2	Fire/Explosion
2	0.1	3	Immersion
13	0.4	5	Fell/Jumped from Vehicle
2	0.1	7	Other Non-Collision
265	7.2	8	Pedestrian
62	1.7	9	Pedalcyclist
14	0.4	10	Railway Vehicle
2	0.1	11	Live Animal
2,751	74.4	12	Motor Vehicle In-Transport
15	0.4	14	Parked Motor Vehicle
11	0.3	15	Non-Motorist on Personal Conveyance
1	0.0	16	Thrown or Falling Object
6	0.2	18	Other Object (not fixed)
1	0.0	19	Building
6	0.2	21	Bridge Pier or Support
5	0.1	23	Bridge Rail (Includes parapet)
20	0.5	24	Guardrail Face
2	0.1	25	Concrete Traffic Barrier
1	0.0	26	Other Traffic Barrier
6	0.2	30	Utility Pole/Light Support
0	0.0	31	Other Post, Other Pole or Other Supports
4	0.1	32	Culvert
9	0.2	34	Ditch
10	0.3	35	Embankment
2	0.1	38	Fence
6	0.2	39	Wall
1	0.0	40	Fire Hydrant

N	Prcnt	Code	Label
69	1.9	42	Tree (Standing Only)
2	0.1	43	Other Fixed Object
5	0.1	45	Working Motor Vehicle
1	0.0	46	Traffic Signal Support
2	0.1	50	Bridge Overhead Structure
3	0.1	51	Jackknife (harmful to this vehicle)
4	0.1	52	Guardrail End
48	1.3	54	Motor Vehicle In-Transport Strikes or is Struck by Cargo, Persons or Objects Set-in-Motion from/by Another Motor Vehicle In Transport
3	0.1	57	Cable Barrier
1	0.0	58	Ground
2	0.1	59	Traffic Sign Support
4	0.1	72	Cargo/Equipment Loss or Shift (harmful to this vehicle)
2	0.1	99	Unknown

The following list shows the code values given above grouped by collision type.

Non-collision Harmful Events
- 01 = Rollover/overturn
- 02 = Fire/explosion
- 03 = Immersion
- 04 = Gas inhalation
- 05 = Fell/jumped from vehicle
- 06 = Injured in vehicle (non-collision)
- 07 = Other non-collision
- 16 = Thrown or falling object
- 44 = Pavement surface irregularity (ruts, potholes, grates, etc.)
- 51 = Jackknife (harmful to this vehicle)
- 72 = Cargo/equipment loss or shift (harmful to this vehicle)

Collision with Motor Vehicle
- 12 = Motor vehicle in–transport
- 54 = Motor vehicle in–transport strikes or is struck by cargo, persons, or objects set–in–motion from/by another motor vehicle in–transport
- 55 = Motor vehicle in motion outside the trafficway

Collision with Object Not Fixed
- 08 = Pedestrian
- 09 = Pedalcyclist
- 10 = Railway vehicle

11 = Live animal
14 = Parked motor vehicle
15 = Nonmotorist on personal conveyance
18 = Other object (not fixed)
45 = Working motor vehicle
49 = Ridden animal or animal–drawn conveyance

Collision with Fixed Object
17 = Boulder
19 = Building
20 = Impact attenuator/crash cushion
21 = Bridge pier or support
23 = Bridge rail (includes parapet)
24 = Guardrail face
25 = Concrete traffic barrier
26 = Other traffic barrier
30 = Utility pole/light support
31 = Other post, other pole or other supports
32 = Culvert
33 = Curb
34 = Ditch
35 = Embankment
38 = Fence
39 = Wall
40 = Fire hydrant
41 = Shrubbery
42 = Tree (standing only)
46 = Traffic signal support
48 = Snow bank
50 = Bridge overhead structure
52 = Guardrail end
53 = Mail box
57 = Cable barrier
58 = Ground
59 = Traffic sign support

Variable	Name	Format	Type	Length
v145	NUMBER OF DEATHS IN VEH	FVDEATH	Numeric	8

NUMBER OF DEATHS IN VEHICLE

N	Prcnt	Code	Label
3,115	84.2	0	0 deaths

N	Prcnt	Code	Label
560	15.1	1	1 death
23	0.6	2	2 deaths
1	0.0	3	3 deaths

Variable	Name	Format	Type	Length
v151	VIN TRUCK FUEL CODE	V151_F	Numeric	4

N	Prcnt	Code	Label
0	0.0	1	(E) Electric operated
117	3.2	2	(G) Gas
3,490	94.3	3	(D) Diesel
1	0.0	4	(P) Propane
0	0.0	7	(*) Not available from vehicle identification number
0	0.0	8	(b) Unknown
91	2.5	9	(9) No vehicle identification number information

Variable	Name	Format	Type	Length
v152	VIN TRUCK WEIGHT CODE	FWGTCD	Numeric	8

N	Prcnt	Code	Label
81	2.2	0	Value not returned
374	10.1	3	10,001 - 14,000 lbs.
95	2.6	4	14,001 - 16,000 lbs.
83	2.2	5	16,001 - 19,500 lbs.
174	4.7	6	19,501 - 26,000 lbs.
236	6.4	7	26,001 - 33,000 lbs.
2,656	71.8	8	33,001 lbs. or more

Variable	Name	Format	Type	Length
v153	VIN TRUCK SERIES	$3	Char	3

Variable	Name	Format	Type	Length
v155	LENGTH OF VIN	6	Numeric	8

N	Prcnt	Code	Label
19	0.5	.	Actual length
3,680	99.5	17	17

Variable	Name	Format	Type	Length
v156	NUMBER UNINJURED IN VEH	FVUNINJ	Numeric	8

NUMBER UNINJURED IN VEHICLE

N	Prcnt	Code	Label
1,463	39.6	0	0 uninjured
1,964	53.1	1	1 uninjured
227	6.1	2	2 uninjured
32	0.9	3	3 uninjured
9	0.2	4	4 uninjured
3	0.1	5	5 uninjured
1	0.0	9	9 uninjured

Variable	Name	Format	Type	Length
v157	NUMBER C-INJURED IN VEH	FVCINJ	Numeric	8

NUMBER C-INJURED IN VEHICLE

N	Prcnt	Code	Label
3,332	90.1	0	0 C-injured
344	9.3	1	1 C-injured
22	0.6	2	2 C-injured
1	0.0	4	4 C-injured

Variable	Name	Format	Type	Length
v158	NUMBER B-INJURED IN VEH	FVBINJ	Numeric	8

NUMBER B-INJURED IN VEHICLE

N	Prcnt	Code	Label
3,221	87.1	0	0 B-injured

N	Prcnt	Code	Label
437	11.8	1	1 B-injured
38	1.0	2	2 B-injured
2	0.1	3	3 B-injured
1	0.0	5	5 B-injured

Variable	Name	Format	Type	Length
v159	NUMBER A-INJURED IN VEH	FVAINJ	Numeric	8

NUMBER A-INJURED IN VEHICLE

N	Prcnt	Code	Label
3,539	95.7	0	0 A-injured
141	3.8	1	1 A-injured
15	0.4	2	2 A-injured
3	0.1	3	3 A-injured
1	0.0	4	4 A-injured

Variable	Name	Format	Type	Length
v160	NUMBER K-INJURED IN VEH	FVKINJ	Numeric	8

NUMBER K-INJURED IN VEHICLE

N	Prcnt	Code	Label
3,115	84.2	0	0 killed
560	15.1	1	1 killed
23	0.6	2	2 killed
1	0.0	3	3 killed

Variable	Name	Format	Type	Length
v161	NUM UNK INJURED IN VEH	FVUNKINJ	Numeric	8

NUMBER UNKNOWN INJURED IN VEHICLE

N	Prcnt	Code	Label
3,690	99.8	0	0 unknown injured
9	0.2	1	1 unknown injured

Variable	Name	Format	Type	Length
v164	UNDERRIDE/OVERRIDE	UORID10F	Numeric	8

N	Prcnt	Code	Label
3,552	96.0	0	No Underride or Override
14	0.4	1	Underriding a Motor Vehicle In-Transport, Underride, Compartment Intrusion
3	0.1	2	Underriding a Motor Vehicle In-Transport, Underride, No Compartment Intrusion
4	0.1	3	Underriding a Motor Vehicle In-Transport, Underride, Compartment Intrusion Unknown
2	0.1	4	Underriding a Motor Vehicle Not In-Transport, Underride, Compartment Intrusion
116	3.1	7	Overriding a Motor Vehicle In-Transport
8	0.2	9	Unknown if Underride or Override

Variable	Name	Format	Type	Length
v165	TRAFFIC CONTROL DEVICE	TRCN10F	Numeric	8

N	Prcnt	Code	Label
2,855	77.2	0	No Controls
23	0.6	1	Traffic control signal (on colors) without Pedestrian Signal
35	0.9	2	Traffic control signal (on colors) with Pedestrian Signal
246	6.7	3	Traffic control signal(on colors) not known whether or not Pedestrian Signal
21	0.6	4	Flashing Traffic Control Signal
8	0.2	8	Other Highway Traffic Signal
2	0.1	9	Unknown Highway Traffic Signal
157	4.2	20	Stop Sign
10	0.3	21	Yield Sign
171	4.6	28	Other Regulatory Sign
1	0.0	29	Unknown Regulatory Sign
75	2.0	40	Warning Sign
11	0.3	50	Person
19	0.5	65	Railway Crossing Device
12	0.3	97	Not Reported
49	1.3	98	Other
4	0.1	99	Unknown

Variable	Name	Format	Type	Length
v166	DEVICE FUNCTIONING	TCFUN10F	Numeric	8

N	Prcnt	Code	Label
2,855	77.2	0	No Controls
2	0.1	1	Device Not Functioning
1	0.0	2	Device Functioning-Functioning Improperly
806	21.8	3	Device Functioning Properly
30	0.8	8	Not Reported
5	0.1	9	Unknown

Variable	Name	Format	Type	Length
v167_1	VEHICLE RELATED FACTORS #1	VRF10F	Numeric	8

FACTORS AT THE VEHICLE LEVEL – RESPONSE #1

N	Prcnt	Code	Label
3,669	99.2	0	None
1	0.0	32	Vehicle Registration for Handicapped
1	0.0	33	Vehicle Being Pushed by Non-Motorist
3	0.1	35	Reconstructed/Altered Vehicle
0	0.0	36	Electric/Alternative Fuel Vehicle
0	0.0	37	Transporting Children To/From Head Start/Day Care
5	0.1	39	Highway Construction, Maintenance, or Utility Vehicle, In Transport (Inside or Outside Work zone)
0	0.0	40	Highway Incident Response Vehicle
0	0.0	41	Police, Fire, or EMS Vehicle Working at the Scene of an Emergency or Performing Other Traffic Control Activities
15	0.4	42	Other Working Vehicle (Not Construction, Maintenance, Utility, Police, Fire, or EMS Vehicle)
0	0.0	44	Adaptive Equipment
5	0.1	99	Unknown

Variable	Name	Format	Type	Length
v167_2	VEHICLE RELATED FACTORS #2	VRF10F	Numeric	8

FACTORS AT VEHICLE LEVEL – RESPONSE #2

N	Prcnt	Code	Label
3,694	99.9	0	None
5	0.1	99	Unknown

Variable	Name	Format	Type	Length
v170	MOTOR CARRIER ID	$11	Char	11

N	Prcnt	Code	Label
219	5.9	00	Not applicable
7	0.2	01	
		-	FARS state code
49	1.3	56	
2453	66.3	57	US DOT
26	0.7	58	MC/MX (ICC)
202	5.5	77	Not reported
266	7.2	88	None
3	0.1	95	Canada
194	5.2	99	Unknown

Identification number (columns 3-11)

0	Not applicable
777777777	Not reported
888888888	None
999999999	Unknown

Variable	Name	Format	Type	Length
v171	BUS USE	BUSUS10F	Numeric	8

N	Prcnt	Code	Label
3,696	99.9	0	Not a Bus
0	0.0	1	School
0	0.0	4	Intercity
0	0.0	5	Charter/Tour
0	0.0	6	Transit/ Commuter

N	Prcnt	Code	Label
0	0.0	7	Shuttle
3	0.1	8	Modified for Personal/Private Use
0	0.0	98	Not Reported
0	0.0	99	Unknown

Variable	Name	Format	Type	Length
v172	GVWR	GVWR10F	Numeric	8

GROSS VEHICLE WEIGHT RATING

N	Prcnt	Code	Label
217	5.9	0	Not Applicable
2	0.1	1	10,000 lbs. or less
526	14.2	2	10,001 lbs - 26,000 lbs
2,949	79.7	3	26,001 lbs. or more
1	0.0	8	Not Reported
4	0.1	9	Unknown

Variable	Name	Format	Type	Length
v179	HAZARDOUS MATERIAL CLASS NUMBER	HAZCN10F	Numeric	8

N	Prcnt	Code	Label
3,592	97.1	0	Not applicable
4	0.1	1	Explosives
17	0.5	2	Gases
51	1.4	3	Flammable liquid and combustible liquid
1	0.0	4	Flammable solid, spontaneously combustible, and dangerous when wet
0	0.0	5	Oxidizers and organic peroxide
2	0.1	6	Poison (toxic) and poison inhalation hazard
0	0.0	7	Radioactive
5	0.1	8	Corrosive
1	0.0	9	Miscellaneous
26	0.7	88	Not reported

Variable	Name	Format	Type	Length
v180	HAZARDOUS MATERIAL ID NUMBER	HAZID10F	Numeric	8

N	Prcnt	Code	Label
3,592	97.1	0	Not Applicable
1	0.0	3	
		-	Hazardous material identification number
1	0.0	3257	
25	0.7	8888	Not Reported

Variable	Name	Format	Type	Length
v181	HAZARDOUS MATERIAL INVOLVEMENT	HAZIN10F	Numeric	8

N	Prcnt	Code	Label
3,592	97.1	1	No
107	2.9	2	Yes

Variable	Name	Format	Type	Length
v182	HAZARDOUS MATERIAL PLACARD	HAZPL10F	Numeric	8

N	Prcnt	Code	Label
3,592	97.1	0	Not Applicable
4	0.1	1	No
98	2.6	2	Yes
5	0.1	8	Not Reported

Variable	Name	Format	Type	Length
v183	HAZARDOUS MATERIAL RELEASED	HAZRL10F	Numeric	8

N	Prcnt	Code	Label
3,592	97.1	0	Not Applicable
57	1.5	1	No
36	1.0	2	Yes
14	0.4	8	Not Reported

Variable	Name	Format	Type	Length
v184	MCID ISSUING AUTHORITY	MCISA10F	Numeric	8

N	Prcnt	Code	Label
219	5.9	0	Not Applicable
7	0.2	1	
		-	FARS state code
49	1.3	56	
2,451	66.3	57	US DOT
25	0.7	58	MC/MX (ICC)
202	5.5	77	Not Reported
266	7.2	88	None
2	0.1	95	Canada
195	5.3	99	Unknown

Variable	Name	Format	Type	Length
v185	MCID IDENTIFICATION NUMBER	$MCID10F	Char	9

N	Prcnt	Code	Label
1	0.0	165420	
		-	MCID identification number
1	0.0	tkd3112	
219	5.9	000000000	Not Applicable
202	5.5	777777777	Not Reported
266	7.2	888888888	None
194	5.2	999999999	Unknown

Variable	Name	Format	Type	Length
v186	HIT AND RUN	HIT10F	Numeric	8

N	Prcnt	Code	Label
3,659	98.9	0	No
36	1.0	1	Yes
1	0.0	9	Unknown

Variable	Name	Format	Type	Length
v190	PRE-EVENT MOVEMENT (PRIOR TO RECOGNITION OF CRITICAL EVENT	P1CRH10F	Numeric	8

N	Prcnt	Code	Label
42	1.1	0	No Driver Present
2,412	65.2	1	Going Straight
100	2.7	2	Decelerating in Traffic Lane
14	0.4	3	Accelerating in traffic lane
31	0.8	4	Starting in traffic lane
185	5.0	5	Stopped in traffic lane
31	0.8	6	Passing or Overtaking Another Vehicle
4	0.1	7	Disabled or "Parked" in Travel lane
4	0.1	8	Leaving a Parking Position
3	0.1	9	Entering a Parking Position
59	1.6	10	Turning Right
136	3.7	11	Turning Left
11	0.3	12	Making a U-turn
44	1.2	13	Backing Up (other than for Parking Position)
557	15.1	14	Negotiating a Curve
33	0.9	15	Changing Lanes
7	0.2	16	Merging
13	0.4	17	Successful Avoidance Maneuver to a Previous Critical Event
7	0.2	98	Other (specify:)
6	0.2	99	Unknown

Variable	Name	Format	Type	Length
v191	CRITICAL EVENT - PRECRASH (EVENT)	P2CRH10F	Numeric	8

N	Prcnt	Code	Label
21	0.6	1	Blow out/flat tire
1	0.0	2	Stalled engine
6	0.2	3	Disabling vehicle failure (e.g., wheel fell off) (specify:)
8	0.2	4	Non-disabling vehicle problem (e.g., hood flew up)(specify:)
15	0.4	5	Poor road conditions (puddle, pothole, ice, etc.) (Specify:)
52	1.4	6	Traveling too fast for conditions
31	0.8	8	Other cause of control loss (specify:)
10	0.3	9	Unknown cause of control loss
123	3.3	10	Over the lane line on left side of travel lane

N	Prcnt	Code	Label
76	2.1	11	Over the lane line on right side of travel lane
94	2.5	12	Off the edge of the road on the left side
174	4.7	13	Off the edge of the road on the right side
1	0.0	14	End departure
83	2.2	15	Turning left at intersection
16	0.4	16	Turning right at intersection
201	5.4	17	Crossing over (passing through) intersection
111	3.0	18	This vehicle decelerating
3	0.1	19	Unknown travel direction
141	3.8	50	Other vehicle stopped
86	2.3	51	Traveling in same direction with lower or steady speed
56	1.5	52	Traveling in same direction while decelerating
330	8.9	53	Traveling in same direction with higher speed
117	3.2	54	Traveling in opposite direction
33	0.9	55	In crossover
7	0.2	56	Backing
8	0.2	59	Unknown travel direction of the other motor vehicle in lane
104	2.8	60	From adjacent lane (same direction) over left lane line
87	2.4	61	From adjacent lane (same direction) over right lane line
810	21.9	62	From opposite direction over left lane line
21	0.6	63	From opposite direction over right lane line
62	1.7	64	From parking lane, median, shoulder, roadside
18	0.5	65	From crossing street, turning into same direction
279	7.5	66	From crossing street, across path
54	1.5	67	From crossing street, turning into opposite direction
9	0.2	68	From crossing street, intended path not known
1	0.0	70	From driveway, turning into same direction
17	0.5	71	From driveway, across path
12	0.3	72	From driveway, turning into opposite direction
6	0.2	73	From driveway, intended path not known
11	0.3	74	From entrance to limited access highway
18	0.5	78	Encroachment by other vehicle - details unknown
204	5.5	80	Pedestrian in roadway
12	0.3	81	Pedestrian approaching roadway
5	0.1	82	Pedestrian unknown location
50	1.4	83	Pedalcyclist or other non-motorist in roadway (specify)
6	0.2	84	Pedalcyclist or other non-motorist approaching roadway (specify)
4	0.1	87	Animal in roadway

N	Prcnt	Code	Label
1	0.0	88	Animal approaching roadway
14	0.4	90	Object in roadway
3	0.1	91	Object approaching roadway
81	2.2	98	Other critical precrash event (specify:)
6	0.2	99	Unknown

Variable	Name	Format	Type	Length
v192	ATTEMPTED AVOIDANCE MANEUVER	P3CRH10F	Numeric	8

N	Prcnt	Code	Label
42	1.1	0	No Driver Present
2,298	62.1	1	No Avoidance Maneuver
130	3.5	2	Braking (no lockup)
135	3.6	3	Braking (lockup)
113	3.1	4	Braking (lockup unknown)
2	0.1	5	Releasing brakes
202	5.5	6	Steering left
217	5.9	7	Steering right
146	3.9	8	Braking and steering left
179	4.8	9	Braking and steering right
5	0.1	10	Accelerating
2	0.1	11	Accelerating and steering left
1	0.0	12	Accelerating and steering right
12	0.3	98	Other actions (specify:)
215	5.8	99	Unknown

Variable	Name	Format	Type	Length
v193	PRE-IMPACT STABILITY	P4CRH10F	Numeric	8

N	Prcnt	Code	Label
42	1.1	0	No Driver Present
3,304	89.3	1	Tracking
226	6.1	2	Skidding longitudinally rotation less than 30 degrees
25	0.7	3	Skidding laterally clockwise rotation
53	1.4	4	Skidding laterally counterclockwise rotation
11	0.3	7	Other vehicle loss-of-control (specify:)
38	1.0	9	Precrash stability unknown

Variable	Name	Format	Type	Length
v194	PRE-IMPACT LOCATION	P5CRH10F	Numeric	8

N	Prcnt	Code	Label
42	1.1	0	No Driver Present
2,604	70.4	1	Stayed in original travel lane
507	13.7	2	Stayed on roadway, but left original travel lane
35	0.9	3	Stayed on roadway, not known if left original travel lane
435	11.8	4	Departed roadway
23	0.6	5	Remained off roadway
23	0.6	6	Returned to roadway
14	0.4	7	Entered roadway
16	0.4	9	Unknown

Variable	Name	Format	Type	Length
v195	CRASH TYPE	ACCTP10F	Numeric	8

N	Prcnt	Code	Label
21	0.6	0	No Impact
263	7.1	1	Drive Off Road
67	1.8	2	Control/Traction Loss
11	0.3	3	Avoid Collision With Vehicle., Pedestrian., Animal
222	6.0	4	Specifics Other
13	0.4	5	Specifics Unknown
7	0.2	11	Parked Vehicle
8	0.2	12	Stationary Object
270	7.3	13	Pedestrian/Animal
4	0.1	14	End Departure
80	2.2	20	Stopped
96	2.6	21	Stopped, Straight
14	0.4	22	Stopped, Left
91	2.5	24	Slower
151	4.1	25	Slower, Going Straight
7	0.2	26	Slower, Going Left
6	0.2	27	Slower, Going Right
27	0.7	28	Decelerating (Slowing)
31	0.8	29	Decelerating (Slowing), Going Straight
3	0.1	30	Decelerating (Slowing), Going Left

N	Prcnt	Code	Label
5	0.1	31	Decelerating (Slowing), Going Right
27	0.7	44	Straight Ahead on Left
100	2.7	45	Straight Ahead on Left/Right
10	0.3	46	Changing Lanes to the Right
4	0.1	47	Changing Lanes to the Left
141	3.8	50	Lateral Move (Left/Right)
707	19.1	51	Lateral Move (Going Straight)
42	1.1	68	Initial Opposite Directions (Left/Right)
82	2.2	69	Initial Opposite Directions (Going Straight)
2	0.1	70	Initial Same Directions (Turning Right)
19	0.5	71	Initial Same Directions (Going Straight)
10	0.3	72	Initial Same Directions (Turning Left)
2	0.1	76	Turn Into Same Direction (Turning Left)
16	0.4	77	Turn Into Same Direction (Going Straight)
1	0.0	78	Turn Into Same Direction (Turning Right)
4	0.1	80	Turn Into Opposite Directions (Turning Right)
96	2.6	81	Turn Into Opposite Directions (Going Straight)
42	1.1	82	Turn Into Opposite Directions (Turning Left)
133	3.6	86	Striking from the Right
63	1.7	87	Struck on the Right
166	4.5	88	Striking from the Left
63	1.7	89	Struck on the left
24	0.6	92	Backing Veh.
5	0.1	93	Other Vehicle or Object
542	14.7	98	Other Crash Type
1	0.0	99	Unknown

The DRIVER Variables

Variables 207 through 240 describe the driver of the truck involved in the crash.

Variable	Name	Format	Type	Length
v207	DRIVER PRESENCE	DRIPR10F	Numeric	8

N	Prcnt	Code	Label
39	1.1	0	No Driver Present/Not Applicable
3,660	98.9	1	Yes

Variable	Name	Format	Type	Length
v208	DRIVER DRINKING	DRIDR10F	Numeric	8

N	Prcnt	Code	Label
3,584	96.9	0	No
115	3.1	1	Yes

Variable	Name	Format	Type	Length
v209	DRIVER'S LICENSE STATE	LSTAT10F	Numeric	8

N	Prcnt	Code	Label
0	0.0	0	No Driver Present
107	2.9	1	Alabama
5	0.1	2	Alaska
50	1.4	4	Arizona
78	2.1	5	Arkansas
247	6.7	6	California
44	1.2	8	Colorado
14	0.4	9	Connecticut
13	0.4	10	Delaware
2	0.1	11	District of Columbia
209	5.7	12	Florida
170	4.6	13	Georgia
5	0.1	15	Hawaii
27	0.7	16	Idaho
124	3.4	17	Illinois
104	2.8	18	Indiana
81	2.2	19	Iowa

N	Prcnt	Code	Label
51	1.4	20	Kansas
95	2.6	21	Kentucky
80	2.2	22	Louisiana
18	0.5	23	Maine
27	0.7	24	Maryland
17	0.5	25	Massachusetts
91	2.5	26	Michigan
82	2.2	27	Minnesota
62	1.7	28	Mississippi
109	2.9	29	Missouri
9	0.2	30	Montana
38	1.0	31	Nebraska
15	0.4	32	Nevada
6	0.2	33	New Hampshire
55	1.5	34	New Jersey
19	0.5	35	New Mexico
113	3.1	36	New York
130	3.5	37	North Carolina
7	0.2	38	North Dakota
143	3.9	39	Ohio
78	2.1	40	Oklahoma
41	1.1	41	Oregon
151	4.1	42	Pennsylvania
4	0.1	44	Rhode Island
60	1.6	45	South Carolina
21	0.6	46	South Dakota
83	2.2	47	Tennessee
428	11.6	48	Texas
32	0.9	49	Utah
12	0.3	50	Vermont
67	1.8	51	Virginia
54	1.5	53	Washington
34	0.9	54	West Virginia
66	1.8	55	Wisconsin
18	0.5	56	Wyoming
35	0.9	95	Canada
13	0.4	96	Mexico
53	1.4	99	Unknown

Variable	Name	Format	Type	Length
v210A	NON-CDL LICENSE TYPE	CDLTP10F	Numeric	8

NON-COMMERCIAL DRIVER LICENSE TYPE, REGARDLESS OF VEHICLE DRIVEN

N	Prcnt	Code	Label
9	0.2	0	Not Licensed
3,595	97.2	1	Full Driver License
3	0.1	2	Intermediate Driver License
1	0.0	8	Temporary License
89	2.4	9	Unknown License Type

Variable	Name	Format	Type	Length
v210B	NON-CDL LICENSE STATUS	NCDLS10F	Numeric	8

NON-COMMERCIAL DRIVER LICENSE STATUS, REGARLESS OF VEHICLE DRIVEN

N	Prcnt	Code	Label
9	0.2	0	Not licensed
34	0.9	1	Suspended
3	0.1	2	Revoked
6	0.2	3	Expired
4	0.1	4	Canceled or denied
3,552	96.0	6	Valid
89	2.4	9	Unknown License Status

Variable	Name	Format	Type	Length
v211	LICENSE CLASS COMPLIANCE	LCOMP10F	Numeric	8

LICENSE CLASS COMPLIANCE (FOR THIS CLASS VEHICLE)

N	Prcnt	Code	Label
9	0.2	0	Not licensed
5	0.1	1	No license required for this class vehicle
103	2.8	2	No valid license for this class vehicle
3,474	93.9	3	Valid license for this class vehicle
1	0.0	7	Not Reported

N	Prcnt	Code	Label
8	0.2	8	Unknown If CDL and/or CDL endorsement required for this vehicle
97	2.6	9	Unknown

Variable	Name	Format	Type	Length
v212	CDL LICENSE STATUS	CMLIC10F	Numeric	8

COMMERCIAL MOTOR VEHICLE LICENSE STATUS

N	Prcnt	Code	Label
556	15.0	0	No (CDL)
19	0.5	1	Suspended
2	0.1	2	Revoked
23	0.6	3	Expired
3	0.1	4	Cancelled or Denied
12	0.3	5	Disqualified
2,973	80.4	6	Valid
4	0.1	7	Learner`s Permit
8	0.2	8	Other Not-Valid
1	0.0	98	Not Reported
96	2.6	99	Unknown

Variable	Name	Format	Type	Length
v213	COMPLIANCE WITH CDL ENDORSEMENTS	CLEND10F	Numeric	8

COMPLIANCE WITH COMMERCIAL DRIVER LICENSE ENDORSEMENTS

N	Prcnt	Code	Label
2,472	66.8	0	No Endorsements required for this vehicle
796	21.5	1	Endorsement(s) Required, Complied with
22	0.6	2	Endorsement(s) Required, Not Complied with
238	6.4	3	Endorsement(s) Required, Compliance Unknown
10	0.3	8	Not Reported
161	4.4	9	Unknown, if required

Variable	Name	Format	Type	Length
v214	LICENSE RESTRICTIONS MET	LREST10F	Numeric	8

N	Prcnt	Code	Label
2,571	69.5	0	No Restrictions or Not Applicable
263	7.1	1	Restrictions Complied With
19	0.5	2	Restrictions Not Complied With
739	20.0	3	Restrictions, Compliance Unknown
3	0.1	8	Not Reported
104	2.8	9	Unknown

Variable	Name	Format	Type	Length
v216	NUMBER OF PREV CRASHES	FNOPAC	Numeric	8

NUMBER OF PREVIOUS CRASHES

N	Prcnt	Code	Label
2,888	78.1	0	0 crashes
371	10.0	1	1 crashes
57	1.5	2	2 crashes
10	0.3	3	3 crashes
241	6.5	98	Crashes not reported on driving record
132	3.6	99	Unknown

Variable	Name	Format	Type	Length
v217	NUMBER PREV SUSPENSIONS	FNOPSUS	Numeric	8

NUMBER OF PREVIOUS SUSPENSIONS

N	Prcnt	Code	Label
3,284	88.8	0	0 suspensions
155	4.2	1	1 suspension
68	1.8	2	2 suspensions
24	0.6	3	3 suspensions
17	0.5	4	4 suspensions
7	0.2	5	5 suspensions
6	0.2	6	6 suspensions
3	0.1	7	7 suspensions
3	0.1	8	8 suspensions

N	Prcnt	Code	Label
1	0.0	9	9 suspensions
1	0.0	10	10 suspensions
1	0.0	18	18 suspensions
129	3.5	99	Unknown

Variable	Name	Format	Type	Length
v218	NUMBER OF PREV DWI CONV	FNODWI	Numeric	8

NUMBER OF PREVIOUS DRIVING WHILE INTOXICATED CONVICTIONS

N	Prcnt	Code	Label
3,545	95.8	0	0 driving while intoxicated convictions
19	0.5	1	1 driving while intoxicated conviction
6	0.2	2	2 driving while intoxicated convictions
129	3.6	99	Unknown

Variable	Name	Format	Type	Length
v219	NUM PREV SPEEDING CONV	FNOPSPD	Numeric	8

NUMBER OF PREVIOUS SPEEDING CONVICTIONS

N	Prcnt	Code	Label
2,826	76.4	0	0 speed convictions
551	14.9	1	1 speed conviction
148	4.0	2	2 speed convictions
33	0.9	3	3 speed convictions
10	0.3	4	4 speed convictions
1	0.0	6	6 speed convictions
1	0.0	9	9 speed convictions
129	3.5	99	Unknown

Variable	Name	Format	Type	Length
v220	NUM PREV OTHER MV CONV	FNOPOTH	Numeric	8

NUMBER OF PREVIOUS OTHER HARMFUL MOVING VIOLATION CONVICTIONS

N	Prcnt	Code	Label
2,716	73.4	0	0 other convictions

N	Prcnt	Code	Label
592	16.0	1	1 other conviction
150	4.1	2	2 other convictions
57	1.5	3	3 other convictions
26	0.7	4	4 other convictions
21	0.6	5	5 other convictions
3	0.1	6	6 other convictions
4	0.1	7	7 other convictions
1	0.0	9	9 other convictions
129	3.5	99	Unknown

Variable	Name	Format	Type	Length
v221	LAST CRASH - MONTH	FLMON10F	Numeric	8

LAST CRASH/SUSPENSIONS/CONVICTION – MONTH

N	Prcnt	Code	Label
1,931	52.2	0	No Record
109	2.9	1	January
129	3.5	2	February
152	4.1	3	March
133	3.6	4	April
141	3.8	5	May
136	3.7	6	June
143	3.9	7	July
145	3.9	8	August
156	4.2	9	September
132	3.6	10	October
122	3.3	11	November
140	3.8	12	December
130	3.5	99	Unknown

Variable	Name	Format	Type	Length
v222	LAST CRASH - YEAR	6	Numeric	8

LAST CRASH/SUSPENSION/CONVICTION – YEAR

N	Prcnt	Code	Label
1,931	52.2	0	No record

N	Prcnt	Code	Label
163	4.4	2007	2007
438	11.8	2008	2008
589	15.9	2009	2009
448	12.1	2010	2010
130	3.5	9999	Unknown

Variable	Name	Format	Type	Length
v223	FIRST CRASH - MONTH	FLMON10F	Numeric	8

FIRST CRASH/SUSPENSION/CONVICTION – MONTH

N	Prcnt	Code	Label
1,931	52.2	0	No Record
112	3.0	1	January
112	3.0	2	February
155	4.2	3	March
146	3.9	4	April
134	3.6	5	May
154	4.2	6	June
131	3.5	7	July
159	4.3	8	August
146	3.9	9	September
149	4.0	10	October
119	3.2	11	November
121	3.3	12	December
130	3.5	99	Unknown

Variable	Name	Format	Type	Length
v224	FIRST CRASH - YEAR	6	Numeric	8

FIRST CRASH/SUSPENSION/CONVICTION – YEAR

N	Prcnt	Code	Label
1,931	52.2	0	No record
395	10.7	2007	2007
656	17.7	2008	2008
403	10.9	2009	2009

N	Prcnt	Code	Label
184	5.0	2010	2010
130	3.5	9999	Unknown

Variable	Name	Format	Type	Length
v227	DRIVER RELATED FACTORS #1	DRF10F	Numeric	8

FACTORS AT DRIVER LEVEL – RESPONSE #1

N	Prcnt	Code	Label
2,692	72.8	0	None
1	0.0	4	Reaction to or Failure to Take Drugs/Medication
2	0.1	8	Aggressive Driving / Road Rage
0	0.0	12	Mother of Dead Fetus
0	0.0	13	Mentally Challenged
0	0.0	15	Seat Back Not In Normal Upright Position, Seat Back Reclined
0	0.0	16	Police or Law Enforcement Officer
1	0.0	18	Traveling on Prohibited Trafficways
0	0.0	19	Legally Driving on Suspended or Revoked License
20	0.5	20	Leaving Vehicle Unattended with Engine Running. Leaving Vehicle Unattended in Roadway
10	0.3	21	Overloading or Improper Loading of Vehicle with Passengers or Cargo
3	0.1	22	Towing or Pushing Vehicle Improperly
0	0.0	23	Failing to Dim Lights or to Have Lights on When Required
19	0.5	24	Operating without Required Equipment
71	1.9	26	Following Improperly
10	0.3	27	Improper or Erratic Lane Changing
222	6.0	28	Failure to keep in Proper Lane
6	0.2	29	Illegal Driving on Road Shoulder, in Ditch, on Sidewalk or on Median
3	0.1	30	Making Improper Entry to or Exit from Trafficway
19	0.5	31	Starting or Backing Improperly
0	0.0	32	Opening Closure into Moving Traffic or While Vehicle is in motion
3	0.1	33	Passing where Prohibited by Posted Signs, Pavement Markings, Hill or Curve, or School Bus Displaying Warning not to Pass Line
1	0.0	34	Passing on Wrong Side
4	0.1	35	Passing with Insufficient Distance or Inadequate Visibility or Failing to Yield to Overtaking Vehicle

N	Prcnt	Code	Label
58	1.6	36	Operating the Vehicle in an Erratic, Reckless or Negligent Manner, Operating at Erratic or Suddenly Changing Speeds.
0	0.0	37	Police Pursuing this Driver or Police Officer in Pursuit
145	3.9	38	Failure to Yield Right-of-Way
54	1.5	39	Failure to Obey Actual Traffic Sign, Traffic Control Devices or Traffic Officers; Failure to Obey Safety Zone Traffic Laws
0	0.0	40	Passing Through or Around Barrier
2	0.1	41	Failure to Observe Warnings or Instructions on Vehicles Displaying Them
1	0.0	42	Failure to Signal Intentions
4	0.1	45	Driving Less than Posted Minimum
1	0.0	47	Making Right Turn From Left-Turn Lane. Make Left Turn From Right-Turn Lane
21	0.6	48	Making Other Improper Turn
0	0.0	50	Driving Wrong Way on One-Way Traffic
37	1.0	51	Driving on Wrong Side of Road (Intentional or Unintentional)
1	0.0	52	Operator Inexperience
0	0.0	53	Unfamiliar with Roadway
24	0.6	54	Stopped in Roadway (Vehicle Not Abandoned)
2	0.1	57	Locked Wheel
28	0.8	58	Overcorrecting
2	0.1	59	Getting Off/Out of or On/In to a Vehicle
0	0.0	73	Driver has not complied with Learners Permit or Intermediate Driver License Restrictions (GDL Restrictions)
15	0.4	74	Driver Has Not Complied With Physical or Other Imposed Restrictions (not including GDL Restrictions)
1	0.0	77	Severe Crosswind
0	0.0	78	Wind from Passing Truck
3	0.1	79	Slippery or Loose Surface
12	0.3	80	Tire Blowout or Flat
0	0.0	81	Debris or Objects in Road
0	0.0	82	Ruts, Holes, Bumps in Road
5	0.1	83	Live Animals in Road
21	0.6	84	Vehicle in Road
0	0.0	85	Phantom Vehicle
18	0.5	86	Pedestrian, Pedal Cyclist or Other Non-Motorist
27	0.7	87	Ice, Snow, Slush, Water, Sand, Dirt, Oil, Wet Leaves on Road
1	0.0	88	Trailer Fishtailing or swaying
11	0.3	89	Driver Has a Driving Record or Drivers License From More Than One State

N	Prcnt	Code	Label
12	0.3	91	Non-Traffic Violation Charged - manslaughter, homicide or other assault committed without malice.
54	1.5	92	Other Non-Moving Traffic Violations
52	1.4	99	Unknown

Variable	Name	Format	Type	Length
v228	DRIVER RELATED FACTORS #2	DRF10F	Numeric	8

FACTORS AT DRIVER LEVEL – RESPONSE #2

N	Prcnt	Code	Label
3,473	93.9	0	None
1	0.0	21	Overloading or Improper Loading of Vehicle with Passengers or Cargo
1	0.0	23	Failing to Dim Lights or to Have Lights on When Required
2	0.1	24	Operating without Required Equipment
6	0.2	26	Following Improperly
1	0.0	27	Improper or Erratic Lane Changing
12	0.3	28	Failure to keep in Proper Lane
1	0.0	29	Illegal Driving on Road Shoulder, in Ditch, on Sidewalk or on Median
1	0.0	30	Making Improper Entry to or Exit from Trafficway
2	0.1	31	Starting or Backing Improperly
1	0.0	35	Passing with Insufficient Distance or Inadequate Visibility or Failing to Yield to Overtaking Vehicle
14	0.4	36	Operating the Vehicle in an Erratic, Reckless or Negligent Manner, Operating at Erratic or Suddenly Changing Speeds.
1	0.0	37	Police Pursuing this Driver or Police Officer in Pursuit
25	0.7	39	Failure to Obey Actual Traffic Sign, Traffic Control Devices or Traffic Officers;Failure to Obey Safety Zone Traffic Laws
1	0.0	41	Failure to Observe Warnings or Instructions on Vehicles Displaying Them
6	0.2	48	Making Other Improper Turn
7	0.2	51	Driving on Wrong Side of Road (Intentional or Unintentional)
1	0.0	52	Operator Inexperience
2	0.1	53	Unfamiliar with Roadway
1	0.0	54	Stopped in Roadway (Vehicle Not Abandoned)
1	0.0	57	Locked Wheel
26	0.7	58	Overcorrecting
1	0.0	73	Driver has not complied with Learners Permit or Intermediate Driver License Restrictions (GDL Restrictions)

N	Prcnt	Code	Label
1	0.0	74	Driver Has Not Complied With Physical or Other Imposed Restrictions (not including GDL Restrictions)
2	0.1	80	Tire Blowout or Flat
1	0.0	81	Debris or Objects in Road
4	0.1	84	Vehicle in Road
1	0.0	85	Phantom Vehicle
2	0.1	86	Pedestrian, Pedal Cyclist or Other Non-Motorist
12	0.3	87	Ice, Snow, Slush, Water, Sand, Dirt, Oil, Wet Leaves on Road
4	0.1	89	Driver Has a Driving Record or Drivers License From More Than One State
11	0.3	91	Non-Traffic Violation Charged - manslaughter, homicide or other assault committed without malice.
11	0.3	92	Other Non-Moving Traffic Violations
52	1.4	99	Unknown

Variable	Name	Format	Type	Length
v229	DRIVER RELATED FACTORS #3	DRF10F	Numeric	8

FACTORS AT DRIVER LEVEL – RESPONSE #3

N	Prcnt	Code	Label
3,617	97.8	0	None
2	0.1	28	Failure to keep in Proper Lane
1	0.0	29	Illegal Driving on Road Shoulder, in Ditch, on Sidewalk or on Median
1	0.0	30	Making Improper Entry to or Exit from Trafficway
1	0.0	35	Passing with Insufficient Distance or Inadequate Visibility or Failing to Yield to Overtaking Vehicle
1	0.0	38	Failure to Yield Right-of-Way
1	0.0	39	Failure to Obey Actual Traffic Sign, Traffic Control Devices or Traffic Officers; Failure to Obey Safety Zone Traffic Laws
1	0.0	53	Unfamiliar with Roadway
1	0.0	54	Stopped in Roadway (Vehicle Not Abandoned)
1	0.0	57	Locked Wheel
1	0.0	58	Overcorrecting
2	0.1	74	Driver Has Not Complied With Physical or Other Imposed Restrictions (not including GDL Restrictions)
1	0.0	77	Severe Crosswind
1	0.0	80	Tire Blowout or Flat
2	0.1	87	Ice, Snow, Slush, Water, Sand, Dirt, Oil, Wet Leaves on Road

N	Prcnt	Code	Label
7	0.2	91	Non-Traffic Violation Charged - manslaughter, homicide or other assault committed without malice.
6	0.2	92	Other Non-Moving Traffic Violations
52	1.4	99	Unknown

Variable	Name	Format	Type	Length
v230	DRIVER RELATED FACTORS #4	DRF10F	Numeric	8

FACTORS AT DRIVER LEVEL – RESPONSE #4

N	Prcnt	Code	Label
3,642	98.5	0	None
1	0.0	29	Illegal Driving on Road Shoulder, in Ditch, on Sidewalk or on Median
1	0.0	51	Driving on Wrong Side of Road (Intentional or Unintentional)
1	0.0	86	Pedestrian, Pedal Cyclist or Other Non-Motorist
1	0.0	87	Ice, Snow, Slush, Water, Sand, Dirt, Oil, Wet Leaves on Road
1	0.0	92	Other Non-Moving Traffic Violations
52	1.4	99	Unknown

Variable	Name	Format	Type	Length
v235	DRIVER HEIGHT (INCHES)	11	Numeric	8

N	Prcnt	Code	Label
1	0.0	52	
		-	Actual inches
1	0.0	89	
544	14.7	99	Unknown

Variable	Name	Format	Type	Length
v236	DRIVER WEIGHT	6	Numeric	8

N	Prcnt	Code	Label
1	0.0	60	
		-	Actual weight in pounds
1	0.0	430	
1,247	33.7	999	Unknown

Variable	Name	Format	Type	Length
v240	SPEED RELATED	SPDRL10F	Numeric	8

N	Prcnt	Code	Label
37	1.0	.	.
3,319	89.7	0	No
314	8.5	1	Yes
27	0.7	9	Unknown

The OCCUPANT Variables

Variables 306 through 363 describe the occupant of the truck (in this case, the driver) and are obtained from the FARS occupant file.

Variable	Name	Format	Type	Length
v306	OCCUPANT NUMBER	6	Numeric	8

N	Prcnt	Code	Label
39	1.1	0	0
3,660	98.9	1	Occupant #1

Variable	Name	Format	Type	Length
v308	OCCUPANT AGE	FOCCAGE	Numeric	8

N	Prcnt	Code	Label
3	0.1	16	16 years
1	0.0	17	17 years
3	0.1	18	18 years
10	0.3	19	19 years
14	0.4	20	20 years
21	0.6	21	21 years
14	0.4	22	22 years
30	0.8	23	23 years
37	1.0	24	24 years
32	0.9	25	25 years
37	1.0	26	26 years
56	1.5	27	27 years
58	1.6	28	28 years
68	1.8	29	29 years
59	1.6	30	30 years
66	1.8	31	31 years
79	2.1	32	32 years
79	2.1	33	33 years
69	1.9	34	34 years
71	1.9	35	35 years
81	2.2	36	36 years
93	2.5	37	37 years
82	2.2	38	38 years
93	2.5	39	39 years

N	Prcnt	Code	Label
92	2.5	40	40 years
91	2.5	41	41 years
94	2.5	42	42 years
96	2.6	43	43 years
102	2.8	44	44 years
120	3.2	45	45 years
108	2.9	46	46 years
116	3.1	47	47 years
126	3.4	48	48 years
115	3.1	49	49 years
102	2.8	50	50 years
114	3.1	51	51 years
109	2.9	52	52 years
106	2.9	53	53 years
107	2.9	54	54 years
79	2.1	55	55 years
84	2.3	56	56 years
100	2.7	57	57 years
85	2.3	58	58 years
94	2.5	59	59 years
62	1.7	60	60 years
59	1.6	61	61 years
56	1.5	62	62 years
49	1.3	63	63 years
27	0.7	64	64 years
38	1.0	65	65 years
28	0.8	66	66 years
23	0.6	67	67 years
21	0.6	68	68 years
15	0.4	69	69 years
12	0.3	70	70 years
11	0.3	71	71 years
6	0.2	72	72 years
8	0.2	73	73 years
5	0.1	74	74 years
5	0.1	75	75 years
3	0.1	76	76 years
3	0.1	77	77 years
8	0.2	78	78 years

N	Prcnt	Code	Label
1	0.0	80	80 years
1	0.0	81	81 years
3	0.1	83	83 years
1	0.0	84	84 years
2	0.1	86	86 years
56	1.5	999	Unknown

Variable	Name	Format	Type	Length
v309	OCCUPANT SEX	SEX10F	Numeric	8

N	Prcnt	Code	Label
3,540	95.7	1	Male
108	2.9	2	Female
51	1.4	9	Unknown

Variable	Name	Format	Type	Length
v310	OCCUPANT TYPE	PTYPE10F	Numeric	8

N	Prcnt	Code	Label
3,660	98.9	1	Driver of a Motor Vehicle In-Transport
39	1.1	99	Unknown person type

Variable	Name	Format	Type	Length
v311	OCC SEATING POSITION	SEATP10F	Numeric	8

OCCUPANT SEATING POSITION

N	Prcnt	Code	Label
3,658	98.9	11	Front Seat, Left Side
1	0.0	13	Front Seat, Right Side
1	0.0	18	Front Seat, Other
39	1.1	99	Unknown

Variable	Name	Format	Type	Length
v312	RESTRAINT SYSTEM/HELMET USE	RHUSE10F	Numeric	8

N	Prcnt	Code	Label
1	0.0	0	Not Applicable
11	0.3	1	Shoulder Belt Only Used
105	2.8	2	Lap Belt Only Used
2,869	77.6	3	Shoulder and Lap Belt Used
411	11.1	7	None Used-Motor Vehicle Occupant
1	0.0	8	Restraint Used - Type Unknown
7	0.2	98	Not Reported
294	7.9	99	Unknown

Variable	Name	Format	Type	Length
v313	AIR BAG AVAIL/FUNCTION	AIRBG10F	Numeric	8

AIR BAG AVAILABLE/FUNCTIONING

N	Prcnt	Code	Label
2,412	65.2	0	Not Applicable
163	4.4	1	Deployed- Front
2	0.1	2	Deployed- Side (door, seatback)
3	0.1	3	Deployed- Curtain (roof)
6	0.2	7	Deployed- Other (Knee, air belt, etc.)
11	0.3	8	Deployed- Combination
60	1.6	9	Deployment- Unknown Location
919	24.8	20	Not Deployed
1	0.0	28	Switched Off
40	1.1	98	Not Reported
82	2.2	99	Deployment Unknown

Variable	Name	Format	Type	Length
v314	OCCUPANT EJECTION	EJECT10F	Numeric	8

N	Prcnt	Code	Label
3,507	94.8	0	Not Ejected
99	2.7	1	Totally Ejected
42	1.1	2	Partially Ejected
1	0.0	3	Ejected - Unknown Degree

N	Prcnt	Code	Label
1	0.0	8	Not Applicable
49	1.3	9	Unknown if Ejected

Variable	Name	Format	Type	Length
v315	EJECTION PATH	EPATH10F	Numeric	8

N	Prcnt	Code	Label
3,508	94.8	0	Not Ejected/Not Applicable
5	0.1	1	Through Side Door Opening
19	0.5	2	Through Side Window
12	0.3	3	Through Windshield
1	0.0	5	Through Back Door/Tailgate Opening
1	0.0	6	Through Roof Opening (sun-roof, convertible top down)
4	0.1	8	Other Path (e.g., back of pick-up truck)
149	4.0	9	Unknown/Unknown Path

Variable	Name	Format	Type	Length
v316	OCCUPANT EXTRICATION	EXTR10F	Numeric	8

N	Prcnt	Code	Label
3,438	92.9	0	Not Extricated or Not Applicable
202	5.5	1	Extricated
59	1.6	9	Unknown

Variable	Name	Format	Type	Length
v317	ANY INDICATION OF MIS-USE OF RESTRAINT SYSTEM/HELMET USE	RSTMU10F	Numeric	8

N	Prcnt	Code	Label
39	1.1	.	.
3,659	98.9	0	No
1	0.0	1	Yes

Variable	Name	Format	Type	Length
v320	DRUG STATUS	DRGST10F	Numeric	8

N	Prcnt	Code	Label
2,250	60.8	0	Test Not Given
1	0.0	1	Test Refused
1,159	31.3	2	Test Given
66	1.8	8	Not Reported
223	6.0	9	Unknown if Tested

Variable	Name	Format	Type	Length
v321	DRUG INVOLVEMENT	DRUG10F	Numeric	8

POLICE REPORTED DRUG INVOLVEMENT

N	Prcnt	Code	Label
1,589	43.0	0	No (drugs not involved)
60	1.6	1	Yes (drugs involved)
1,801	48.7	8	Not Reported
249	6.7	9	Unknown

Variable	Name	Format	Type	Length
v322	DRUG DETERMINATION	DRGDT10F	Numeric	8

METHOD OF DRUG DETERMINATION BY POLICE

N	Prcnt	Code	Label
272	7.4	1	Evidential Test (Blood, Urine)
15	0.4	2	Drug Recognition Technician (DRT) determination
26	0.7	3	Behavioral
333	9.0	7	Other
3,053	82.5	8	Not Reported

Variable	Name	Format	Type	Length
v323	DRUG TEST TYPE #1	DRGTT10F	Numeric	8

N	Prcnt	Code	Label
2,251	60.9	0	Test Not Given
976	26.4	1	Blood

N	Prcnt	Code	Label
131	3.5	2	Urine
33	0.9	3	Both: Blood and Urine Tests
66	1.8	6	Not Reported
12	0.3	7	Unknown Test Type
7	0.2	8	Other Test Type
223	6.0	9	Unknown if Tested

Variable	Name	Format	Type	Length
v324	DRUG TEST RESULTS #1	DRGRS10F	Numeric	8

N	Prcnt	Code	Label
2,251	60.9	0	Test Not Given
829	22.4	1	No Drugs Reported / Negative
87	2.4	95	Not Reported
0	0.0	100	
		-	Narcotic drug
2	0.1	295	
4	0.1	300	
		-	Depressant drug
1	0.0	395	
0	0.0	400	
		-	Stimulant drug
6	0.2	495	
0	0.0	500	
		-	Hallucinogen drug
0	0.0	595	
2	0.1	600	
		-	Cannabinoid drug
6	0.2	695	
0	0.0	700	
		-	Phencyclidine (PCP)
0	0.0	795	

N	Prcnt	Code	Label
0	0.0	800	
		-	Anabolic steroid
0	0.0	895	
0	0.0	900	
		-	Inhalant drug
0	0.0	995	
36	1.0	996	Other Drug
157	4.2	997	Test For Drug, Results Unknown
6	0.2	998	Tested For Drugs, Drugs Found, Type unknown/Positive
223	6.0	999	Unknown if Tested

Variable	Name	Format	Type	Length
v325	DRUG TEST TYPE #2	DRGTT10F	Numeric	8

N	Prcnt	Code	Label
3,339	90.3	0	Test Not Given
87	2.4	1	Blood
15	0.4	2	Urine
5	0.1	3	Both: Blood and Urine Tests
30	0.8	6	Not Reported
223	6.0	9	Unknown if Tested

Variable	Name	Format	Type	Length
v326	DRUG TEST RESULTS #2	DRGRS10F	Numeric	8

N	Prcnt	Code	Label
3,338	90.2	0	Test Not Given
50	1.4	1	No Drugs Reported / Negative
30	0.8	95	Not Reported
0	0.0	100	
		-	Narcotic drug
0	0.0	295	

N	Prcnt	Code	Label
0	0.0	300	
		-	Depressant drug
1	0.0	395	
0	0.0	400	
		-	Stimulant drug
0	0.0	495	
2	0.1	600	
		-	Cannabinoid drug
2	0.1	695	
14	0.4	996	Other Drug
1	0.0	997	Test For Drug, Results Unknown
1	0.0	998	Tested For Drugs, Drugs Found, Type unknown/Positive
223	6.0	999	Unknown if Tested

Variable	Name	Format	Type	Length
v327	DRUG TEST TYPE #3	DRGTT10F	Numeric	8

N	Prcnt	Code	Label
3,377	91.3	0	Test Not Given
59	1.6	1	Blood
7	0.2	2	Urine
3	0.1	3	Both: Blood and Urine Tests
30	0.8	6	Not Reported
223	6.0	9	Unknown if Tested

Variable	Name	Format	Type	Length
v328	DRUG TEST RESULTS #3	DRGRS10F	Numeric	8

N	Prcnt	Code	Label
3,376	91.3	0	Test Not Given
48	1.3	1	No Drugs Reported / Negative
30	0.8	95	Not Reported

N	Prcnt	Code	Label
0	0.0	100	
		-	Narcotic drug
0	0.0	295	
2	0.1	300	
		-	Depressant drug
0	0.0	295	
0	0.0	400	
		-	Stimulant drug
0	0.0	495	
0	0.0	600	
		-	Cannabinoid drug
1	0.0	695	
6	0.2	996	Other Drug
1	0.0	998	Tested For Drugs, Drugs Found, Type unknown/Positive
223	6.0	999	Unknown if Tested

Variable	Name	Format	Type	Length
v329	OCCUPANT INJURY SEVERITY	INJUR10F	Numeric	8

N	Prcnt	Code	Label
2,192	59.3	0	No Injury (O)
344	9.3	1	Possible Injury (C)
435	11.8	2	Non-incapacitating Evident Injury (B)
132	3.6	3	Incapacitating Injury (A)
540	14.6	4	Fatal Injury (K)
9	0.2	5	Injured, Severity Unknown
1	0.0	6	Died Prior to Crash*
46	1.2	9	Unknown

Variable	Name	Format	Type	Length
v330	TRANSPORTED TO MEDICAL FACILITY BY	THOSP10F	Numeric	8

N	Prcnt	Code	Label
2,828	76.5	0	Not Transported
128	3.5	1	EMS Air
13	0.4	2	Law Enforcement
53	1.4	3	EMS Unknown Mode
8	0.2	4	Transported Unknown Source
579	15.7	5	EMS Ground
18	0.5	6	Other
6	0.2	8	Not Reported
66	1.8	9	Unknown

Variable	Name	Format	Type	Length
v331	OCC DEATH DATE - MONTH	FACMONTH	Numeric	8

OCCUPANT DEATH DATE – MONTH

N	Prcnt	Code	Label
38	1.0	1	January
30	0.8	2	February
33	0.9	3	March
37	1.0	4	April
40	1.1	5	May
55	1.5	6	June
45	1.2	7	July
76	2.1	8	August
49	1.3	9	September
45	1.2	10	October
42	1.1	11	November
44	1.2	12	December
3,120	84.3	88	Not applicable (non-fatal)
45	1.2	99	99

Variable	Name	Format	Type	Length
v332	OCC DEATH DATE - DAY	6	Numeric	8

OCCUPANT DEATH DATE – DAY

N	Prcnt	Code	Label
18	0.5	1	
		-	Day of month
10	0.3	31	
3,120	84.3	88	Not applicable (non-fatal)
45	1.2	99	Unknown

Variable	Name	Format	Type	Length
v333	OCC DEATH DATE - YEAR	6	Numeric	8

OCCUPANT DEATH DATE – YEAR

N	Prcnt	Code	Label
537	14.5	2010	2010
3,120	84.3	8888	Not applicable (non-fatal)
42	1.1	9999	Unknown

Variable	Name	Format	Type	Length
v334	OCC DEATH TIME - HOURS	FACHOUR	Numeric	8

OCCUPANT DEATH TIME – HOURS

N	Prcnt	Code	Label
13	0.4	0	12:00 am - 12:59 am
19	0.5	1	1:00 am - 1:59 am
17	0.5	2	2:00 am - 2:59 am
20	0.5	3	3:00 am - 3:59 am
26	0.7	4	4:00 am - 4:59 am
19	0.5	5	5:00 am - 5:59 am
21	0.6	6	6:00 am - 6:59 am
21	0.6	7	7:00 am - 7:59 am
27	0.7	8	8:00 am - 8:59 am
15	0.4	9	9:00 am - 9:59 am
29	0.8	10	10:00 am - 10:59 am
29	0.8	11	11:00 am - 11:59 am

N	Prcnt	Code	Label
27	0.7	12	12:00 pm - 12:59 pm
27	0.7	13	1:00 pm - 1:59 pm
31	0.8	14	2:00 pm - 2:59 pm
22	0.6	15	3:00 pm - 3:59 pm
26	0.7	16	4:00 pm - 4:59 pm
20	0.5	17	5:00 pm - 5:59 pm
22	0.6	18	6:00 pm - 6:59 pm
11	0.3	19	7:00 pm - 7:59 pm
18	0.5	20	8:00 pm - 8:59 pm
14	0.4	21	9:00 pm - 9:59 pm
14	0.4	22	10:00 pm - 10:59 pm
17	0.5	23	11:00 pm - 11:59 pm
3,120	84.3	88	Not applicable (non-fatal)
74	2.0	99	Unknown

Variable	Name	Format	Type	Length
v335	OCC DEATH TIME - MINUTES	6	Numeric	8

OCCUPANT DEATH TIME -- MINUTES

N	Prcnt	Code	Label
28	0.8	0	
		-	Minute
7	0.2	59	
3,120	84.3	88	Not applicable (non-fatal)
74	2.0	99	Unknown

Variable	Name	Format	Type	Length
v336	LAG TIME CRASH/DEATH - HRS	11	Numeric	8

LAG TIME BETWEEN CRASH AND DEATH -- HOURS

N	Prcnt	Code	Label
337	9.1	0	
		-	Actual time in hours
1	0.0	674	
3,194	86.3	999	Unknown

Variable	Name	Format	Type	Length
v342	OCC FATAL INJURY AT WORK	WKINJ10F	Numeric	8

OCCUPANT FATAL INJURY AT WORK

N	Prcnt	Code	Label
76	2.1	0	No
389	10.5	1	Yes
3,120	84.3	8	Not Applicable (not a fatality)
114	3.1	9	Unknown

Variable	Name	Format	Type	Length
v345	OCC ALCOHOL INVOLVEMENT	DRINK10F	Numeric	8

OCCUPANT ALCOHOL INVOLVEMENT

N	Prcnt	Code	Label
2,532	68.5	0	No (Alcohol Not Involved)
81	2.2	1	Yes (Alcohol Involved)
793	21.4	8	Not Reported
293	7.9	9	Unknown (Police Reported)

Variable	Name	Format	Type	Length
v346	OCC METH ALC DETERMINAT	ALC_D10F	Numeric	8

METHOD OF ALCOHOL DETERMINATION BY POLICE

N	Prcnt	Code	Label
454	12.3	1	Evidential Test (breath, blood, urine)
117	3.2	2	Preliminary Breath Test (PBT)
17	0.5	3	Behavioral
3	0.1	4	Passive Alcohol Sensor (PAS)
664	18.0	5	Observed
33	0.9	8	Other (e.g., Saliva test)
2,411	65.2	9	Not Reported

Variable	Name	Format	Type	Length
v347	ALCOHOL TEST	ALCTT10F	Numeric	8

N	Prcnt	Code	Label
1,863	50.4	0	Test Not Given
1,262	34.1	1	Blood
96	2.6	2	Breathalyzer "BAC"
52	1.4	3	Urine
1	0.0	4	Vitreous
2	0.1	5	Blood Plasma/Serum
1	0.0	7	Liver
6	0.2	8	Other Test Type
127	3.4	10	Preliminary Breath Test (PBT)
59	1.6	95	Not Reported
13	0.4	98	Unknown Test Type
217	5.9	99	Unknown if Tested

Variable	Name	Format	Type	Length
v348	OCC ALCOHOL TEST RESULT	ALCRS10F	Numeric	8

OCCUPANT ALCOHOL TEST RESULT

N	Prcnt	Code	Label
1,356	36.7	0	
		-	Result value (grams/100 ml)%
0	0.0	80	
0	0.0	94	0.94 % or Greater
97	2.6	95	Not Reported
1,863	50.4	96	Test Not Given
83	2.2	97	AC Test Performed, Results Unknown
217	5.9	99	Unknown if tested

Variable	Name	Format	Type	Length
v349	ALCOHOL TEST STATUS	ALCST10F	Numeric	8

N	Prcnt	Code	Label
1,859	50.3	0	Test Not Given
4	0.1	1	Test Refused
1,560	42.2	2	Test Given

N	Prcnt	Code	Label
59	1.6	8	Not Reported
217	5.9	9	Unknown if Tested

Variable	Name	Format	Type	Length
v361	RACE	RACE10F	Numeric	8

N	Prcnt	Code	Label
3,119	84.3	0	Not a Fatality (not Applicable)
393	10.6	1	White
66	1.8	2	Black
1	0.0	3	American Indian (includes Aleuts and Eskimos)
2	0.1	4	Chinese
1	0.0	5	Japanese
2	0.1	18	Asian Indian
1	0.0	19	Other Indian (includes South and Central America, any other, except American or Asian Indians)
1	0.0	68	Other Asian or Pacific Islander
4	0.1	98	All other races
109	2.9	99	Unknown

Variable	Name	Format	Type	Length
v362	HISPANIC ORIGIN	HISPO10F	Numeric	8

N	Prcnt	Code	Label
3,120	84.3	0	Not A Fatality (not Applicable)
10	0.3	1	Mexican
1	0.0	2	Puerto Rican
3	0.1	4	Central or South American
1	0.0	5	European Spanish
22	0.6	6	Hispanic, Origin Not Specified or Other Origin
363	9.8	7	Non-Hispanic
179	4.8	99	Unknown

Variable	Name	Format	Type	Length
v363	OCC DEAD ON ARRIVAL	DOA10F	Numeric	8

OCCUPANT DEAD ON ARRIVAL

N	Prcnt	Code	Label
3,237	87.5	0	Not Applicable
415	11.2	7	Died at Scene
6	0.2	8	Died En Route
41	1.1	9	Unknown

The SURVEY Variables

Information in variables 1001 through 1062 and 1091 through 1126 was collected by the TIFA interview.

Variable	Name	Format	Type	Length
v1001	POWER UNIT MAKE	V1001_F	Numeric	4

N	Prcnt	Code	Label
8	0.2	1	Autocar
85	2.3	2	Chevrolet
118	3.2	3	Dodge
345	9.3	4	Ford
877	23.7	5	Freightliner
78	2.1	6	GMC
527	14.2	7	International
32	0.9	8	Isuzu
418	11.3	9	Kenworth
259	7.0	10	Mack
5	0.1	12	Mitsubishi Fuso
15	0.4	13	Nissan/UD
446	12.1	14	Peterbilt
243	6.6	15	Volvo
54	1.5	16	Western Star
5	0.1	17	White
21	0.6	18	WhiteGMC
80	2.2	19	Sterling
33	0.9	97	Other
50	1.4	99	Unknown

Variable	Name	Format	Type	Length
v1002	POWER UNIT YEAR	V1002_F	Numeric	4

N	Prcnt	Code	Label
1	0.0	1959	1959
1	0.0	1963	1963
1	0.0	1971	1971
1	0.0	1974	1974
2	0.1	1975	1975
3	0.1	1976	1976
3	0.1	1977	1977

N	Prcnt	Code	Label
2	0.1	1978	1978
4	0.1	1979	1979
8	0.2	1981	1981
6	0.2	1982	1982
4	0.1	1983	1983
7	0.2	1984	1984
11	0.3	1985	1985
20	0.5	1986	1986
20	0.5	1987	1987
17	0.5	1988	1988
16	0.4	1989	1989
26	0.7	1990	1990
27	0.7	1991	1991
30	0.8	1992	1992
58	1.6	1993	1993
62	1.7	1994	1994
91	2.5	1995	1995
100	2.7	1996	1996
104	2.8	1997	1997
137	3.7	1998	1998
218	5.9	1999	1999
277	7.5	2000	2000
187	5.1	2001	2001
138	3.7	2002	2002
167	4.5	2003	2003
195	5.3	2004	2004
345	9.3	2005	2005
347	9.4	2006	2006
447	12.1	2007	2007
202	5.5	2008	2008
189	5.1	2009	2009
134	3.6	2010	2010
41	1.1	2011	2011
50	1.4	9999	Unknown

Variable	Name	Format	Type	Length
v1003	CAB STYLE	V1003_F	Numeric	4

N	Prcnt	Code	Label
3,493	94.4	1	Conventional
156	4.2	2	Cabover or cab-forward
50	1.4	9	Unknown

Variable	Name	Format	Type	Length
v1006	TRUCK MODEL	$CHAR10	Char	10

N	Prcnt	Code	Label
74	2.0		Unrecorded
1	0.0	1552SC	
		-	Model name or number
1	0.0	WXLL	

Variable	Name	Format	Type	Length
v1007	POWER UNIT TYPE	V1007_F	Numeric	4

N	Prcnt	Code	Label
1,298	35.1	1	Straight truck
2,347	63.4	8	Tractor
54	1.5	9	Unknown

Variable	Name	Format	Type	Length
v1008	STRT TRUCK BODY STYLE	V1008_F	Numeric	4

STRAIGHT TRUCK BODY STYLE

N	Prcnt	Code	Label
2,347	63.4	0	Not applicable (tractor)
223	6.0	1	Van
1	0.0	2	Open top van
32	0.9	3	Refrigerated van
1	0.0	4	Livestock carrier
104	2.8	5	Flatbed
26	0.7	7	Flatbed with equipment

N	Prcnt	Code	Label
15	0.4	8	Flatbed with sides
5	0.1	9	Pole/logging
63	1.7	10	Tank: liquid/gas
1	0.0	11	Tank: dry bulk
9	0.2	12	Auto carrier
245	6.6	13	Dump
3	0.1	14	Bottom dump/hopper bottom
97	2.6	15	Garbage/refuse
19	0.5	17	Concrete mixer
232	6.3	18	Pickup
211	5.7	95	Other
65	1.8	99	Unknown

Variable	Name	Format	Type	Length
v1009	STRT TRUCK OTHER BODY	$CHAR10	Char	10

STRAIGHT TRUCK OTHER BODY

N	Prcnt	Code	Label
3,395	91.8		Unrecorded
2	0.1	ARMOREDCAR	
		-	Specific body style
1	0.0	YARD	

Variable	Name	Format	Type	Length
v1010	POWER UNIT NO. OF AXLES	V1010_F	Numeric	4

POWER UNIT NUMBER OF AXLES

N	Prcnt	Code	Label
1,008	27.3	2	2 axles
2,525	68.3	3	3 axles
85	2.3	4	4 axles
19	0.5	5	5 axles
2	0.1	6	6 axles
60	1.6	9	Unknown

Variable	Name	Format	Type	Length
v1015	POWER UNIT CARGO	V1015_F	Numeric	4

N	Prcnt	Code	Label
501	13.5	0	Empty
120	3.2	1	General freight
24	0.6	2	Household goods
11	0.3	3	Building materials
5	0.1	4	Metal: coils, sheets, etc
5	0.1	5	Heavy machinery
16	0.4	6	Large objects
32	0.9	7	Motor vehicles
10	0.3	8	Piggyback/towaway
9	0.2	9	Gases in bulk
206	5.6	10	Solids in bulk
41	1.1	11	Liquids in bulk
0	0.0	12	Explosives
10	0.3	13	Logs/poles/lumber
26	0.7	14	Refrigerated food
15	0.4	16	Farm products
2	0.1	17	Live animals
182	4.9	18	Other
10	0.3	94	Not intended for cargo
5	0.1	95	Cargo, unknown type
2,342	63.3	98	Not applicable (not a straight truck)
127	3.4	99	Unknown

Variable	Name	Format	Type	Length
v1017	1ST TRAILER TYPE	V1017_F	Numeric	4

FIRST TRAILER TYPE

N	Prcnt	Code	Label
2,272	61.4	1	Semitrailer
28	0.8	2	Full trailer
138	3.7	3	Other
1,196	32.3	4	None
65	1.8	9	Unknown

Variable	Name	Format	Type	Length
v1018	1ST TRAILER NO. OF AXLES	V1018_F	Numeric	4

FIRST TRAILER NUMBER OF AXLES

N	Prcnt	Code	Label
106	2.9	1	1 axle
2,154	58.2	2	2 axles
98	2.6	3	3 axles
25	0.7	4	4 axles
2	0.1	5	5 axles
1	0.0	6	6 axles
1	0.0	8	8 axles
65	1.8	97	Unknown if had first trailer
1,196	32.3	98	Not applicable (no first trailer)
51	1.4	99	Unknown

Variable	Name	Format	Type	Length
v1023	1ST TRAILER BODY	V1023_F	Numeric	4

FIRST TRAILER BODY

N	Prcnt	Code	Label
1,261	34.1	0	None or unknown if had first trailer
946	25.6	1	Van
37	1.0	2	Open top van
291	7.9	3	Refrigerated van
42	1.1	4	Livestock carrier
332	9.0	5	Flatbed
49	1.3	6	Lowboy
1	0.0	7	Flatbed with equipment
16	0.4	8	Flatbed with sides
98	2.6	9	Pole/logging
194	5.2	10	Tank: liquid/gas
35	0.9	11	Tank: dry bulk
20	0.5	12	Auto carrier
186	5.0	13	Dump
112	3.0	14	Bottom dump/hopper bottom
9	0.2	15	Garbage/refuse

N	Prcnt	Code	Label
48	1.3	95	Other
22	0.6	99	Unknown

Variable	Name	Format	Type	Length
v1024	1ST TRAILER OTHER BODY	$CHAR10	Char	10

FIRST TRAILER OTHER BODY STYLE

N	Prcnt	Code	Label
3,528	95.4		Unknown or not applicable
1	0.0	AUTO DOLLY	
		-	Specific body style
3	0.1	VACUUM	

Variable	Name	Format	Type	Length
v1025	1ST TRAILER CARGO	V1025_F	Numeric	4

FIRST TRAILER CARGO

N	Prcnt	Code	Label
623	16.8	0	Empty
601	16.2	1	General freight
14	0.4	2	Household goods
37	1.0	3	Building materials
67	1.8	4	Metal: coils, sheets, etc
71	1.9	5	Heavy machinery
44	1.2	6	Large objects
29	0.8	7	Motor vehicles
3	0.1	8	Piggyback/towaway
16	0.4	9	Gases in bulk
248	6.7	10	Solids in bulk
120	3.2	11	Liquids in bulk
106	2.9	13	Logs/poles/lumber
200	5.4	14	Refrigerated food
1	0.0	15	Mobile home
47	1.3	16	Farm products
28	0.8	17	Live animals
17	0.5	18	Other

N	Prcnt	Code	Label
9	0.2	94	Not for cargo
31	0.8	95	Cargo, unknown type
65	1.8	96	Unknown if had first trailer
1,196	32.3	98	Not applicable (no first trailer)
126	3.4	99	Unknown

Variable	Name	Format	Type	Length
v1027	2ND TRAILER TYPE	V1027_F	Numeric	4

SECOND TRAILER TYPE

N	Prcnt	Code	Label
69	1.9	1	Semitrailer
24	0.6	2	Full trailer
3	0.1	3	Other
3,537	95.6	4	None
66	1.8	9	Unknown

Variable	Name	Format	Type	Length
v1028	2ND TRAILER NO. OF AXLES	V1028_F	Numeric	4

SECOND TRAILER NUMBER OF AXLES

N	Prcnt	Code	Label
75	2.0	2	2 axles
10	0.3	3	3 axles
4	0.1	4	4 axles
2	0.1	6	6 axles
66	1.8	97	Unknown if had second trailer
3,537	95.6	98	Not applicable (no second trailer)
5	0.1	99	Unknown

Variable	Name	Format	Type	Length
v1033	2ND TRAILER BODY	V1033_F	Numeric	4

SECOND TRAILER BODY STYLE

N	Prcnt	Code	Label
3,603	97.4	0	None or unknown if had second trailer
51	1.4	1	Van
1	0.0	2	Open top van
2	0.1	3	Refrigerated van
10	0.3	5	Flatbed
1	0.0	6	Lowboy
1	0.0	8	Flatbed with sides
2	0.1	10	Tank: liquid/gas
4	0.1	11	Tank: dry bulk
4	0.1	13	Dump
12	0.3	14	Bottom dump/hopper bottom
6	0.2	95	Other
2	0.1	99	Unknown

Variable	Name	Format	Type	Length
v1034	2ND TRAILER OTHER BODY	$CHAR10	Char	10

SECOND TRAILER OTHER BODY STYLE

N	Prcnt	Code	Label
3,691	99.8		Unknown or not applicable
1	0.0	BOOSTER	
		-	Specific body style
2	0.1	TOMATO TUB	

Variable	Name	Format	Type	Length
v1035	2ND TRAILER CARGO	V1035_F	Numeric	4

SECOND TRAILER CARGO

N	Prcnt	Code	Label
15	0.4	0	Empty
43	1.2	1	General freight
1	0.0	2	Household goods

N	Prcnt	Code	Label
2	0.1	6	Large objects
13	0.4	10	Solids in bulk
2	0.1	13	Logs/poles/lumber
2	0.1	14	Refrigerated food
6	0.2	16	Farm products
1	0.0	94	Not for cargo
66	1.8	96	Unknown if had second trailer
3,537	95.6	98	Not applicable (no second trailer)
11	0.3	99	Unknown

Variable	Name	Format	Type	Length
v1037	3RD TRAILER TYPE	V1037_F	Numeric	4

THIRD TRAILER TYPE

N	Prcnt	Code	Label
3	0.1	1	Semitrailer
2	0.1	3	Other
3,629	98.1	4	None
65	1.8	9	Unknown

Variable	Name	Format	Type	Length
v1038	3RD TRAILER NO. OF AXLES	V1038_F	Numeric	4

THIRD TRAILER NUMBER OF AXLES

N	Prcnt	Code	Label
5	0.1	2	2 axles
65	1.8	97	Unknown if had third trailer
3,629	98.1	98	Not applicable (no third trailer)

Variable	Name	Format	Type	Length
v1043	3RD TRAILER BODY	V1043_F	Numeric	4

THIRD TRAILER BODY STYLE

N	Prcnt	Code	Label
3,694	99.9	0	None or unknown if had third trailer

N	Prcnt	Code	Label
2	0.1	1	Van
1	0.0	14	Bottom dump/hopper bottom
2	0.1	95	Other

Variable	Name	Format	Type	Length
v1044	3RD TRAILER OTHER BODY	$CHAR10	Char	10

THIRD TRAILER OTHER BODY STYLE

N	Prcnt	Code	Label
3,697	99.9		Unknown or not applicable
1	0.0	JEEP	JEEP
1	0.0	STINGER	STINGER

Variable	Name	Format	Type	Length
v1045	3RD TRAILER CARGO	V1045_F	Numeric	4

THIRD TRAILER CARGO

N	Prcnt	Code	Label
2	0.1	1	General freight
1	0.0	10	Solids in bulk
1	0.0	94	Not for cargo
65	1.8	96	Unknown if had third trailer
3,629	98.1	98	Not applicable (no third trailer)
1	0.0	99	Unknown

Variable	Name	Format	Type	Length
v1047	VEHICLE CONFIGURATION	$CHAR10	Char	10

VEHICLE CONFIGURATION BY UNIT

N	Prcnt	Code	Label
54	1.5		Unknown or unspecified
1,136	30.7	R	
		-	Unit descriptor code
2	0.1	TW	

Variable	Name	Format	Type	Length
v1048	VEHICLE COMBINATION CODE	V1048_F	Numeric	4

N	Prcnt	Code	Label
			Straight truck, no or 1 cargo-carrying trailer
1,136	30.7	1	Straight truck only
27	0.7	2	Straight truck and full trailer
85	2.3	3	Straight truck and other (non-full trailer)
47	1.3	4	Straight truck and other trailer with gooseneck hitch
0	0.0	5	Straight truck and A-dolly and semitrailer
3	0.1	6	Straight truck wrecker with towed unit
0	0.0	9	Straight truck, unknown if pulling trailer
			Straight truck, 2 cargo-carrying trailers
0	0.0	10	Straight truck and full trailer and other trailer
0	0.0	11	Straight truck and other trailer and other trailer
0	0.0	12	Straight truck and 2 full trailers
0	0.0	13	Straight truck, other trailer with gooseneck hitch, A-dolly, other trailer
0	0.0	14	Straight truck, other trailer with gooseneck hitch, other trailer
			Tractor, no cargo-carrying trailers
64	1.7	20	Bobtail tractor
2	0.1	21	Tractor carrying cargo
0	0.0	22	Tractor and A-dolly
			Tractor, 1 cargo-carrying trailer
2,178	58.9	30	Tractor and semitrailer
2	0.1	31	Tractor and other (non-semitrailer)
0	0.0	32	Tractor and semitrailer and A-dolly
0	0.0	33	Tractor and full trailer
1	0.0	34	Tractor and gooseneck and other
			Tractor, 2 cargo-carrying trailers
53	1.4	40	Tractor and semitrailer and A-dolly and semitrailer
2	0.1	41	Tractor and semitrailer and B-dolly and semitrailer
1	0.0	42	Tractor and semitrailer and C-dolly and semitrailer
7	0.2	43	Tractor and semitrailer with unknown dolly and semitrailer
24	0.6	44	Tractor and semitrailer and full trailer

N	Prcnt	Code	Label
0	0.0	45	Tractor and full trailer and full trailer
2	0.1	46	Tractor and semitrailer and other trailer
0	0.0	47	Tractor and ballhitch and 2 other trailers
0	0.0	48	Tractor and semitrailer and A-dolly and unknown trailer
2	0.1	49	Tractor and semitrailer and unknown trailer
			Tractor, 3 cargo-carrying trailers
3	0.1	50	Tractor and 3 trailers (A-dollies)
0	0.0	51	Tractor, semitrailer, A-dolly, semitrailer, full trailer
0	0.0	52	Tractor, semitrailer, two full trailers
0	0.0	53	Tractor and semitrailer and C-dolly and semitrailer and A-dolly and semitrailer
1	0.0	54	Tractor and semitrailer and unknown dolly and semitrailer and unknown dolly and semitrailer
			Combinations for oversize/overweight loads
0	0.0	60	Tractor and jeep and semitrailer and jeep
0	0.0	61	Tractor and semitrailer and jeep
1	0.0	62	Tractor and jeep and semitrailer
0	0.0	63	Tractor and jeep and full trailer and jeep
1	0.0	64	Tractor and jeep and semitrailer and other
0	0.0	65	Tractor and jeep with cargo
0	0.0	66	Tractor and semitrailer and full trailer and straight truck
0	0.0	67	Tractor and jeep and other
1	0.0	68	Tractor and jeep and other and jeep
			Unusual tractor combinations
0	0.0	70	Tractor towing tractor *
0	0.0	71	Tractor towing straight truck *
0	0.0	72	Tractor towing tractor-semitrailer*
2	0.1	73	Tractor with one saddlemount tractor
0	0.0	74	Tractor with two saddlemount tractors
0	0.0	75	Tractor with three saddlemount tractors
0	0.0	78	Tractor with saddlemount tractor and P van
			Unusual straight truck combinations
0	0.0	80	Straight truck towing straight truck *
0	0.0	81	Straight truck pulling tractor-semitrailer
0	0.0	82	Wrecker and doubles

N	Prcnt	Code	Label
0	0.0	83	Wrecker towing straight and full trailer
0	0.0	84	Straight with two saddlemount straight trucks
0	0.0	85	Wrecker towing other trailer
0	0.0	86	Wrecker and tow and tow and tow
0	0.0	87	Wrecker and A-dolly
54	1.5	99	Unknown

*Towed with all wheels on the ground.

Variable	Name	Format	Type	Length
v1049	NO. OF TRAILERS	V1049_F	Numeric	4

NUMBER OF TRAILERS

N	Prcnt	Code	Label
1,196	32.3	0	No trailer
2,339	63.2	1	1 trailer
91	2.5	2	2 trailers
5	0.1	3	3 trailers
68	1.8	9	Unknown

Variable	Name	Format	Type	Length
v1050	GROSS VEHICLE WEIGHT RATING	V1050_F	Numeric	4

N	Prcnt	Code	Label
364	9.8	3	10,001 - 14,000 lbs.
96	2.6	4	14,001 - 16,000 lbs.
85	2.3	5	16,001 - 19,500 lbs.
168	4.5	6	19,501 - 26,000 lbs.
227	6.1	7	26,001 - 33,000 lbs.
2,696	72.9	8	33,001 lbs. or more
63	1.7	9	Unknown

Variable	Name	Format	Type	Length
v1050C	GROSS COMBINATION WEIGHT RATING	V1050C_F	Numeric	4

N	Prcnt	Code	Label
715	19.3	2	10,000-26,000 lbs
2,924	79.0	3	Greater than 26,000 lbs
60	1.6	9	Unknown

Variable	Name	Format	Type	Length
v1055	SPECIFIC CARGO	$CHAR20	Char	20

ACTUAL CARGO CARRIED

N	Prcnt	Code	Label
1,673	45.2		
1	0.0	ADHESIVE IN DRUMS	
		-	Actual cargo
1	0.0	WOOD	

Variable	Name	Format	Type	Length
v1056	CARGO SPILLAGE	V1056_F	Numeric	4

N	Prcnt	Code	Label
2,993	80.9	0	No spillage
469	12.7	1	Spillage of nonhazardous cargo
30	0.8	2	Spillage of hazardous cargo
207	5.6	9	Unknown

Variable	Name	Format	Type	Length
v1057	AREA OF OPERATION	V1057_F	Numeric	4

N	Prcnt	Code	Label
2,431	65.7	1	Interstate
768	20.8	2	Intrastate
146	3.9	5	Personal use only
70	1.9	6	Government owned
22	0.6	7	Daily rental
262	7.1	9	Unknown

Variable	Name	Format	Type	Length
v1058	OPERATING AUTHORITY	V1058_F	Numeric	4

N	Prcnt	Code	Label
1,231	33.3	1	Private
2,071	56.0	2	For hire
146	3.9	5	Personal use only
70	1.9	6	Government owned
22	0.6	7	Daily rental
159	4.3	9	Unknown

Variable	Name	Format	Type	Length
v1059	ACCIDENT TYPE	V1059_F	Numeric	4

N	Prcnt	Code	Label
0	0.0	0	No impact
		-	Diagram number (see appendix)
36	1.0	97	Untripped rollover
438	11.8	98	Other accident type
83	2.2	99	Unknown

Variable	Name	Format	Type	Length
v1061	TRIP TYPE	V1061_F	Numeric	4

N	Prcnt	Code	Label
1,243	33.6	1	Local delivery
418	11.3	2	51 to 100 miles
188	5.1	3	101 to 150 miles
163	4.4	4	151 to 200 miles
442	11.9	5	201 to 500 miles
438	11.8	6	Over 500 miles
97	2.6	7	Unknown over-the-road trip distance
710	19.2	9	Unknown

Variable	Name	Format	Type	Length
v1062	HOURS DRIVING	V1062_F	Numeric	4

N	Prcnt	Code	Label
516	13.9	1	1 hour
332	9.0	2	2 hours
209	5.7	3	3 hours
200	5.4	4	4 hours
139	3.8	5	5 hours
162	4.4	6	6 hours
91	2.5	7	7 hours
132	3.6	8	8 hours
44	1.2	9	9 hours
38	1.0	10	10 hours
9	0.2	11	11 hours
7	0.2	12	12 hours
1	0.0	13	13 hours
3	0.1	14	14 hours
1	0.0	15	15 hours
1	0.0	17	17 hours
1	0.0	32	32 hours
401	10.8	96	Unknown but legal
12	0.3	97	Unknown but not legal
1,400	37.8	99	Unknown

Variable	Name	Format	Type	Length
v1063	INTERVIEW CONDUCTED	V1063_F	Numeric	4

N	Prcnt	Code	Label
3,263	88.2	1	Yes
436	11.8	2	No

Variable	Name	Format	Type	Length
v1064	POLICE REPORT	V1064_F	Numeric	4

N	Prcnt	Code	Label
3,489	94.3	1	Yes
210	5.7	2	No

Variable	Name	Format	Type	Length
v1065	FAX/MAIL	V1065_F	Numeric	4

N	Prcnt	Code	Label
367	9.9	1	Yes
3,332	90.1	2	No

Variable	Name	Format	Type	Length
v1066	1ST QUESTION DERIVED	V1066_F	Numeric	4

FIRST QUESTION DERIVED

N	Prcnt	Code	Label
3,695	99.9	0	None
1	0.0	16	Question 16
3	0.1	19	Question 19

Variable	Name	Format	Type	Length
v1067	2ND QUESTION DERIVED	V1067_F	Numeric	4

SECOND QUESTION DERIVED

N	Prcnt	Code	Label
3,699	100.0	0	None

Variable	Name	Format	Type	Length
v1068	3RD QUESTION DERIVED	V1068_F	Numeric	4

THIRD QUESTION DERIVED

N	Prcnt	Code	Label
3,699	100.0	0	None

Variable	Name	Format	Type	Length
v1069	4TH QUESTION DERIVED	V1069_F	Numeric	4

FOURTH QUESTION DERIVED

N	Prcnt	Code	Label
3,699	100.0	0	None

Variable	Name	Format	Type	Length
v1070	5TH QUESTION DERIVED	V1070_F	Numeric	4

FIFTH QUESTION DERIVED

N	Prcnt	Code	Label
3,699	100.0	0	None

Variable	Name	Format	Type	Length
v1091	HAZARDOUS MATERIALS PLACARD	V1091_F	Numeric	4

N	Prcnt	Code	Label
104	2.8	1	Yes
3,437	92.9	2	No
158	4.3	9	Unknown

Variable	Name	Format	Type	Length
v1092	HAZARDOUS MATERIALS CLASS	V1092_F	Numeric	4

N	Prcnt	Code	Label
0	0.0	1	Explosives
29	0.8	2	Compressed gases
57	1.5	3	Flammable liquids
2	0.1	4	Flammable solids
1	0.0	5	Oxidizing substances
0	0.0	6	Poisonous substances
0	0.0	7	Radioactive materials
10	0.3	8	Corrosive liquids
2	0.1	9	Miscellaneous hazardous substances
3,437	92.9	98	Not applicable
161	4.4	99	Unknown

Variable	Name	Format	Type	Length
v1093	HAZARDOUS PLACARD 4-DIGIT NUMBER	V1093_F	Numeric	4

N	Prcnt	Code	Label
3	0.1	1005	
		-	Hazardous placard number
1	0.0	3257	
3,437	92.9	9998	Not applicable
164	4.4	9999	Unknown

Variable	Name	Format	Type	Length
v1101	HEADWAY DETECTION/FORWARD CRASH WARNING	V1101_8F	Numeric	4

N	Prcnt	Code	Label
26	0.7	1	Yes
2,547	68.9	2	No
1,126	30.4	9	Unknown

Variable	Name	Format	Type	Length
v1102	SIDE/OBJECT DETECTION	V1101_8F	Numeric	4

N	Prcnt	Code	Label
39	1.1	1	Yes
2,547	68.9	2	No
1,113	30.1	9	Unknown

Variable	Name	Format	Type	Length
v1103	LANE DEPARTURE WARNING	V1101_8F	Numeric	4

N	Prcnt	Code	Label
39	1.1	1	Yes
2,536	68.6	2	No
1,124	30.4	9	Unknown

Variable	Name	Format	Type	Length
v1104	ROLLOVER WARNING	V1101_8F	Numeric	4

N	Prcnt	Code	Label
61	1.6	1	Yes
2,490	67.3	2	No
1,148	31.0	9	Unknown

Variable	Name	Format	Type	Length
v1105	ELECTRONIC STABILITY CONTROL	V1101_8F	Numeric	4

N	Prcnt	Code	Label
129	3.5	1	Yes
2,288	61.9	2	No
1,282	34.7	9	Unknown

Variable	Name	Format	Type	Length
v1106	POWER UNIT TRACKING	V1101_8F	Numeric	4

N	Prcnt	Code	Label
548	14.8	1	Yes
2,011	54.4	2	No
1,140	30.8	9	Unknown

Variable	Name	Format	Type	Length
v1107	TRAILER TRACKING	V1101_8F	Numeric	4

N	Prcnt	Code	Label
129	3.5	1	Yes
2,326	62.9	2	No
1,244	33.6	9	Unknown

Variable	Name	Format	Type	Length
v1108	SPEED LIMITER	V1101_8F	Numeric	4

N	Prcnt	Code	Label
42	1.1	1	Yes

N	Prcnt	Code	Label
2,530	68.4	2	No
1,127	30.5	9	Unknown

Variable	Name	Format	Type	Length
v1109	AUTOMATIC COLLISION AVOIDANCE BRAKING	V1101_8F	Numeric	4

N	Prcnt	Code	Label
989	26.7	1	Yes
1,352	36.6	2	No
1,358	36.7	9	Unknown

Variable	Name	Format	Type	Length
v1111	DRIVER COMPENSATION	V1111_F	Numeric	4

N	Prcnt	Code	Label
223	6.0	1	Percent of gross trip revenue
789	21.3	2	By hour
518	14.0	3	By mile
93	2.5	4	By hour and mile
356	9.6	5	By load
340	9.2	6	Other
1,380	37.3	9	Unknown

Variable	Name	Format	Type	Length
v1112	DRIVER COMPENSATION OTHER	$CHAR15	Char	15

N	Prcnt	Label
1	0.0	BARTER SERVICES
2	0.1	BASE+COMMISSION
1	0.0	BY ACTIVITY
8	0.2	BY DAY
1	0.0	BY DELIVERY
22	0.6	BY JOB
1	0.0	BY MONTH
3	0.1	BY ROUTE
1	0.0	BY TON

N	Prcnt	Label
8	0.2	BY TRIP
6	0.2	BY WEIGHT
1	0.0	BY ZONE
10	0.3	COMMISSION
4	0.1	DAILY
2	0.1	FLAT RATE
1	0.0	FLAT RATE ROUTE
1	0.0	FLAT RATE TRIP
1	0.0	GOVERNMENT
3	0.1	HOUR + LOAD
1	0.0	HOUR + WEIGHT
1	0.0	HOUR+COMMISSION
1	0.0	LOAD + HOUR
4	0.1	LOAD + MILE
1	0.0	LOAD + PERCENT
9	0.2	MILE + ACTIVITY
2	0.1	MILE + DROPS
6	0.2	MILE + LOAD
2	0.1	MILE + PCT
1	0.0	MILE + STOP
6	0.2	MILE + STOPS
3	0.1	MILE + WEIGHT
1	0.0	MILE+DROPS+HOUR
1	0.0	MILE+PIECE+DROP
1	0.0	MILE+STOP+PCT
1	0.0	MILEHOUR+WEIGHT
13	0.4	NO COMPENSATION
84	2.3	OWNER/BUSINESS
53	1.4	PERSONAL TRIP
1	0.0	PIECE + MILE
1	0.0	PIECE RATE
2	0.1	PIECE+STOP+MILE
63	1.7	SALARY
1	0.0	SALARY + COMM
1	0.0	SALARY + MILE
1	0.0	SALE OF CARGO
2	0.1	STOLEN VEHICLE
1,979	53.5	NA
1,380	37.3	UNKNOWN

Variable	Name	Format	Type	Length
v1120	BUSINESS TYPE	$CHAR20	Char	20

N	Prcnt	Label
278	7.5	
14	0.4	AGRI PRODUCTION
24	0.6	AGRI SUPPLY
1	0.0	AMUSEMENTS
2	0.1	APIARY
6	0.2	AUTO DEALERSHIP
1	0.0	AUTO RACING
1	0.0	AUTO SALVAGE
13	0.4	AUTO TRANSPORT
9	0.2	BAKERY
1	0.0	BEVERAGE PRODUCTION
2	0.1	BINDERY
1	0.0	BOAT DEALERSHIP
2	0.1	CARCASS REMOVAL
5	0.1	CHEM DISTRIBUTION
3	0.1	CHEM TRANSPORT
162	4.4	CONSTRUCTION
69	1.9	CONSTRUCTION SUPPLY
1	0.0	CONTAINER SERVICE
11	0.3	DAIRY PRODUCTION
2	0.1	DEFORESTATION
188	5.1	DISTRIBUTION
5	0.1	DRILLING + BLASTING
2	0.1	EDUCATION
2	0.1	ENERGY EXPLORATION
2	0.1	ENERGY PRODUCTION
1	0.0	ENVIRONMENTAL SVCS
23	0.6	EQUIPMENT DEALER
17	0.5	EQUIPMENT RENTAL
7	0.2	FABRICATION
13	0.4	FARM COOPERATIVE
95	2.6	FARMING
46	1.2	FUEL DISTRIBUTION
23	0.6	FUEL TRANSPORT
72	1.9	GOVERNMENT

N	Prcnt	Label
1	0.0	GRAIN MILLING
6	0.2	HARVESTING
9	0.2	INDUSTRIAL GASES
3	0.1	INTERMENT
20	0.5	LANDSCAPING
16	0.4	LANDSCAPING SUPPLY
9	0.2	LIVESTOCK
41	1.1	LOGGING OPERATION
35	0.9	MAINTENANCE + REPAIR
60	1.6	MANUFACTURING
1	0.0	MEDICAL WASTE
2	0.1	MINING
1	0.0	MINING SUPPLY
4	0.1	MOBILE HOME SERVICE
8	0.2	MOVING + STORAGE
2	0.1	NURSERY
55	1.5	OIL FIELD SERVICE
1	0.0	OIL PRODUCTION
40	1.1	PACKAGE DELIVERY
1	0.0	PEST CONTROL
9	0.2	POULTRY PRODUCTION
6	0.2	RAILROAD
7	0.2	RANCHING
35	0.9	RECYCLING
29	0.8	RENTAL/LEASING
5	0.1	REPOSSESSION SERVICE
1	0.0	RESOURCE MANAGEMENT
17	0.5	RETAIL STORE
15	0.4	ROAD MAINTENANCE
3	0.1	SECURE TRANSPORT
24	0.6	SERVICE + REPAIR
1	0.0	SHIPPING YARD
16	0.4	SMALL BUSINESS
2	0.1	SOD FARM
1	0.0	STONE PROCESSING
60	1.6	TOWING
21	0.6	TRADESMAN
9	0.2	TREE TRIMMING
2	0.1	TRUCK DEALERSHIP

N	Prcnt	Label
1,887	51.0	TRUCKING
7	0.2	UNIFORM SUPPLY
21	0.6	UTILITY
9	0.2	VENDOR
91	2.5	WASTE COLLECTION
2	0.1	WOOD MILLING

Variable	Name	Format	Type	Length
v1121	SPEED LIMITER SETTING	2	Numeric	4

N	Prcnt	Code	Label
1	0.0	40	40 mph
2	0.1	45	45 mph
1	0.0	50	50 mph
19	0.5	55	55 mph
4	0.1	56	56 mph
2	0.1	57	57 mph
9	0.2	58	58 mph
37	1.0	60	60 mph
5	0.1	61	61 mph
85	2.3	62	62 mph
21	0.6	63	63 mph
18	0.5	64	64 mph
284	7.7	65	65 mph
14	0.4	66	66 mph
33	0.9	67	67 mph
120	3.2	68	68 mph
41	1.1	69	69 mph
102	2.8	70	70 mph
2	0.1	71	71 mph
31	0.8	72	72 mph
6	0.2	73	73 mph
2	0.1	74	74 mph
17	0.5	75	75 mph
4	0.1	76	76 mph
1	0.0	77	77 mph
2	0.1	78	78 mph

N	Prcnt	Code	Label
3	0.1	80	80 mph
2,833	76.6	99	Unknown

Variable	Name	Format	Type	Length
v1125	YEARS OF TRUCK DRIVING EXPERIENCE	2	Numeric	4

N	Prcnt	Code	Label
44	1.2	1	1 year
33	0.9	2	2 years
48	1.3	3	3 years
44	1.2	4	4 years
77	2.1	5	5 years
40	1.1	6	6 years
42	1.1	7	7 years
39	1.1	8	8 years
29	0.8	9	9 years
179	4.8	10	10 years
24	0.6	11	11 years
53	1.4	12	12 years
31	0.8	13	13 years
17	0.5	14	14 years
117	3.2	15	15 years
13	0.4	16	16 years
14	0.4	17	17 years
35	0.9	18	18 years
10	0.3	19	19 years
227	6.1	20	20 years
9	0.2	21	21 years
11	0.3	22	22 years
10	0.3	23	23 years
5	0.1	24	24 years
80	2.2	25	25 years
11	0.3	26	26 years
7	0.2	27	27 years
8	0.2	28	28 years
6	0.2	29	29 years
134	3.6	30	30 years
2	0.1	31	31 years

N	Prcnt	Code	Label
10	0.3	32	32 years
11	0.3	33	33 years
6	0.2	34	34 years
39	1.1	35	35 years
7	0.2	36	36 years
3	0.1	37	37 years
13	0.4	38	38 years
1	0.0	39	39 years
56	1.5	40	40 years
2	0.1	41	41 years
4	0.1	42	42 years
2	0.1	43	43 years
8	0.2	45	45 years
2	0.1	47	47 years
3	0.1	50	50 years
1	0.0	51	51 years
1	0.0	55	55 years
2	0.1	60	60 years
2,129	57.6	99	Unknown

Variable	Name	Format	Type	Length
v1126	YEARS OF DRIVING EXPERIENCE FOR COMPANY	2	Numeric	4

N	Prcnt	Code	Label
338	9.1	1	1 year
266	7.2	2	2 years
183	4.9	3	3 years
131	3.5	4	4 years
182	4.9	5	5 years
108	2.9	6	6 years
67	1.8	7	7 years
80	2.2	8	8 years
45	1.2	9	9 years
124	3.4	10	10 years
24	0.6	11	11 years
39	1.1	12	12 years
21	0.6	13	13 years

N	Prcnt	Code	Label
11	0.3	14	14 years
58	1.6	15	15 years
12	0.3	16	16 years
3	0.1	17	17 years
14	0.4	18	18 years
7	0.2	19	19 years
62	1.7	20	20 years
7	0.2	21	21 years
9	0.2	22	22 years
4	0.1	23	23 years
6	0.2	24	24 years
12	0.3	25	25 years
3	0.1	26	26 years
2	0.1	27	27 years
4	0.1	28	28 years
1	0.0	29	29 years
21	0.6	30	30 years
2	0.1	31	31 years
6	0.2	32	32 years
3	0.1	33	33 years
2	0.1	34	34 years
12	0.3	35	35 years
2	0.1	36	36 years
3	0.1	37	37 years
1	0.0	38	38 years
11	0.3	40	40 years
2	0.1	43	43 years
2	0.1	45	45 years
1	0.0	50	50 years
1	0.0	52	52 years
1	0.0	55	55 years
1,806	48.8	99	Unknown

APPENDIX

ACCIDENT TYPES (INCLUDES INTENT)

CATEGORY	CONFIGURATION			
I SINGLE DRIVER	A. RIGHT ROADSIDE DEPARTURE	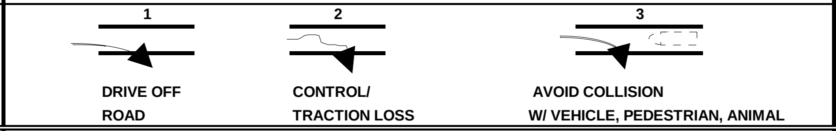 1 DRIVE OFF ROAD; 2 CONTROL/ TRACTION LOSS; 3 AVOID COLLISION W/ VEHICLE, PEDESTRIAN, ANIMAL	4 SPECIFICS OTHER	5 SPECIFICS UNKNOWN
	B. LEFT ROADSIDE DEPARTURE	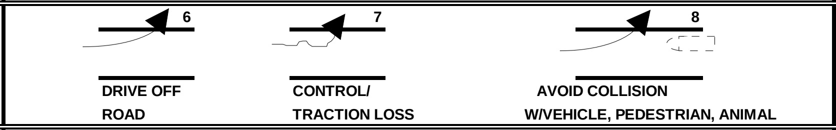 6 DRIVE OFF ROAD; 7 CONTROL/ TRACTION LOSS; 8 AVOID COLLISION W/VEHICLE, PEDESTRIAN, ANIMAL	9 SPECIFICS OTHER	10 SPECIFICS UNKNOWN
	C. FORWARD IMPACT	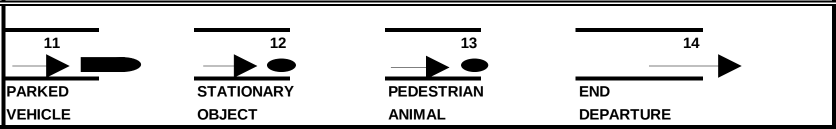 11 PARKED VEHICLE; 12 STATIONARY OBJECT; 13 PEDESTRIAN ANIMAL; 14 END DEPARTURE	15 SPECIFICS OTHER	16 SPECIFICS UNKNOWN
II SAME TRAFFICWAY SAME DIRECTION	D. REAR-END	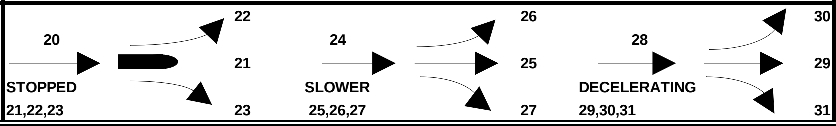 20, 21, 22, 23 STOPPED 21,22,23; 24, 25, 26, 27 SLOWER 25,26,27; 28, 29, 30, 31 DECELERATING 29,30,31	30 (EACH - 32) SPECIFICS OTHER	(EACH - 33) SPECIFICS UNKNOWN
	E. FORWARD IMPACT	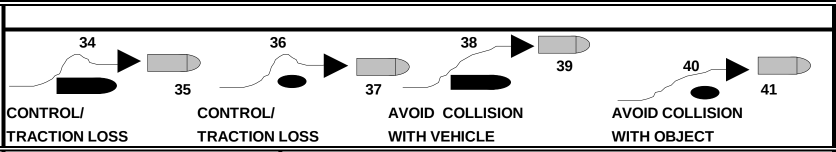 34, 35 CONTROL/ TRACTION LOSS; 36, 37 CONTROL/ TRACTION LOSS; 38, 39 AVOID COLLISION WITH VEHICLE; 40, 41 AVOID COLLISION WITH OBJECT	(EACH - 42) SPECIFICS OTHER	(EACH - 43) SPECIFICS UNKNOWN
	F. SIDESWIPE ANGLE	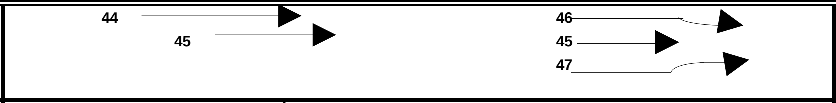 44, 45; 46, 45, 47	(EACH - 48) SPECIFICS OTHER	(EACH - 49) SPECIFICS UNKNOWN
III SAME TRAFFICWAY OPPOSITE DIRECTION	G. HEAD-ON	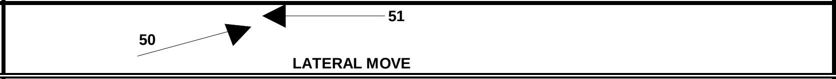 50, 51 LATERAL MOVE	(EACH - 52) SPECIFICS OTHER	(EACH - 53) SPECIFICS UNKNOWN
	H. FORWARD IMPACT	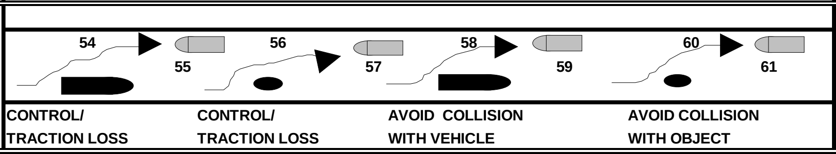 54, 55 CONTROL/ TRACTION LOSS; 56, 57 CONTROL/ TRACTION LOSS; 58, 59 AVOID COLLISION WITH VEHICLE; 60, 61 AVOID COLLISION WITH OBJECT	(EACH - 62) SPECIFICS OTHER	(EACH - 63) SPECIFICS UNKNOWN
	I. SIDESWIPE/ ANGLE	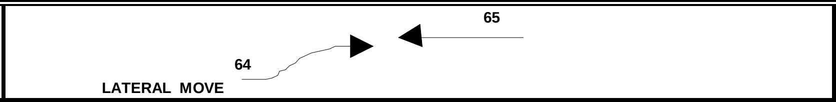 64, 65 LATERAL MOVE	(EACH - 66) SPECIFICS OTHER	(EACH - 67) SPECIFICS UNKNOWN
IV CHANGE TRAFFICWAY VEHICLE TURNING	J. TURN ACROSS PATH	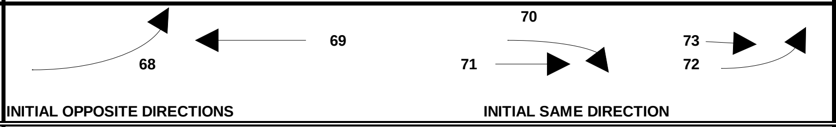 68, 69 INITIAL OPPOSITE DIRECTIONS; 70, 71, 72, 73 INITIAL SAME DIRECTION	(EACH - 74) SPECIFICS OTHER	(EACH - 75) SPECIFICS UNKNOWN
	K. TURN INTO PATH	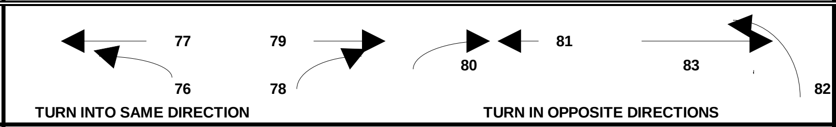 76, 77, 78, 79 TURN INTO SAME DIRECTION; 80, 81, 82, 83 TURN IN OPPOSITE DIRECTIONS	82 (EACH - 84) SPECIFICS OTHER	(EACH - 85) SPECIFICS UNKNOWN
V INTERSECTING PATH-VEHICLE DAMAGE	L. STRAIGHT PATHS	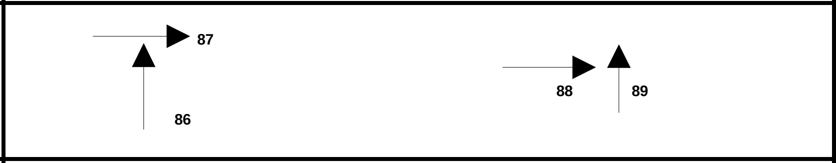 86, 87; 88, 89	(EACH - 90) SPECIFICS OTHER	(EACH - 91) SPECIFICS UNKNOWN
VI MISC.	M. BACKING ETC.	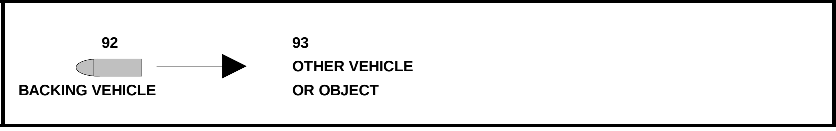 92 BACKING VEHICLE; 93 OTHER VEHICLE OR OBJECT	97 UNTRIPPED ROLLOVER<br>98 OTHER ACC TYPE<br>99 UNKNOWN ACC TYPE<br>00 NO IMPACT	

CENTER FOR NATIONAL TRUCK & BUS STATISTICS
TRUCKS INVOLVED IN FATAL ACCIDENTS SURVEY

Accident Date ______ / ______ / 2010
Month Day Year

ACCIDENT IDENTIFICATION *(fill out prior to interview)*

1. FARS State Code __ __ (1 2) State of Accident ____________

2. FARS Case No. __ __ __ __ (3 4 5 6)

3. FARS Vehicle No. __ __ (7 8)

POWER UNIT

4. Truck Make

Autocar	[ ]	01
Chevrolet	[ ]	02
Dodge	[ ]	03
Ford	[ ]	04
Freightliner	[ ]	05
GMC	[ ]	06
International/Navistar	[ ]	07
Isuzu	[ ]	08
Kenworth	[ ]	09
Mack	[ ]	10
Mercedes	[ ]	11
Mitsubishi Fuso	[ ]	12
Nissan/UD	[ ]	13
Peterbilt	[ ]	14
Volvo	[ ]	15
Western Star	[ ]	16
White	[ ]	17
WhiteGMC	[ ]	18
Sterling	[ ]	19
Other ________ *(Specify)*	[ ]	97
	9 -10	

5. Truck Model Year: __ __ __ __ (11 12 13 14)

6. Cab Style

Conventional	[ ]	1
Cab-Over-Engine/Cab Forward	[ ]	2
	15	

7. Truck Model ____________
(Name or No.) *(Specify)*

Truck Model __ __ __ __ __ __ __ __ __ __ (editor)
16 17 18 19 20 21 22 23 24 25

NOTE: Put all information and calculations on this form.

VEHICLE CONFIGURATION

	POWER UNIT		FIRST TRAILER		SECOND TRAILER		THIRD TRAILER	
8. TYPE:	Tractor	[ ] 8	Semi	[ ] 1	Semi	[ ] 1	Semi	[ ] 1
	St. Trk.	[ ] 1	Full	[ ] 2	Full	[ ] 2	Full	[ ] 2
		26	Other	[ ] 3	Other	[ ] 3	Other	[ ] 3
			None	[ ] 4	None	[ ] 4	None	[ ] 4
				27		28		29
9. NO. OF AXLES IN USE/DOWN:		__ 30		__ 31		__ 32		__ 33

10. COMBINATION CODE: __ __ __ __ __ __ __ __ __ __ (34 35 36 37 38 39 40 41 42 43)

(Key: R=straight truck; T=tractor; S=semitrailer; F=full trailer; O=other trailer; U=unknown trailer; A=A dolly; B=B train; C=C dolly; X=unknown dolly; J=jeep; G=gooseneck)

11. BODY STYLE:

POWER UNIT		FIRST TRAILER		SECOND TRAILER		THIRD TRAILER	
Tractor	[ ] 00						
Van	[ ] 01	Van	[ ] 01	Van	[ ] 01	Van	[ ] 01
Open Top Van	[ ] 02	Open Top Van	[ ] 02	Open Top Van	[ ] 02	Open Top Van	[ ] 02
Refrigerated Van	[ ] 03	Refrigerated Van	[ ] 03	Refrigerated Van	[ ] 03	Refrigerated Van	[ ] 03
Livestock Carrier	[ ] 04	Livestock Carrier	[ ] 04	Livestock Carrier	[ ] 04	Livestock Carrier	[ ] 04
Flatbed	[ ] 05	Flatbed	[ ] 05	Flatbed	[ ] 05	Flatbed	[ ] 05
Lowboy	[ ] 06	Lowboy	[ ] 06	Lowboy	[ ] 06	Lowboy	[ ] 06
Flatbed w/ equipment	[ ] 07	Flatbed w/ equipment	[ ] 07	Flatbed w/ equipment	[ ] 07	Flatbed w/ equipment	[ ] 07
Flatbed w/ sides	[ ] 08	Flatbed w/ sides	[ ] 08	Flatbed w/ sides	[ ] 08	Flatbed w/sides	[ ] 08
Pole/Logging	[ ] 09	Pole/Logging	[ ] 09	Pole/Logging	[ ] 09	Pole/Logging	[ ] 09
Tank - liquid/gas	[ ] 10	Tank - liquid/gas	[ ] 10	Tank - liquid/gas	[ ] 10	Tank - liquid/gas	[ ] 10
Tank - dry bulk	[ ] 11	Tank - dry bulk	[ ] 11	Tank - dry bulk	[ ] 11	Tank - dry bulk	[ ] 11
Auto Carrier	[ ] 12	Auto Carrier	[ ] 12	Auto Carrier	[ ] 12	Auto Carrier	[ ] 12
Dump	[ ] 13	Dump	[ ] 13	Dump	[ ] 13	Dump	[ ] 13
Bottom Dump/Hopper	[ ] 14	Bottom Dump/Hopper	[ ] 14	Bottom Dump/Hopper	[ ] 14	Bottom Dump/Hopper	[ ] 14
Garbage/Refuse	[ ] 15	Garbage/Refuse	[ ] 15	Garbage/Refuse	[ ] 15	Garbage/Refuse	[ ] 15
Concrete Mixer	[ ] 17	Concrete Mixer	[ ] 17	Concrete Mixer	[ ] 17	Concrete Mixer	[ ] 17
Pickup	[ ] 18	Other	[ ] 95	Other	[ ] 95	Other	[ ] 95
Other	[ ] 95						
	44 - 45		46 - 47		48 - 49		50 - 51

Describe "other" body style above.

Editor section:	__ __ __ __ __ __ __ __ __ __ (52 53 54 55 56 57 58 59 60 61)	__ __ __ __ __ __ __ __ __ __ (62 63 64 65 66 67 68 69 70 71)	__ __ __ __ __ __ __ __ __ __ (72 73 74 75 76 77 78 79 80 81)	__ __ __ __ __ __ __ __ __ __ (82 83 84 85 86 87 88 89 90 91)

CARGO INFORMATION

12. CARGO:

Code	POWER UNIT	FIRST TRAILER	SECOND TRAILER	THIRD TRAILER
00	Empty [ ]	Empty [ ]	Empty [ ]	Empty [ ]
01	General freight [ ]	General freight [ ]	General freight [ ]	General freight [ ]
02	Household goods [ ]	Household goods [ ]	Household goods [ ]	Household goods [ ]
03	Building materials [ ]	Building materials [ ]	Building materials [ ]	Building materials [ ]
04	Metal (coils, sheets) [ ]	Metal (coils, sheets) [ ]	Metal (coils, sheets) [ ]	Metal (coils, sheets) [ ]
05	Heavy machinery [ ]	Heavy machinery [ ]	Heavy machinery [ ]	Heavy machinery [ ]
06	Large objects [ ]	Large objects [ ]	Large objects [ ]	Large objects [ ]
07	Motor vehicles [ ]	Motor vehicles [ ]	Motor vehicles [ ]	Motor vehicles [ ]
08	Piggyback/towaway [ ]	Piggyback/towaway [ ]	Piggyback/towaway [ ]	Piggyback/towaway [ ]
09	Gases in bulk [ ]	Gases in bulk [ ]	Gases in bulk [ ]	Gases in bulk [ ]
10	Solids in bulk [ ]	Solids in bulk [ ]	Solids in bulk [ ]	Solids in bulk [ ]
11	Liquids in bulk [ ]	Liquids in bulk [ ]	Liquids in bulk [ ]	Liquids in bulk [ ]
12	Explosives [ ]	Explosives [ ]	Explosives [ ]	Explosives [ ]
13	Logs, poles, lumber [ ]	Logs, poles, lumber [ ]	Logs, poles, lumber [ ]	Logs, poles, lumber [ ]
14	Refrigerated foods [ ]	Refrigerated foods [ ]	Refrigerated foods [ ]	Refrigerated foods [ ]
15	Mobile home [ ]	Mobile home [ ]	Mobile home [ ]	Mobile home [ ]
16	Farm products [ ]	Farm products [ ]	Farm products [ ]	Farm products [ ]
17	Live animals [ ]	Live animals [ ]	Live animals [ ]	Live animals [ ]
18	Other [ ]	Other [ ]	Other [ ]	Other [ ]
94	Not for cargo [ ]	Not for cargo [ ]	Not for cargo [ ]	Not for cargo [ ]
95	Unknown type [ ]	Unknown type [ ]	Unknown type [ ]	Unknown type [ ]
	92-93	94-95	96-97	98-99

Cargo ______________________
(Specify)

Cargo _ (editor)
100 101 102 103 104 105 106 107 108 109 110 111 112 113 114 115 116 117 118 119

HAZARDOUS CARGO:

13. Any cargo that required a Hazardous Materials Placard?

Yes [ ] 1
No [ ] 2
120

14. Enter the 4-digit number from Placard: _ _ _ _
121 122 123 124

15 Enter the Hazardous Materials Class Number from bottom of Placard: _
125

16. Did any spillage of cargo result from the accident?

No spillage [ ] 0
Spillage of non-hazardous cargo [ ] 1
Spillage of hazardous cargo [ ] 2
126

17. Owner's Name ____________________

18. Owner's Business Type ____________________

Business Type (editor)

___ ___ ___ ___ ___ ___ ___ ___ ___ ___ ___ ___ ___ ___ ___ ___ ___ ___ ___ ___
127 128 129 130 131 132 133 134 135 136 137 138 139 140 141 142 143 144 145 146

19. Operating Authority at the Time of the Accident

Within twelve months before the accident, did any of your trucks carry goods interstate (across state lines)?

Yes	[ ] 1
No	[ ] 2
Personal use <u>only</u>	[ ] 5
Gov't. owned	[ ] 6
Daily rental	[ ] 7
	147

At the time of the accident, were you operating:

Private (carry own goods)	[ ] 1
For hire (carry other people's goods)	[ ] 2
Personal use <u>only</u>	[ ] 5
Govt. owned	[ ] 6
Daily rental	[ ] 7
	148

20. Intended <u>One-Way</u> Trip Distance

Local <51 miles	[ ] 1
Over-the-Road	
51 to 100 miles	[ ] 2
101 to 150 miles	[ ] 3
151 to 200 miles	[ ] 4
201 to 500 miles	[ ] 5
Greater than 500 miles	[ ] 6
Unknown over-the-road trip distance	[ ] 7
	149

21. ITS/Collision Avoidance Systems or Devices

	Yes	No	
Headway detection/forward crash warning	[ ] 1	[ ] 2	150
Automatic collision avoidance braking	[ ] 1	[ ] 2	151
Side/object detection	[ ] 1	[ ] 2	152
Lane departure warning	[ ] 1	[ ] 2	153
Rollover warning	[ ] 1	[ ] 2	154
Electronic stability control	[ ] 1	[ ] 2	155
Power unit tracking	[ ] 1	[ ] 2	156
Trailer tracking	[ ] 1	[ ] 2	157
Speed limiter	[ ] 1	[ ] 2	158

If Speed limiter is "Yes," at what speed was it set? ___ ___ mph
159 160

22. How was the driver compensated for this trip?

Percent of gross trip revenue	[ ] 1
By hour	[ ] 2
By mile	[ ] 3
By hour & mile	[ ] 4
By load	[ ] 5
Other ______________ *(Specify)*	[ ] 6
	161

Compensation Method (editor)

__ __ __ __ __ __ __ __ __ __ __ __ __ __ __
162 163 164 165 166 167 168 169 170 171 172 173 174 175 176

23. How many hours had the driver been <u>driving</u> since the last 10-hour break?

__ __ Hours
177 178

24. Number of years of truck driving experience (for this driver):

__ __ Years
179 180

25. Number of years of driving for this company (for this driver):

__ __ Years
181 182

End of interview portion of survey...

See next page for final Editor Section of survey.

THIS ENTIRE PAGE TO BE COMPLETED BY EDITOR.

26. Collision avoidance section. Illustrate pre-collision scenario below.

Enter GES code: ___ ___ (183, 184)

27. Interview?

Yes [ ] 1
No [ ] 2
185

28. Police Report?

Yes [ ] 1
No [ ] 2
186

29. FAX/Mail/Email?

Yes [ ] 1
No [ ] 2
187

DERIVED INFORMATION *(Insert question numbers.)*

___ ___ (188, 189) ___ ___ (190, 191) ___ ___ (192, 193) ___ ___ (194, 195) ___ ___ (196, 197)

30. PAR Number

___ ___ ___ ___ ___ ___ ___ ___ ___ ___ ___ ___ (198 199 200 201 202 203 204 205 206 207 208 209)

31. DOT Number

___ ___ ___ ___ ___ ___ ___ (210 211 212 213 214 215 216)

32. State PSC Number

___ ___ ___ ___ ___ ___ ___ ___ ___ ___ (217 218 219 220 221 222 223 224 225 226)

33. State Issuing

___ ___ (227 228)

34. GVWR ___ (229)

35. GCWR ___ (230)
